# The phosphorylated trimeric SOSS1 complex and RNA polymerase II trigger liquid-liquid phase separation at double-strand breaks

**DOI:** 10.1016/j.celrep.2023.113489

**Published:** 2023-11-30

**Authors:** Qilin Long, Marek Sebesta, Katerina Sedova, Vojtech Haluza, Adele Alagia, Zhichao Liu, Richard Stefl, Monika Gullerova

**Affiliations:** 1 Sir William Dunn School of Pathology, University of Oxford, South Parks Road, Oxford OX1 3RE, UK; 2 Central European Institute of Technology (CEITEC), Masaryk University, 62500 Brno, Czech Republic; 3 National Center for Biomolecular Research, Faculty of Science, Masaryk University, 62500 Brno, Czech Republic

## Abstract

Double-strand breaks (DSBs) are the most severe type of DNA damage. Previously, we demonstrated that RNA polymerase II (RNAPII) phosphorylated at the tyrosine 1 (Y1P) residue of its C-terminal domain (CTD) generates RNAs at DSBs. However, the regulation of transcription at DSBs remains enigmatic. Here, we show that the damage-activated tyrosine kinase c-Abl phosphorylates hSSB1, enabling its interaction with Y1P RNAPII at DSBs. Furthermore, the trimeric SOSS1 complex, consisting of hSSB1, INTS3, and c9orf80, binds to Y1P RNAPII in response to DNA damage in an R-loop-dependent manner. Specifically, hSSB1, as a part of the trimeric SOSS1 complex, exhibits a strong affinity for R-loops, even in the presence of replication protein A (RPA). Our *in vitro* and *in vivo* data reveal that the SOSS1 complex and RNAPII form dynamic liquid-like repair compartments at DSBs. Depletion of the SOSS1 complex impairs DNA repair, underscoring its biological role in the R-loop-dependent DNA damage response.

## Graphical abstract

**Graphic abstract:**
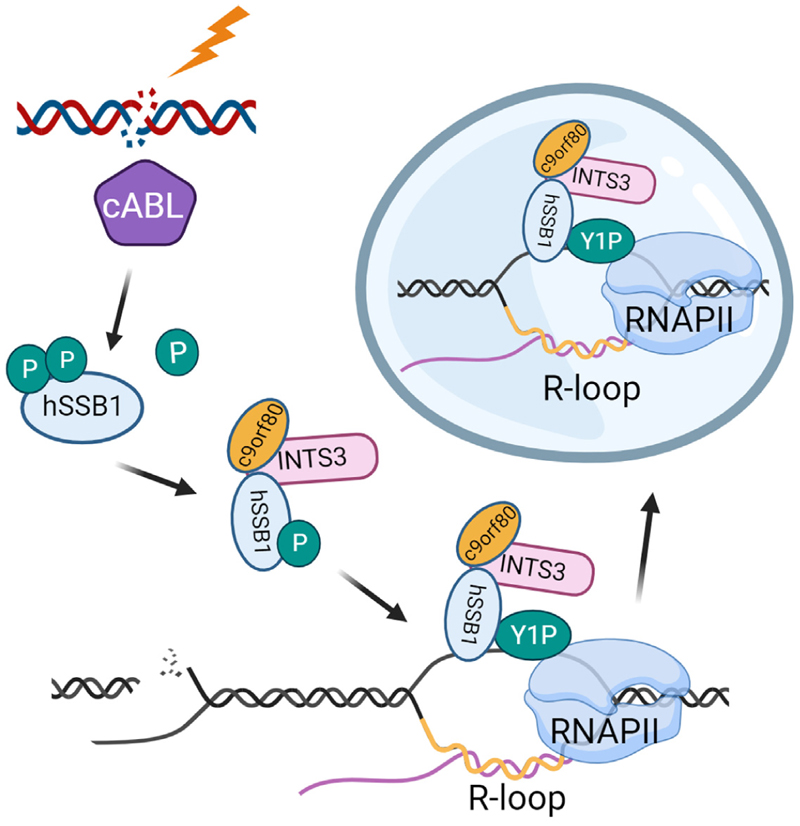

## Introduction

The stability of the human genome is challenged by numerous endogenous and exogenous insults.^1^ The DNA damage response (DDR) pathway safeguards genome integrity. The common types of DNA lesions include base conversion,^2^ bulky DNA addition,^3^ single-strand breaks,^4^ and double-strand breaks (DSBs).^5^ Persistent, unrepaired DSBs lead to chromosomal aberrations, genome instability, cell malfunction, or tumorigenesis.^6^

DSBs can be repaired by homologous recombination (HR) or non-homologous end joining (NHEJ).^5^ The HR repair pathway is initiated by resection of one of the DNA strands. Single-strand DNA binding (SSB) proteins protect the exposed single-stranded DNA (ssDNA) overhang. To date, four SSB proteins have been characterized: replication protein A (RPA), human SSB1 (hSSB1), human SSB2 (hSSB2), and mitochondrial SSB (mtSSB).^7,8^ RPA is well characterized and plays an essential role in almost all DNA metabolism pathways.^9^ In contrast, the role of the other SSBs in DDR is limited. Ataxia telangiectasia mutated (ATM)-phosphorylated hSSB1 on the threonine 117 residue has been implicated in HR and cell cycle regulation.^10^ hSSB1 can form a heterotrimeric sensor of ssDNA (SOSS) complex along with INTS3 and c9orf80, called the SOSS1 complex.^11,12^

Kinases are the key activators of DNA repair pathways. Specifically, three serine/threonine phosphatidylinositol 3-kinase-related kinase (PIKK) family members, ATM, ataxia telangiectasia and Rad3-related (ATR), and DNA-dependent protein kinase (DNA-PK) are critical upstream signal transducers.^13,14^ They are recruited and activated by protein complexes, such as MRN (MRE11/RAD50/NBS1), RPA/ATPIP, and KU70/KU80, respectively.^15^ Hundreds of proteins are directly phosphorylated in an ATM/ATR-dependent manner, particularly at Ser/Thr-Gln positions,^16,17^ leading to activation of the checkpoint transducers Chk1 and Chk2.^18,19^ DNA-PK prevents the end resection and phosphorylates NHEJ pathway factors.^20^ Besides PIKK members, the ubiquitously expressed Abelson tyrosine kinase (c-Abl) displays multifaceted roles in DDR.^21^ At DSBs, c-Abl phosphorylates the C-terminal domain (CTD) of the RNA polymerase II (RNAPII) at the tyrosine 1 (Y1) residue, which generates *de novo* damageresponsive transcripts (DARTs), required for efficient repair.^22^

R-loops are nucleic acid structures consisting of a DNA:RNA hybrid and the non-template ssDNA, usually occurring nearby RNAPII pausing sites. DSB-associated transcription generates R-loops, which serve as a binding platform for DDR factors to facilitate DNA repair.^22,23^

Liquid-liquid phase separation (LLPS) is an important mechanism required for the formation of membrane-less compartments, such as nucleoli, nuclear speckles, or RNA granules,^24,25^ as well as gene promoters and super-enhancers.^26^ During LLPS, part of a protein solution condenses into a dense phase, forming droplets with liquid-like properties, while the remaining solution forms a dilute phase.^27^ The driving force of LLPS is the weak multivalent interaction of intrinsically disordered regions.^28^ Accumulating evidence shows that LLPS promotes DNA damage repair.^29–31^

Here, we investigate the role of the trimeric SOSS1 complex in regulating transcription at DSBs. We show that the DNA damage-activated tyrosine kinase c-Abl phosphorylates hSSB1. p-hSSB1 binds to INTS3 and c9orf80, leading to formation of the trimeric SOSS1 complex. The formation of this complex is required for efficient binding of hSSB1 to R-loops, explaining the coexistence of RPA (a complex with substantially higher affinity toward ssDNA) and hSSB1 at DSBs. Furthermore, the trimeric SOSS1 complex and Y1P RNAPII trigger LLPS at DSBs to promote efficient DDR. The importance of our findings is further supported by the impaired DNA repair observed in cells lacking the trimeric SOSS1 complex. Thus, this study demonstrates the crucial role of the trimeric SOSS1 complex in formation of transient repair compartments and regulation of R-loop-dependent DDR.

## Results

### c-ABL phosphorylates hSSB1 upon DNA damage

**Figure 1:**
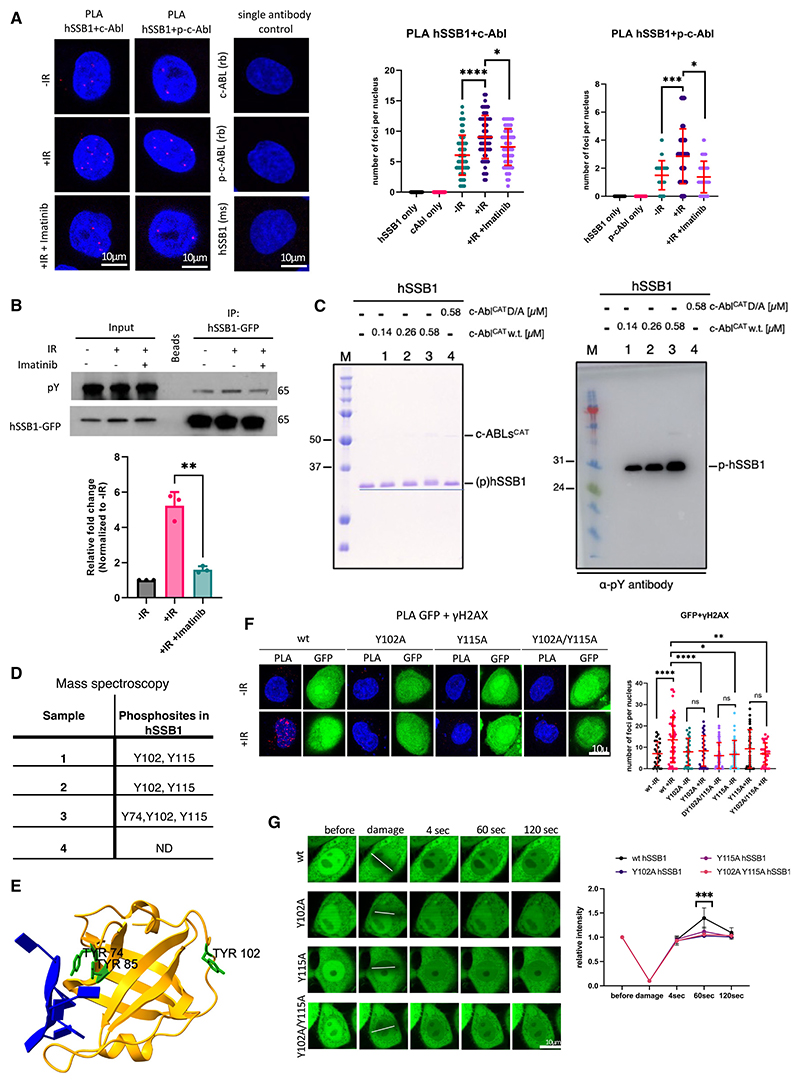
cAbl phosphorylates hSSB1 upon DNA damage (A) PLA of cAbl/p-cAbl and hSSB1 without IR, with IR, and with IR plus imatinib. IR = 10 Gy. n > 100. Left: representative confocal microscopy images. Right: quantification of left. Error bars, mean ± SD. Significance was determined using non-parametric Mann-Whitney test. *p ≤ 0.05, ***p ≤ 0.001, ****p ≤ 0.0001. A single antibody was used as a negative control. (B) Immunoprecipitation of hSSB1-GFP from cells subjected to IR and imatinib treatment. IR = 10 Gy. Top: immunoblots showing signals for pan-phosphotyrosine (α-pY) and hSSB1-GFP. Bottom: quantification of left. Error bars, mean ± SD. Significance was determined using paired t test. **p < 0.01. (C) In vitro phosphorylation of hSSB1 by cAbl^CAT^. hSSB1 was incubated with increasing concentrations of cAbl^CAT^ (0.14, 0.28, and 0.56 μM) or cAbl^CAT^ ^D363A^ (D/A) mutant at 0.56 mM for 30 min at 37°C and subsequently analyzed by SDS-PAGE and immunodetection with the α-pY antibody. Left: an SDS-PAGE gel of the reaction. Right: immunodetection by western blotting with an α-pY antibody. (D) Identification of the cAbl^CAT^ phosphorylation sites on hSSB1 by MS. The table shows identified residues in individual reactions. The sample number in the table corresponds to the numbering in (C). (E) Depiction of the position of tyrosine residues (in green) of hSSB1 (yellow) on the structural model with ssDNA (blue) (PDB: 4OWW). Residue Y115 is not visible/highlighted in the structure due to its absence in the structure. (F) PLA of GFP and γH2AX in cells transiently transfected with hSSB1^WT^-GFP or hSSB1^Y102A^-GFP, hSSB1^Y115A^-GFP and hSSB1^Y102A&Y115A^-GFP plasmids treated with or without IR. IR = 2 Gy. Left: representative confocal microscopy images. Right: quantification of left. Error bar, mean ± SD. Significance was determined using non-parametric Mann-Whitney test. *p ≤ 0.05, **p ≤ 0.01, ****p ≤ 0.0001. (G) Laser striping of cells transiently transfected with hSSB1^WT^-GFP or hSSB1^Y102A^-GFP, hSSB1^Y115A^-GFP and hSSB1^Y102A&Y115A^-GFP plasmids. Representative confocal microscopy images and quantification (n ≥ 10) show GFP signals before and after laser striping at the indicated time points. Error bars, mean ± SEM. Significance was determined using one-way ANOVA with a multiple-comparisons test. ***p ≤ 0.001. See also Figures S1−S3.

We have shown previously that c-Abl phosphorylates Y1P CTD RNAPII at DSBs, which leads to production of strand-specific DARTs.^22^ To investigate whether c-Abl phosphorylates components of the SOSS1 complex, we first performed a proximity ligation assay (PLA). This technique allows visualization of two proteins in close proximity (≤40 nm). Using antibodies against c-Abl and hSSB1, we detected PLA foci upon ionizing radiation (IR) treatment. The number of these foci was significantly reduced in the presence of the c-Abl inhibitor imatinib (Figure 1A; single antibodies were used as a negative control). Next, we repeated the PLA using an antibody against phosphorylated c-Abl (p-c-Abl) and detected an imatinib-sensitive interaction between p-c-Abl and hSSB1 (Figure 1A). Finally, we performed a co-immunoprecipitation (coIP) assay by pulling down hSSB1-GFP from cells with stably integrated hSSB1-GFP. Immunoblotting with a pan-phospho-tyrosine (α-pY) antibody revealed specific, imatinib-sensitive, damage-induced hSSB1 phosphorylation (Figure 1B).

To validate our *in vivo* data, we incubated purified hSSB1 (Figures S1A and S1B) with the catalytic domain of c-Abl (amino acids (aas) 83−534, c-Abl^CAT^) or its kinase-dead variant (c-Abl^CAT D363A^) *in vitro*. Upon SDS-PAGE analysis, we observed a shift in the bands corresponding to hSSB1 protein, which correlated with increasing concentration of c-Abl^CAT^ but not with the catalytic mutant (Figure 1C, left). To confirm that the shift in the protein band on the gel was indeed caused by phosphorylation by c-Abl^CAT^, we performed immunoblotting with the α-pY antibody and detected a signal that corresponded to the size of hSSB1 protein (Figure 1C, right). As a control, we incubated c-Abl^CAT^ and c-Abl^CAT D363A^ with an unrelated protein: glutathione S-transferase (GST). As expected, no phosphorylation of GST was observed (Figures S1C and S1D). Next, we subjected the bands from the SDS-PAGE gel (Figure 1C) to mass spectrometry (MS) (Table S1). The MS analysis identified phosphorylation of hSSB1 on residues Y102, Y115, and Y74, among which the phosphorylation on Y102 and Y115 was present in every sample (Figure 1D). We visualized the position of the phosphorylated tyrosine residues within the hSSB1 protein structure. Interestingly, Y74 and the previously identified Y85^32^ residue are located on the ssDNA binding interface, while the Y102 residue is present on the hSSB1-INTS3 interface. The Y115 residue is in a flexible region of hSSB1 and, hence, not visible in the model (Figures 1E and S1E).

Taken together, our *in vivo* and *in vitro* experiments show that DNA damage significantly increases c-Abl-mediated phosphorylation of tyrosine residues on hSSB1.

### hSSB1 phosphorylation is required for its localization to DSBs

To test whether c-Abl-mediated phosphorylation of hSSB1 plays a role in DDR, we investigated the recruitment of stably integrated hSSB1-GFP to sites of laser-induced damage *in vivo*. First, we detected hSSB1-GFP to be rapidly (32 s after laser damage) recruited to DSBs. This recruitment was impaired in the presence of imatinib (Figure S2A). Next, we performed a PLA in HeLa cells using antibodies against hSSB1, c-Abl, and γH2AX and observed that, even in presence of imatinib, there was a significant increase in hSSB1/γH2AX and c-Abl/γH2AX PLA signals. This was most likely caused by incomplete inhibition of c-Abl activity by the imatinib treatment. However, the imatinibdependent reduction of the interaction of hSSB1 and c-Abl with γH2AX was significant upon IR treatment (Figures S2B and S2C).

Next, we transiently transfected cells with plasmids expressing the hSSB1-GFP wild type (WT), hSSB1^Y102A^-GFP, hSSB1^Y115A^-GFP, and hSSB1^Y102A/Y115A^-GFP variants (Figure S3A) and observed positive PLA foci of GFP (detecting hSSB1-GFP variants) and γH2AX in cells expressing hSSB1 WT after IR treatment. Meanwhile, the number of foci was significantly reduced in cells expressing all three mutants (Figure 1F). It should be noted that the baseline signal in non-irradiated samples was the same in all tested samples. Additionally, we performed a PLA assay using hSSB1 and γH2AX antibodies (Figure S3B) and detected PLA foci in cells transfected with hSSB1 WT, and these were significantly reduced in cells expressing the mutants. Next, we generated stable cell lines expressing hSSB1-GFP WT, hSSB1^Y102A^-GFP, hSSB1^Y115A^-GFP, and hSSB1^Y102A/Y115A^-GFP mutants and subjected them to laser striping. Laser-induced DNA damage led to rapid recruitment of hSSB1-GFP WT but not of the mutants to DSBs (Figure S3C). Similarly, laser striping of transiently transfected cells resulted in the recruitment of hSSB1-GFP WT but not of the mutants to DSBs (Figure 1G).

Overall, the data demonstrate that the DNA damage-induced, c-Abl-mediated phosphorylation of hSSB1 on Y102 and Y115 residues is required for its recruitment to DSBs.

### Localization of hSSB1 to DSBs is R-loop dependent *in vivo*

**Figure 2:**
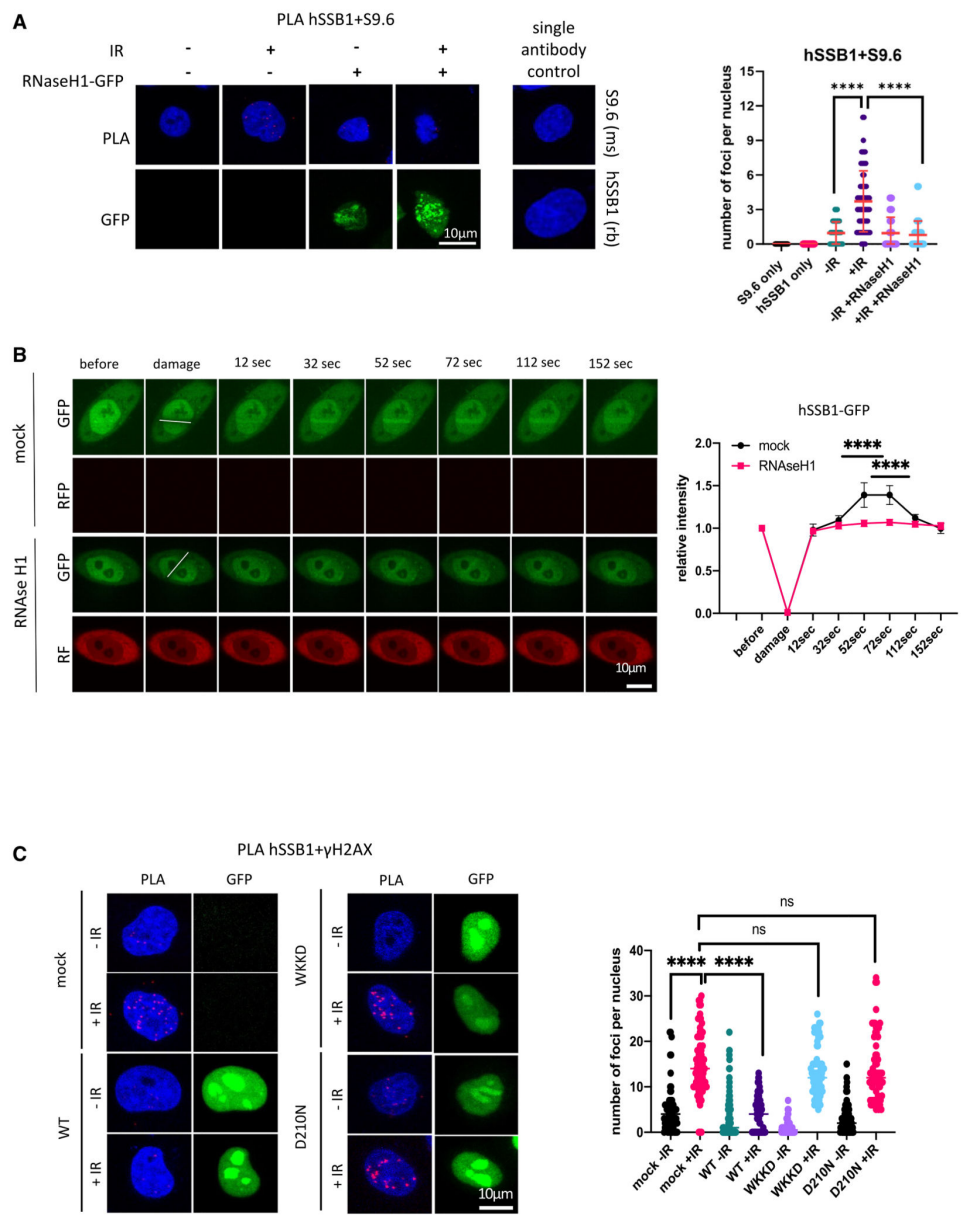
Localization of hSSB1 to DSBs is R-loop dependent (A) PLA of hSSB1 and S9.6 (R-loops) with or without IR in the presence or absence of RNase H1. IR = 10 Gy. Left: representative confocal microscopy images. Right: quantification of left. Error bars, mean ± SD. Significance was determined using non-parametric Mann-Whitney test. ****p ≤ 0.0001. A single antibody was used as a negative control. (B) Laser striping of stably integrated hSSB1-GFP cells with or without transient expression of the RNase H1-RFP plasmid. Representative confocal microscopy images and quantification (n R 10) show GFP and RFP signals at the indicated time points. Error bars, mean ± SEM. Significance was determined using multiple unpaired Student’s t tests. ****p ≤ 0.0001. (C) PLA of hSSB1 and γH2AX in cells transiently transfected with RNase H1^WT^-GFP or RNaseH1^WKKD^-GFP (binding and catalytic) or RNaseH1^D210N^-GFP (catalytic) mutants with or without IR. IR = 2Gy. Top: representative confocal microscopy images. Bottom: quantification of left. Error bars, mean ± SD. Significance was determined using non-parametric Mann-Whitney test. ****p ≤ 0.0001. See also Figure S4.

R-loops are transcription-dependent structures found near DSBs.^22,33,34^ To test whether hSSB1 directly binds to R-loops, we first performed a modified PLA^35^ using antibodies recognizing hSSB1 and RNA:DNA hybrids (S9.6 also recognizing R-loop structures) and observed a significant increase in RNase H1-sensitive PLA foci upon IR treatment (Figure 2A). To test whether R-loops might act as a binding platform for hSSB1, we transiently transfected hSSB1-GFP cells with plasmids expressing ribonuclease RNase H1, an enzyme specifically degrading RNA:DNA hybrids, before subjecting them to laser striping (Figure 2B). First, we confirmed a rapid (from 12 s) recruitment of hSSB1 to DSBs in control cells. The hSSB1-GFP signal was significantly reduced when RNase H1 was overexpressed. Expression of the catalytically inactive variants RNase H1^D210N^ (cannot degrade R-loops) and RNase H1^WKKD^ (cannot degrade nor bind to R-loops) did not affect hSSB1 recruitment to DSBs. We also tested recruitment of RNase H1-GFP WT, RNase H1^D210N^-GFP, and RNase H1^WKKD^-GFP to DSBs by a PLA and confirmed a proximity of RNase H1-GFP to γH2AX in cells transfected with the RNase H1-GFP WT and RNase H1^D210N^-GFP variant but not with RNase H1^WKKD^-GFP (Figure S4). Subsequently, we observed a significant reduction in PLA foci corresponding to hSSB1 and γH2AX upon overexpression of RNase H1-GFP WT, which was not observed upon overexpression of its catalytic variants (Figure 2C).

Collectively, our data suggest that the recruitment of hSSB1 (and, *in extenso*, the trimeric SOSS1 complex) to the sites of DNA damage *in vivo* is mediated by R-loops.

### The trimeric SOSS1 complex overcomes the inhibitory effect of RPA in hSSB1-mediated binding to R-loops

**Figure 3:**
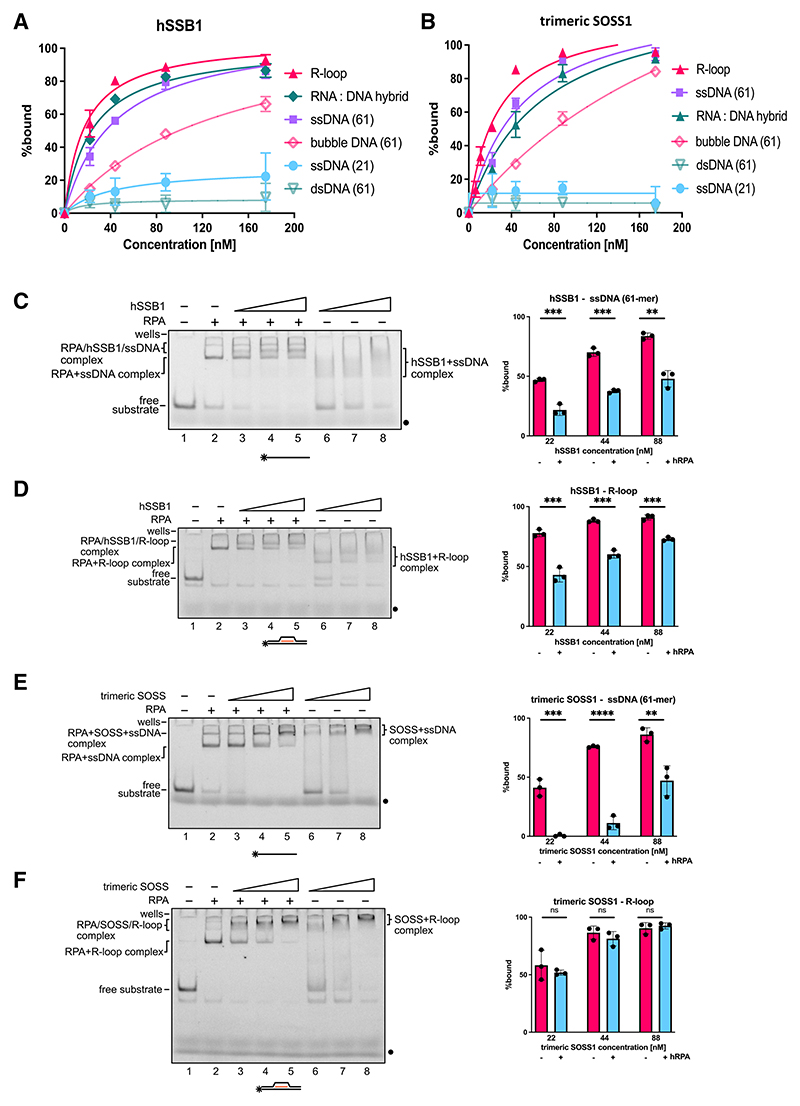
The trimeric SOSS1 complex suppresses the inhibitory effect of RPA in hSSB1-mediated binding of R-loops (A) Graph representing quantification of EMSA experiments (n = 3) conducted between hSSB1 and R-loop, RNA:DNA hybrid, 61-mer ssDNA, bubble DNA, 21-mer DNA, and 61-mer dsDNA, respectively. (B) As in (A) with the trimeric SOSS1. (C) Scan of representative EMSA experiments (left) and bar chart (right) representing quantification of EMSA experiments (n = 3) conducted between hSSB1 (at 22, 44, and 88 nM) and ssDNA (61-mer) in the absence or presence of 30 nM RPA. Error bars, mean ± SD. Significance was determined using unpaired Student’s t test. **p ≤ 0.01 and ***p ≤ 0.001. (D) Scan of representative EMSA experiments (left) and bar chart (right) representing quantification of EMSA experiments (n = 3) conducted between hSSB1 (at 22, 44, and 88 nM) and the R-loop in the absence or presence of 30 nM RPA. Error bars, mean ± SD. Significance was determined using unpaired Student’s t test. ***p ≤ 0.001. (E) As in (C) with the trimeric SOSS1. **p < 0.01, ***p < 0.001, ****p < 0.0001. (F) As in (D) with the trimeric SOSS1. See also Figures S5−S12.

hSSB1, together with RPA, belongs to the SSB protein family.^7^ Given the high affinity of RPA to ssDNA, it was unclear how hSSB1 and RPA might coexist at DSBs. Additionally, ssDNA is also a component of R-loops.^36,37^ We performed a comprehensive binding analysis of the trimeric SOSS1 and its subunits to a broad range of nucleic acid (NA) substrates (21-nt ssDNA, 61-nt ssDNA, 61-nt ssRNA, 61-nt dsDNA, DNA/RNA hybrid, R-loop, and DNA bubble) *in vitro*. By using an electrophoretic mobility shift assay (EMSA), we first determined the binding preferences of hSSB1 to NA substrates. As expected from the literature,^10^ hSSB1 bound ssDNA in a length-dependent manner; hSSB1 did not bind to 21-nt ssDNA while exhibiting high affinity toward 61-nt ssDNA (Figures 3A and S5A−S5C). Surprisingly, hSSB1 bound to R-loop structures and RNA:DNA hybrids, but not to bubble DNA, with an affinity similar to that of 61-nt ssDNA (Figures 3A and S5D−S5F). Additionally, hSSB1 bound to ssRNA with an affinity similar to ssDNA (Figures S5G and S5H). Because the ssDNA portion within R-loop structures is 21 nt long (which is not bound by hSSB1 in isolation), it suggests that hSSB1 recognizes R-loops specifically. The trimeric SOSS1 complex exhibited affinities to the NA substrates comparable with hSSB1 (Figures 3B and S6A−S6H). However, INTS3 alone did not bind to any of the structures (Figures S7A−S7D). Therefore, we conclude that hSSB1, alone or as a subunit of the trimeric SOSS1, binds to NA substrates with a preference for R-loops.

Additionally, hSSB1^Y102A^, hSSB1^Y115A^, and hSSB1^Y102A/Y115A^ mutants could bind to ssDNA and R-loops to a similar extent as hSSB1 WT. In contrast, hSSB1^Y74A^ and hSSB1^Y85A^ mutants bound significantly less to ssDNA than hSSB1 WT. Furthermore, binding of the hSSB1^Y74A^ mutant to R-loops was also reduced (Figures S8A−S8J and S9A−S9D).

To investigate the NA binding of the trimeric SOSS1 complex and hSSB1 in the presence of RPA, we performed a set of competitive EMSA experiments. We first pre-incubated the NA substrates with RPA at either 10 or 30 nM and then included hSSB1 or trimeric SOSS1 complex. RPA significantly reduced the binding of hSSB1 to ssDNA (61 nt), RNA:DNA hybrids, and R-loop substrates (Figures 3C, 3D, and S10A−S10D). In contrast, RPA significantly reduced binding of the trimeric SOSS1 complex only to ssDNA (Figures 3E and S11A), but it did not inhibit SOSS1 binding to R-loop structures (Figures 3F and S11B) or to RNA:DNA hybrids (Figure S11C). SOSS1 did not bind to short, 21-nt ssDNA (Figure S11D). Because RPA does bind to short, 21-nt ssDNA, it exhibits a binding affinity similar to all tested substrates except bubble DNA (Figures S12A−S12H). This suggests that RPA is coating the ssDNA portions of the substrates and that hSSB1, either alone or embedded in the trimeric SOSS1 complex, recognizes the branched portions of the substrates.

We conclude that the association of hSSB1 with the other subunits of the trimeric SOSS1 complex is required to overcome the inhibitory effect of RPA on its binding to R-loops and RNA:DNA hybrids. This notion is consistent with the hypothesis that RPA preferentially coats resected ssDNA, while the SOSS1 complex primarily recognizes R-loops behind RNAPII near DSBs.

### The trimeric SOSS1 complex binds to the CTD of RNAPII upon DNA damage

**Figure 4:**
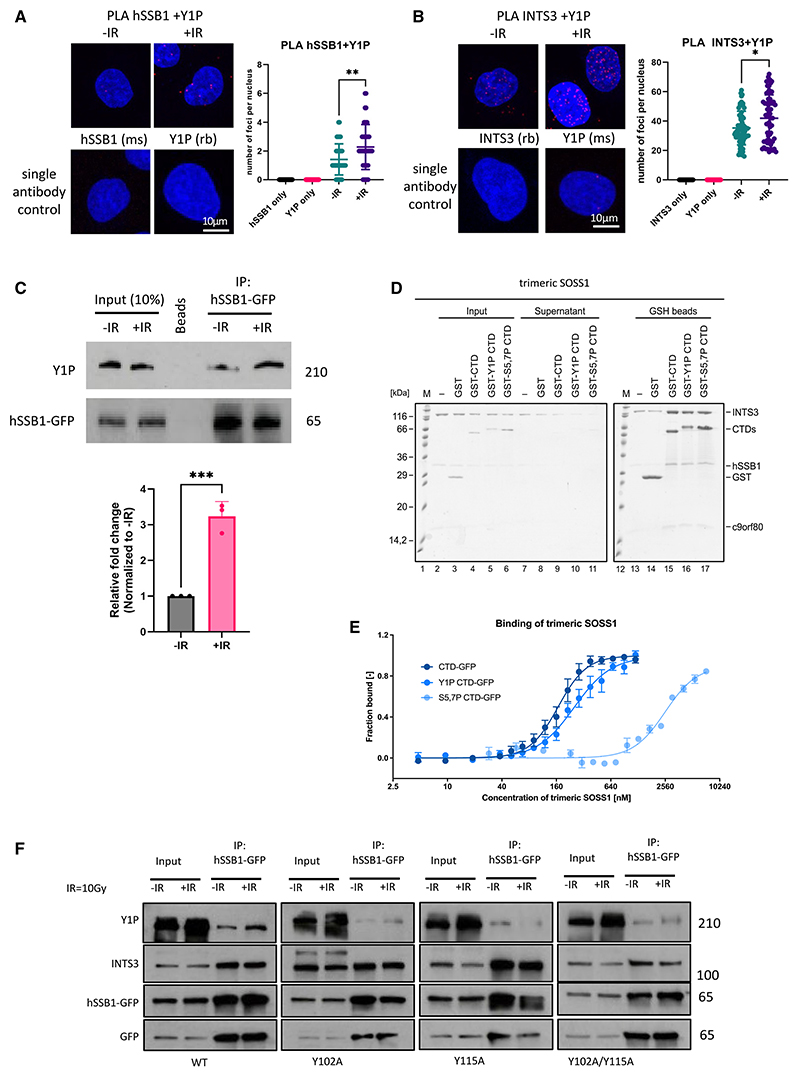
The trimeric SOSS1 complex binds to the Y1P CTD of RNAPII upon DNA damage (A) PLA of hSSB1 and Y1P in HeLa cells with or without IR. IR = 10 Gy. Left: representative confocal microscopy images. Right: quantification of left. Error bars, mean ± SD. Significance was determined using non-parametric Mann-Whitney test. **p ≤ 0.01. A single antibody was used as a negative control. (B) PLA of INTS3 and Y1P in cells with or without IR. IR = 10 Gy. Left: representative confocal microscopy images. Right: quantification of left. Error bars, mean ± SD. Significance was determined using non-parametric Mann-Whitney test. *p ≤ 0.05. A single antibody was used as a negative control. (C) Co-immunoprecipitation of hSSB1-GFP followed by immunoblotting using GFP and Y1P RNAPII antibodies. IR = 10 Gy. Samples were collected 1 h post IR. Bottom: quantification of left. Error bars, mean ± SD. Significance was determined using paired t test. ***p ≤ 0.001. (D) In vitro pull-down assay of the trimeric SOSS1 complex with immobilized GST-CTD, GST-CTD phosphorylated on Tyr1 (GST-Y1P-CTD), or GST-CTD phosphorylated on Ser5 and Ser7 (GST-S5,7P-CTD). (E) Microscale thermophoresis (MST) binding curves of the trimeric SOSS1 complex with unmodified CTD-GFP, Y1P-CTD-GFP, or S5,7P-CTD-GFP. Measured in triplicates; the lines represent the Hill fit. (F) CoIP of hSSB1-GFP from stably integrated hSSB1^wt^-GFP or hSSB1^Y102A^-GFP, hSSB1^Y115A^-GFP and hSSB1^Y102A&Y115A^-GFP cells with or without IR treatment. IR = 10 Gy. Samples were collected 1 h post IR. Immunoblots show signals for Y1P RNAPII, INTS3, GFP, and hSSB1. See also Figure S13.

Previously, we have shown that Y1P RNAPII actively transcribes RNA at DSBs.^22^ Under non-damage conditions, Y1P RNAPII is mostly detected at the start of genes and with the antisense orientation.^38^ Furthermore, MS analysis suggested that the SOSS1 complex can associate with Y1P RNAPII.^39^ To investigate the interaction between the trimeric SOSS1 complex and RNAPII, we used a PLA and detected a significant increase in the number of foci when using antibodies recognizing hSSB1 or INTS3 and Y1P RNAPII coupled with IR treatment (Figures 4A and 4B; single antibodies were used as negative controls). To complement our PLA data, we performed coIP experiments by pulling down hSSB1-GFP in cells exposed to IR treatment and immunoblotted for Y1P RNAPII. We observed increased levels of Y1P RNAPII in hSSB1-GFP pull-down experiments after IR, which is consistent with the PLA experiments (Figure 4C). Next, we performed *in vitro* pull-down experiments with purified SOSS1 complex and GST-CTD (unmodified), phosphorylated on Tyr1 (Y1P CTD), or phosphorylated on Ser5 and Ser7 (S5,7P CTD) polypeptides. All three tested variants of GST-CTD efficiently and specifically pulled down the trimeric SOSS1 complex (Figure 4D). To determine which subunit of the trimeric SOSS1 complex is responsible for binding to the CTD of RNAPII, we repeated the pull-down experiments with individual subunits (Figures S13A and S13B) but failed to detect an interaction with CTD polypeptides. Intriguingly, the trimeric SOSS1 complex assembled from individual, purified subunits failed to interact with CTD polypeptides as well (Figure S13C), suggesting that proper complex assembly is required for binding of the trimeric SOSS1 complex to the CTD of RNAPII. To gain quantitative insight into the binding of the trimeric SOSS1 complex to CTD polypeptides, we performed microscale thermophoresis (MST), which revealed that the trimeric SOSS1 showed higher affinity to unphosphorylated and Y1P CTD than to S5,7P CTD (Figure 4E).

Next, we asked whether hSSB1 phosphorylation can affect its ability to bind to Y1P RNAPII or INTS3. We performed co-immunoprecipitation of hSSB1-GFP WT, hSSB1^Y102A^-GFP, hSSB1^Y115A^-GFP, and double hSSB1^Y102A/Y115A^-GFP mutants, followed by immunoblotting using antibodies recognizing Y1P or INTS3. We observed that hSSB1 mutants did not bind Y1P RNAPII as efficiently as hSSB1 WT, but their binding to INTS3 was not affected (Figure 4F). Next, we purified the trimeric SOSS1 complexes harboring hSSB1^Y102A^, hSBB1^Y115A^, and hSSB1^Y102A/Y115A^ and tested their ability to directly bind the Y1P CTD *in vitro*. Intriguingly, all SOSS1 complexes bound the Y1P CTD (Figure S13D). We hypothesize that the interaction between SOSS1 and RNAPII may take place only at sites of DSBs. Because the recruitment of the hSSB1 to DSBs is abrogated by loss of phosphorylation, it may not interact with Y1P RNAPII *in vivo*.

### Nucleic acids and the CTD of RNAPII promote phase separation of the trimeric SOSS1 complex into condensates *in vitro*

**Figure 5:**
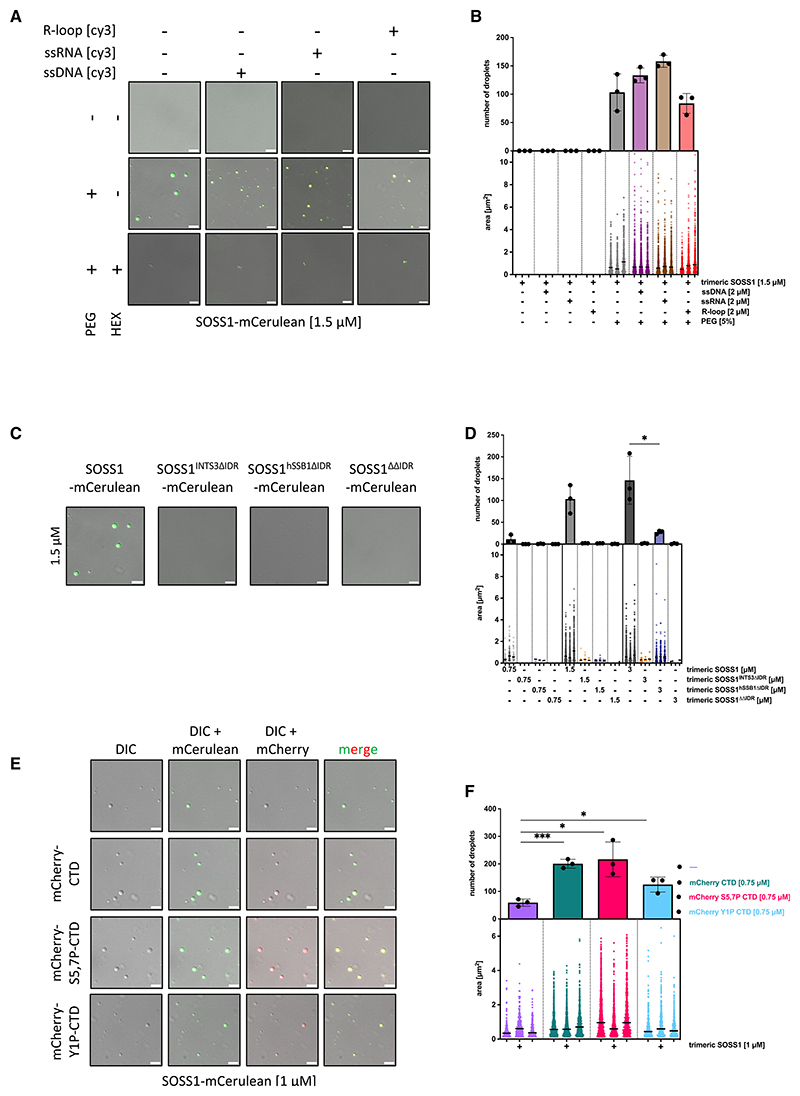
Phase separation of the trimeric SOSS complex is promoted by the DNA and CTD of RNAPII *in vitro* (A) LLPS experiments of purified trimeric, fluorescently labeled SOSS1 complex (on the INTS3 subunit), determining the effect of a crowding agent (5% PEG-8000), ssDNA (2 μM), ssRNA (2 μM), and the R-loop (2 μM) on the efficiency of phase separation of the complex. Representative images from three experiments are depicted as an overlay of differential interference contrast (DIC), mCerulean, and cy3 (where NAs are present) channels. Hexane-1,6-diol (HEX; at 10%) was added to inhibit hydrophobic interactions. Scale bars, 5 μm. polyethylene glycol (PEG), PEG-8000. (B) Bar chart (top) representing quantification (n = 3) of the number of droplets from the LLPS experiments shown in (A). Statistical significance was determined by unpaired t test. A nested scatterplot (bottom) represents quantification (n = 3) of an area of individual droplets from three independent experiments shown in (A), with median area determined per dataset. Statistical significance was determined by nested t test. (C) Determination of the domain responsible for LLPS of the trimeric SOSS1 complex. Representative images from three experiments with WT, mCerulean-labeled (on INTS3) SOSS1 complex, and its mutant variants (all at 1.5 μM) with deleted IDRs found within INTS3 (aas 959−1,042, INTS3^ΔIDR^), hSSB1 (aas 140−212, hSSB1^ΔIDR^), and combination of both deletions (SOSS1^Δ Δ IDR^). The images are depicted as an overlay of DIC and mCerulean channels. Scale bars, 5 μM. (D) Bar chart (top) representing quantification (n = 3) of the number of droplets from the LLPS experiments shown in (C). Statistical significance was determined by unpaired t test. A nested scatterplot (bottom) represents quantification (n = 3) of an area of individual droplets from three independent experiments shown in (C), with median area determined per dataset. Statistical significance was determined by nested t test. *p < 0.05. (E) LLPS experiments investigating the effect of mCherry-labeled CTDs (unmodified, S5,7P, and tyrosine 1 Y1P at 0.75 μM) on phase separation with the trimeric SOSS1 complex, labeled with mCerulean on INTS3 (1 μM). Representative images from three experiments are depicted as DIC, overlay of DIC and mCerulean, overlay of DIC and mCherry, and overlay of all three channels. Scale bars, 5 μM. (F) Bar chart (top) represents quantification (n = 3) of the number of droplets from the LLPS experiments shown in (E). Statistical significance was determined by unpaired t test. *p ≤ 0.05 and ***p ≤ 0.001. A nested scatterplot (bottom) represents quantification (n = 3) of an area of individual droplets from three independent experiments shown in (E), with median area determined per dataset. Statistical significance was determined by nested t test. See also Figures S14 and S15.

Next, we investigated the role of the trimeric SOSS1 complex at DSBs. Previous structural work^40^ suggested that the C termini of INTS3 and hSSB1 are largely unstructured, intrinsically disordered regions (IDRs). IDRs can drive LLPS.^41,42^ To test whether the trimeric SOSS1 complex is able to phase separate, we purified the trimeric SOSS1 complex with an mCerulean fluorescent protein tag fused to the C terminus of INTS3. The trimeric SOSS1 complex alone, in the presence of ssDNA, ssRNA, or R-loops, did not phase separate at physiological salt concentration. When a crowding agent (5% polyethylene glycol [PEG]-8000) was included, we observed a robust, concentration-dependent appearance of condensates, which were sensitive to hexane-1,6-diol (HEX) and are characteristic of LLPS. HEX is an aliphatic alcohol that interferes with hydrophobic interactions and consequently dissolves condensates driven by hydrophobic interactions. Furthermore, addition of ssDNA, ssRNA, and R-loops resulted in a significant increase in the number and size of the condensates, suggesting that these NA structures may promote LLPS of the trimeric SOSS1 complex (Figures 5A, 5B, and S14A−S14D).

To identify which region of the trimeric SOSS1 complex is responsible for the phase separation *in vitro*, we constructed a set of variants of the trimeric SOSS1 complex in which the IDR regions of INTS3 (aas 959−1,043) and hSSB1 (aas 140−212) were deleted individually or in combination, creating variants SOSS1^INTS3ΔIDR^, SOSS1^hSSB1ΔIDR^, and SOSS1^ΔΔIDR^

(Figures S15A and S15B). When these variants were tested across various concentrations, SOSS1^INTS3ΔIDR^ and SOSS1^ΔΔIDR^ did not phase separate, while SOSS1^hSSB1ΔIDR^ exhibited a severely reduced ability to phase separate *in vitro* (Figures 5C, 5D, and S15A). These data suggest that the IDR domain of INTS3 is essential, while the IDR domain of hSSB1 is important, but not essential, for efficient phase separation of the trimeric SOSS1 complex.

Next, we generated a variant of the trimeric SOSS1 complex in which the hSSB1 subunit was also tagged with a different fluorescent tag (mOrange) alongside mCerulean-tagged INTS3. We observed that such a complex can indeed phase separate (Figures S14E and S14F), albeit to a lesser extent compared with the trimeric SOSS1 complex tagged only on the INTS3 subunit (Figure S14F). In the observed droplets, we did detect a signal for both INTS3 and hSSB1, suggesting that the entire complex phase separated into droplets.

We next wondered whether the trimeric SOSS1 complex may form heterotypic condensates, thereby serving as a scaffold for additional proteins. Given that it is widely accepted that RNAPII, via its CTD, may be one of such proteins,^43–45^ we tested this hypothesis by combining the trimeric SOSS1 complex with CTD polypeptides tagged with mCherry and either unmodified, S5,7P-CTD, or Y1P-CTD. While the S5,7P-CTD and Y1P-CTD polypeptides efficiently entered the condensates, the unmodified CTD entered to a lesser extent (Figure 5E). Importantly, all three forms of the CTD peptides promoted phase separation of the trimeric SOSS1 complex (Figure 5F). This effect is not caused by phase separation of the CTD itself because, under the tested conditions, none of the CTD peptides phase separated (Figure S15C). Next, we investigated whether both ssDNA and CTD polypeptides may enter the same condensates. We used unmodified trimeric SOSS1 complex and show that both S5,7P CTD tagged with mGFP and ssDNA entered the same condensates (Figure S15D and S15E).

Collectively, our results suggest that the trimeric SOSS1 complex efficiently phase separates *in vitro*, in the presence of the RNAPII and various NA structures (ssDNA, ssRNA, and R-loops).

### hSSB1 and INTS3 form condensates with liquid-like properties at the sites of DSBs *in vivo*

To test whether the trimeric SOSS1 complex forms condensates with liquid-like properties *in vivo*, we used the optoDroplet system, which is an optogenetic tool that uses the photolyase homology region (PHR) of *Arabidopsis thaliana*, Cry2. After fusing a protein of interest with Cry2, the potential of proteins to undergo phase separation can be evaluated upon light stimulation.^46^

First, we cloned the full-length hSSB1 and INTS3 into mCherry-PHR (Cry2) plasmids.^46^ Also, we used Cry2 WT alone and Cry2 fused to the IDRs of FUS and hnRNPA1 as negative and positive controls, respectively.^46^ After stably integrating all constructs into HeLa cells, we tested whether the cells could form optoDroplets when subjected to light induction (Figure S16A). No conden-sate formation was detected upon light induction in cells expressing Cry2 alone (Figure S16B; Video S1). Fusion of FUS and hnRNPA1 IDRs with Cry2 resulted in time-dependent opto-Droplet formation, as shown previously,^46^ suggesting that the optoDroplet system works in our hands (Figures S16C and S16D; Videos S2 and S3). Interestingly, we also observed lightinduced optoDroplet formation in cells expressing Cry2-tagged hSSB1 and INTS3 (Figures S16E and S16F; Videos S4 and S5).

**Figure 6:**
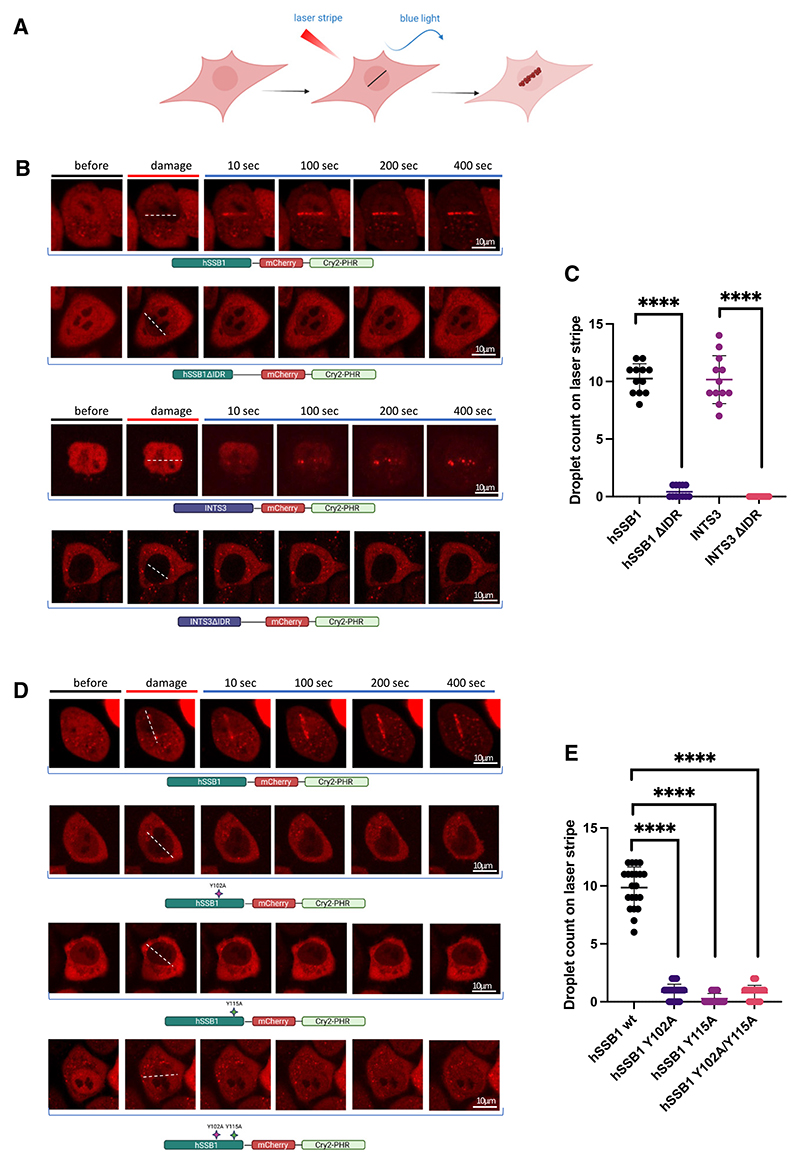
SOSS1 phase separates at doublestrand breaks *in vivo* (A)The optoDroplet strategy combined with laser striping. (B) Damage-induced optoDroplet formation of hSSB1-Cry2-mCherry, hSSB1^ΔIDR^ (Δaa140−211)-Cry2-mCherry, INTS3-Cry2-mCherry, and INTS3^Δ IDR^(Δaa959−1,042)-Cry2-mCherry cells. Shown are representative images of optoDroplets before and after laser striping and during light induction at the indicated time points. The position of the laser stripe is marked with a dashed white line. (C) Quantification of optoDroplets from 3 independent experiments shows values for optoDroplet numbers on the laser stripe 400 s after light induction. Significance was determined by Student’s t test. ****p ≤ 0.0001. (D) Damage-induced optoDroplet formation of hSSB1-Cry2-mCherry, hSSB1^Y102A^ -Cry2-mCherry, hSSB1^Y115A^-Cry2-mCherry, and hSSB1^Y102A,Y115A^-Cry2-mCherry cells. Shown are representative images of optoDroplets before and after laser striping and during light induction at the indicated time points. The position of the laser stripe is marked with a dashed white line. (E) Quantification of optoDroplets from 3 independent experiments shows values for optoDroplet numbers on the laser stripe 400 s after light induction. Significance was determined Student’s t test. ****p ≤ 0.0001. See also Figures S16 and S17.

A recent study showed that the CTD of RNAPII can phase separate to form hubs at actively transcribed genes.^26^ We showed that the SOSS1 complex interacts with RNAPII upon DNA damage (Figure 4). Additionally, it has been proposed that, at DSBs, LLPS may integrate DNA repair factors into specific compartments to increase the efficiency of repair.^30^ Therefore, we investigated whether the trimeric SOSS1 complex might contribute to condensate formation at DSBs. We modified the light-inducible optoDroplet protocol and included DNA damage induction by using laser striping (at 405 nm) prior to light induction (Figure 6A). Cry2 WT, Cry2-FUS-IDR, and Cry2-hnRNPA1-IDR were not recruited to the laser stripes, suggesting that an IDR domain alone is not sufficient for DSB recruitment (Figures S17A−S17D; Videos S6, S7, and S8). In contrast, both Cry2-hSSB1 and Cry2-INTS3 were rapidly recruited to DSBs and formed condensates in a time-dependent manner within the laser stripe area (Figures 6B and 6C; Videos S9 and S10; see also Figure 2B for comparison with hSSB1 recruitment to DSBs without OptoDroplet fusion). Droplet formation was not observed when the IDR domains in hSSB1 and INTS3 were individually deleted (Figures 6B and 6C; Videos S11 and S12), indicating that the IDR domains of hSSB1 and INTS3 are the drivers for their droplet formation *in vivo*.

Additionally, we generated cells stably expressing the opto-Droplet hSSB1^Y102A^, hSSB1^Y115A^, and hSSB1^Y102A/Y115A^ variants. We found no condensate formation in cells expressing mutant hSSB1 (Figures 6D and 6E; Videos S13, S14, S15, and S16), which is in agreement with our previous observation that hSSB1 phos-pho-mutants are not recruited to laser-induced DNA damage sites (Figure 1G and S3C).

Together, these data suggest that the trimeric SOSS1 complex contributes to phase separation at DSBs via the IDR domains and hSSB1 phosphorylation.

### Trimeric SOSS1 is required for efficient DNA damage repair

**Figure 7:**
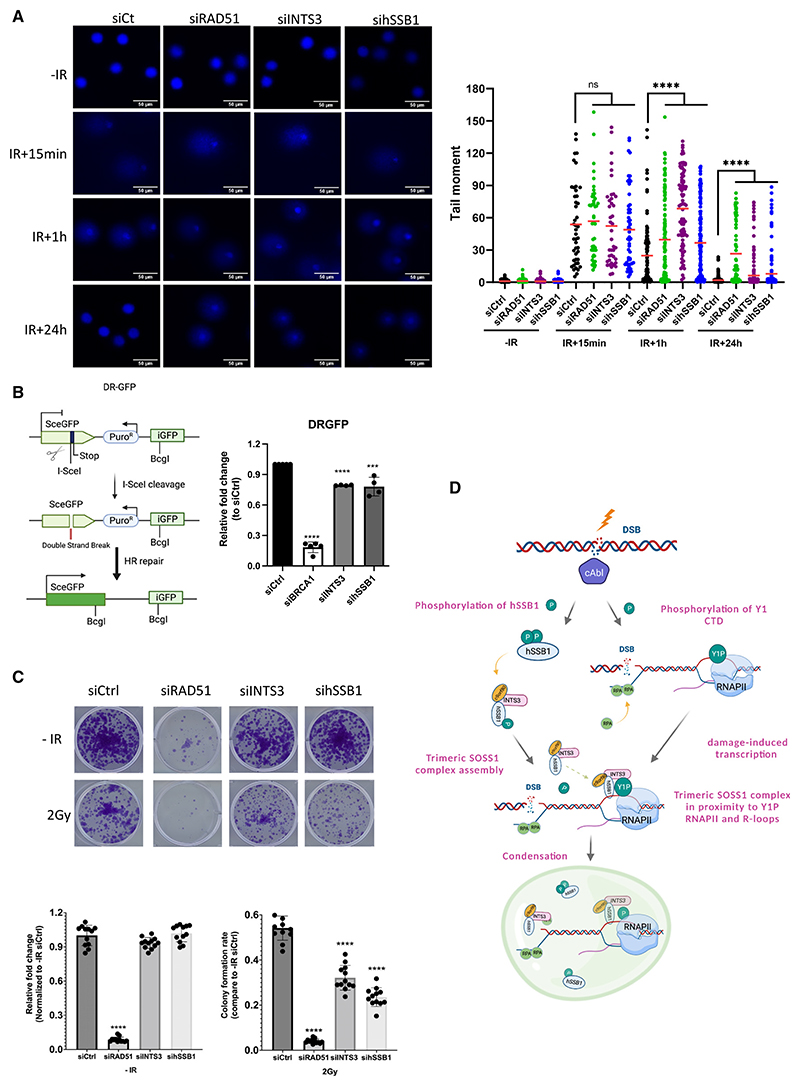
SOSS1 is required for efficient DNA repair (A) Comet assay was performed to visualize DNA breaks after downregulation of INTS3 and hSSB1 by small interfering RNA (siRNA) in the presence or absence of IR treatment. IR = 5 Gy. Samples were collected at the indicated time points. Downregulation of RAD51 served as a positive control. Right: quantification of the left. Error bar, mean ± SD. Significance was determined using unpaired Student’s t test. ****p ≤ 0.0001. (B) DR-GFP HeLa HR reporter assay. Left: schematic of the DR-GFP HeLa HR reporter assay. Right: bar chart representing FACS data determining the efficiency of HR repair after knockdown of the SOSS1 complex. Knockdown of BRCA1 served as a positive control. The significance was determined by Student’s t test. *** p ≤ 0.001, ****p ≤ 0.0001. (C) Top: representative images from the clonogenic assay. HeLa cells with or without IR treatment (2 Gy) after siCtrl, siRAD51, siINTS3, and sihSSB1 knockdown. siRAD51 worked as a positive control. The cells were stained and counted after 10 days of growing. Bottom: quantification of top. Error bars, mean ± SD. Significance was determined using unpaired Welch’s correction. ****p ≤ 0.0001. (D) Model. In response to DNA damage, damage-activated c-abl phosphorylates hSSB1 on Y102 and Y115 (p-hSSB1). Phosphorylated Y1P CTD RNAPII subsequently generates DARTs at DSBs.^22^ p-hSSB1 binds to INTS3 and c9orf80, resulting in assembly of the trimeric SOSS1 complex at DSBs, which is in close proximity to Y1P RNAPII and R-loop structures, thereby stimulating DDR by promoting condensation of these factors into larger assemblies. See also Figure S18.

Next, we wondered whether the trimeric SOSS1 complex is biologically important for DDR. To test this, we first monitored DNA repair using the comet assay. Depletion of INTS3 or hSSB1 resulted in a substantial delay in DNA repair (Figures 7A and S18A; knockdown of RAD51 was used as a positive control). We subsequently monitored γH2AX clearance at several time points after IR in WT and SOSS1-depleted cells by immunofluorescence (Figure S18B) and observed a significant delay in DNA repair. To identify in which DSB repair pathway the SOSS1 complex might be involved, we used reporter cell lines. Specifically, DR-GFP HR HeLa reporter cells were used to study the HR repair efficiency. The stably integrated DR-GFP cassette has a SceGFP sequence that contains an I-SceI cutting site, followed by a stop codon to avoid the NHEJ and an iGFP sequence used as the inframe repair template. Following transient expression of the pCBASceI plasmid (expressing I-SceI), only cells that undergo HR will generate functional GFP, which can be monitored by fluorescence-activated cell sorting (FACS). In this system, we observed a modest but significant, inhibition of HR in cells depleted of the SOSS1 complex (Figures 7B and S18C; depletion of BRCA1 was used as a positive control). It should be noted that the HR reporter system is limited to only a small number of cells (5%), which can cause a weaker detectable phenotype. Additionally, we also used another HeLa-based reporter system in which the disrupted GFP is re-activated by NHEJ (Figure S18D). In this system, we used the DNA-PK inhibitor wortmannin as a positive control and observed a significant reduction in NHEJ efficiency. The depletion of INTS3 caused only weak inhibition of NHEJ, while depletion of hSSB1 led to increased NHEJ efficiency. Finally, we performed a clonogenic assay and detected a growth defect caused by the trimeric SOSS1 depletion upon IR treatment (Figure 7C).

Collectively, these data show that the trimeric SOSS1 complex plays a role in promoting timely repair of DNA damage, primarily by acting within the HR pathway.

## Discussion

Efficient repair of DSBs via HR requires the trimeric SOSS1 complex.^11^ One of the possible mechanisms by which hSSB1 may be recruited to sites of DNA damage is phosphorylation. Previously, DNA damage-induced ATM and DNA-PK-mediated phosphorylation of hSSB1 on residues T117 and S134, respectively, has implicated hSSB1 in DNA repair.^10,47^ Here, we demonstrate that, upon IR, c-Abl specifically phosphorylates hSSB1 on residues Y102, Y115, and Y74 (Figure 1). The residue Y74 is located within the OB-fold domain, mediating ssDNA binding.^40^ Y102 is located inside the binding interface with INTS3, possibly functioning in trimeric SOSS1 complex assembly. The Y115 residue is present within the unstructured domain (Figures 1E and S1E). Previously, we have reported that c-Abl is present at DSBs,^22^ serving multiple roles in DDR.^21^ However, its role in phosphorylating hSSB1 was unknown. We further demonstrated that the phosphorylation of hSSB1 by cAbl is critical for its presence at DSBs. Additionally, this phosphorylation event is involved in the interaction between hSSB1 and Y1P RNAPII and R-loop structures, which is required for phase separation at DSBs (Figure 6). These findings emphasize the importance of c-Abl and hSSB1 in DDR. We proposed that the phosphorylated hSSB1 works as a signal transducer, initiating the recruitment and/or assembly of the trimeric SOSS1 complex at DSBs. The SOSS1 complex interacts directly with both non-phosphorylated and active RNAPII. Our data suggest that c-Abl initiates DSBs signaling in a dual manner: by phosphorylating hSSB1, which leads to the recruitment of the trimeric SOSS1 complex to DSBs, and by phosphorylating Y1 CTD RNAPII, stimulating transcription at DSBs.^22^

The thorough biochemical characterization of the binding properties of the trimeric SOSS1 complex enabled us to provide a possible explanation for the coexistence of RPA and hSSB1 at DSBs.^8^ Our data suggest that RPA coats the ssDNA portion of the resected ends of DSBs, while the trimeric SOSS1 complex recognizes R-loops and/or RNA: DNA hybrids formed, most likely, behind RNAPII.^22,23^

Several studies have suggested the role of liquid-like condensates of biomolecules in the cellular response to DSBs. In particular, different proteins, such as NONO, RAP80, MRNIP, and RPA, can undergo phase separation at DSBs to recruit and regulate other repair factors. These condensates can modulate various aspects of DSB repair, such as transcription, ubiquitination, and end resection. DSB-associated condensates are influenced by different signaling pathways, such as ATM and DNA-PKcs. LLPS is a versatile mechanism that may explain how dynamic responses to DNA damage are orchestrated.^29,48–50^

Our data show that the purified CTD of RNAPII (phosphorylated or not) and various NA structures (ssDNA, ssRNA, and R-loops) can enter the pre-formed SOSS1 condensates, suggesting that it serves as a scaffold for RNAPII at DSBs (Figures 5 and S15). We also observed *in vivo* condensation of hSSB1 and INTS3 by using an optogenetic tool upon DNA damage (Figure 6). These data uncover a role of the trimeric SOSS1 complex in promoting partition of RNAPII into condensates, which may enable efficient clustering of repair factors at DSBs.

Recent work by Xu et al.^51^ suggested that hSSB1 may not only associate with INTS3 and c9orf80 to form the trimeric SOSS1 complex but also with the entire Integrator complex. This association is required for the recognition of RNAPII-generated R-loops at promoter-proximal sites genome-wide by the combined Integrator-hSSB1-c9orf80 complex, which suppresses transcription-borne genome instability. Moreover, hSSB1 alone promotes formation of condensates via its IDR domain, similarly to the trimeric SOSS1 complex, of the entire Integrator complex. These data suggest that hSSB1 may coexist in two distinct complexes: the trimeric SOSS1 complex, specialized in promoting the repair of DSBs, in addition to the Integrator-hSSB1-c9orf80 complex, specialized in suppressing genome-wide transcription-borne genome instability emanating from aberrant promoter-proximal pausing. Importantly, our work provides direct evidence of a mechanistic explanation for the requirement for SOSS1 complex formation in the recognition of R-loops in the presence of RPA.

Collectively we propose that DNA damage-activated c-Abl phosphorylates hSSB1 protein and RNAPII. p-hSSB1 subsequently associates with INTS3 and c9orf80, leading to the formation of the trimeric SOSS1 complex, which is required for efficient binding to R-loops and RNAPII at DSBs. Consequently, the trimeric SOSS1 complex, together with RNAPII and NAs, promotes formation of condensates with liquid-like properties to boost efficient DDR at DSBs (Figure 7D).

### Limitations of the study

One of the limitations of this study is that we could not test the effect of phase separation on DSB repair using hSSB1 and INTS3 IDR mutants that fail to form condensates. These mutants showed cytoplasmic localization (Figure 6B), which would interfere with their recruitment to DSBs and their function in DNA repair. Therefore, we could not assess whether the formation of hSSB1-INTS3 condensates is essential for DSB repair or whether it is a secondary consequence of the DDR.

Another limitation is that we could not perform rescue experiments with phosphorylation mutants of hSSB1 and INTS3. We found that all phosphorylation mutants were not recruited to DSBs (Figures 1F and 1G), suggesting that phosphorylation is a prerequisite for hSSB1-INTS3 condensate formation and DSB repair. However, we could not rule out the possibility that phosphorylation also affects other aspects of hSSB1 and INTS3 function at the sites of DSBs, such as protein stability, interactions, or localization. Therefore, we could not determine whether phosphorylation directly regulates phase separation or whether it has other roles in DSB repair.

Another possible limitation of the study is that it did not address whether the entire Integrator complex is also involved in DDR or whether it is only the trimeric SOSS1 sub-complex that associates with chromatin and facilitates DNA repair. The trimeric SOSS1 complex and the Integrator complex located at DSBs could be newly formed complexes assembled from individual subunits, or they are pre-bound complexes switching from their original role to a damage-responsive state. This is another relevant question because evidence suggest that the Integrator complex is consistently associated with elongating and paused RNAPII to ensure faithful transcription under non-damage conditions. These unanswered questions require further investigations to elucidate the role of the Integrator and SOSS1 complexes in DDR.

### Star⋆Methods

Detailed methods are provided in the online version of this paper and include the following:

- KEY RESOURCES TABLE
- Resource Availability
  ○ Lead contact
  ○ Materials availability
  ○ Data and code availability

- EXPERIMENTAL MODEL AND STUDY PARTICIPANT DETAILS
  ○ Cell lines
- METHOD DETAILS
  ○ Synthetic RNA/DNA substrates
  ○ Plasmids
  ○ Insect cell work
  ○ Protein purification
  ○ *In vitro* phosphorylation assay
  ○ Identification of residues phosphorylated by cABL^CAT^ by mass spectrometry
  ○ Electrophoretic mobility shift assay (EMSA)
  ○ *In vitro* pull-down experiments
  ○ Micro-scale thermophoresis (MST)
  ○ *In vitro* LLPS assays
  ○ Transfection of siRNA and plasmids
  ○ *In situ* proximity ligation assay (PLA)
  ○ Cell lysis, immunoprecipitation and western blot
  ○ Co-immunoprecipitation (CoIP)
  ○ HR/NHEJ reporter assay with FACS
  ○ Laser microirradiation
  ○ *In vivo* optoDroplets live cell imaging
  ○ Comet assay
  ○ Clonogenic assay
  ○ Immunofluorescence (IF)
- QUANTIFICATION AND STATISTICAL ANALYSIS

### Star⋆Methods

#### Key Resources Table

**Table:** 

REAGENT or RESOURCE	SOURCE	IDENTIFIER
**Antibodies**		
RNA Polymerase II RPB1-8WG16	Biolegend	Cat 664912; PRID: AB_2650945
Anti-RNA polymerase II CTD repeat YSPTSPS antibody	Abcam	Cat ab26721; PRID: AB777726
RNA polymerase II CTD repeat YSPTSPS (phospho S2)	Abcam	Cat ab5095; PRID: AB_304749
RNA polymerase II CTD repeat YSPTSPS (phospho S5)	Abcam	Cat ab5131; PRID: AB_449369
RNA Pol II CTD phospho Tyr1 antibody (mAb)	Active Motif	Cat 61383; PRID: AB_2793613
AbFlex® RNA Pol II CTD phospho Tyr1 antibody (rAb)	Active Motif	Cat 92129: PRID: AB_2793809
phospho-Histone H2A.X (Ser139)	Sigma	Cat 05−636: PRID: AB_2924829
Anti-gamma H2A.X (phospho S139)	Abcam	Cat ab11174: PRID: AB_297813
anti-hSSB1	Bethyl	Cat A301-938A; PRID: AB_1548027
anti-hSSB1	Abcam	Cat ab85752: PRID: AB_1860975
anti-hSSB1	LSBio (Lifespan)	Cat LS-C173584; PRID: AB_3075492
anti-INTS3	Bethyl	Cat A302-051A; PRID: AB_1604271
GFP [PABG1]	Chromotek	Cat PABG1-10; PRID: AB_2749857
GFP	Santa Cruz	Cat sc-9996; PRID:A B_627695
ANTI-DNA-RNA HYBRID, CLONE S9.6	Sigma	Cat MABE1095; PRID: AB_2861387
cABL1	Abcam	Cat ab15130; PRID: AB_301675
cABL	Cell Signaling	Cat 2862S; PRID: AB_2257757
Phospho-*c*-Abl (Tyr245)	Thermo Fisher Scientific	Cat 44250; PRID: AB_2533616
phospho-Tyrosine Monoclonal Antibody (pY20)	Thermo Fisher Scientific	Cat 14500182; PRID: AB_2572884
BRCA1	Santa Cruz	Cat sc-6954; PRID: AB_626761
beta-tubulin	Abcam	Cat ab6046; PRID: AB_2210370
RAD51	Santa Cruz	Cat sc-398587; PRID: AB_2756353
**Bacterial and virus strains**		
NEB® 5-alpha Competent E. coli (High Efficiency)	New England Biology	C2987H
**Chemicals, peptides, and recombinant proteins**		
Imatinib	Stratech Scientific	B2171-APE-10mM
**Critical commercial assays**		
Duolink® *In Situ* Red Starter Kit Mouse/Rabbit	Sigma	DUO92101-1KT
**Experimental models: Cell lines**		
HeLa	ATCC	N/A
DRGFP HeLa	This study	N/A
EJ5 HeLa	This study	N/A
hSSB1-GFP HeLa	This study	N/A
Y102A hSSB1-GFP HeLa	This study	N/A
Y115A hSSB1-GFP HeLa	This study	N/A
Y102A&Y115A hSSB1-GFP HeLa	This study	N/A
pHR-mCh-Cry2WT HeLa	This study	N/A
pHR-FUSN-mCh-Cry2WT HeLa	This study	N/A
pHR-HNRNPA1C-mCh-Cry2WT heLa	This study	N/A
pHR-hSSB1-mCh-Cry2WT HeLa	This study	N/A
pHR-INTS3-mCh-Cry2WT HeLa	This study	N/A
pHR-ΔhSSB1-mCh-Cry2WT	This study	N/A
pHR-ΔINTS3-mCh-Cry2WT	This study	N/A
pHR-Y102A hSSB1-mCh-Cry2WT	This study	N/A
pHR-Y115A hSSB1-mCh-Cry2WT	This study	N/A
pHR-Y102A&Y115A hSSB1-mCh-Cry2WT	This study	N/A
**Oligonucleotides**		
Primers	This paper	Tables S1, S3, and S4
siControl (ON-TARGETplus, Dharmacon SMARTpool)	Dharmacon	D-001810-03-05
siBRCA1(ON-TARGETplus, Dharmacon SMARTpool)	Dharmacon	J-003461-09-0005
sihSSB1(ON-TARGETplus, Dharmacon SMARTpool)	Dharmacon	L-014288-01-0005
siINTS3(ON-TARGETplus, Dharmacon SMARTpool)	Dharmacon	L-018360-01-0005
siRAD51 #1*	IDT	5’ GACUGCCAGGAUAAAGCUU 3’
siRAD51 #2*	IDT	5’ GUGCUGCAGCCUAAUGAGA 3’
*Use both siRAD51 #1 and siRAD51 #2 together to do transient knock down.		
**Recombinant DNA**		
2BT	QB3 MacroLab	29666
2BcT	QB3 MacroLab	37236
H6-mOrange	QB3 MacroLab	29723
H6-mCerulean	QB3 MacroLab	29726
438B	QB3 MacroLab	55219
438C	QB3 MacroLab	55220
pRNH1-GFP	NJP Lab	N/A
pRNH1^D210N^-GFP	NJP Lab	N/A
pRNH1^WKKD^-GFP	NJP Lab	N/A
NABP2	Sino Biological	HG22790-ACG-SIB-1Unit
Y102A NABP2	this study	N/A
Y115A NABP2	this study	N/A
Y102A&Y115ANABP2	this study	N/A
pFRT-TODestRFP_RNAseH1	(Ascano M et al.)^52^	Addgene #65785
pCBASceI	(Richardson C et al.)^53^	Addgene #26477
pHR-mCh-Cry2WT	(Shin Y et al.)^46^	Addgene #101221
pHR-FUSN-mCh-Cry2WT	(Shin Y et al.)^46^	Addgene #101223
pHR-HNRNPA1C-mCh-Cry2WT	(Shin Y et al.)^46^	Addgene #101226
pHR-hSSB1-mCh-Cry2WT	this study	N/A
pHR-INTS3-mCh-Cry2WT	this study	N/A
pHR-ΔhSSB1-mCh-Cry2WT	this study	N/A
pH R-ΔINTS3-mCh-Cry2WT	this study	N/A
pHR-Y102A hSSB1-mCh-Cry2WT	this study	N/A
pHR-Y115A hSSB1-mCh-Cry2WT	this study	N/A
pHR-Y102A,Y115A hSSB1-mCh-Cry2WT	this study	N/A
**Software and algorithms**		
GraphPad Prism 9	GraphPad Software, San Diego, California USA, www.graphpad.com	N/A
Fiji	(Schindelinet et al.)^54^	N/A
CellProfiler	(Carpenter et al.)^55^	N/A
BioRender	https://www.biorender.com/	N/A

#### Resource Availability

##### Lead contact

Further information and requests for resources and reagents should be directed to and will be fulfilled by the Lead Contact, Monika Gullerova (monika.gullerova@path.ox.ac.uk).

##### Materials availability

Reagents generated in this study can be made available on request.

### Experimental Model And Study Participant Details

#### Cell lines

Cells were cultured at 37 °C with 5% CO_2_ in high-glucose DMEM medium (Life Technologies, 31966047) supplemented with 10% (v/v) fetal bovine serum (FBS) (Merck, F9665), 2 mM L-glutamine (Life Technologies, 25030024) and 100 units/ml penicillin-streptomycin solution (Life Technologies, 15140122). Cell morphology was frequently assessed via microscopy, and regular mycoplasma authentication was conducted. HeLa cells were obtained from ATCC. The stable wild-type hSSB1-GFP and 102A/115A/102&115A hSSB1-GFP mutants were generated with Lipofectamine 3000 (Invitrogen, L3000001) transfection followed by 500 μg/mL hygromycin B (Gibco, 10687010) selection for 10 days. Single-cell sorting was performed to ensure monoclonal-based growth in a 96-well plate (supplemented with 1:1 conditioned HeLa media to fresh media). HeLa HR/NHEJ reporter cell lines were generated with linearized DRGFP and EJ5 cassettes via Lipofectamine LTX (Invitrogen, 15338100) transfection followed by 2 μg/mL puromycin selection for 2 weeks before being single-cell sorted. Monoclonals were progressively grown until sufficient confluency. The correct cassette integration was validated by transfecting the I-SceI overexpression plasmid (Addgene, 26477^53^) for 48 h and measuring GFP induction by flow cytometry. The colony with the highest GFP signal was further validated with Western blot by siRNA ablation of the target proteins. To produce stable optoDroplet cell lines expressing Cry2 fusion constructs, lentiviral constructs were transfected with Lipofectamine 3000 (Invitrogen, L3000001) into 293T cells and incubated at 37°C, 5% CO_2_ for 48h. Viral supernatants were collected 48h after transfection and filtered with 0.45 μm syringe filters (Sigma, SLHV033R). HeLa cells seeded at ~70% confluency, were infected by adding 1 mL of filtered viral supernatant directly to the cell medium. Viral medium was replaced with normal growth medium 48 h after infection.

The DNA damage was generated with γ-rays by CS-137 source (Gravatom, RM30/55). Cells were treated with 1μM cAbl inhibitor Imatinib (Stratech Scientific, B2171-APE-10mM) for 1h prior to the induction of DNA damage, and cells were harvested 10 min postirradiation (IR = 10Gy) unless stated differently.

## Method Details

### Synthetic RNA/DNA substrates

Oligonucleotides for preparing synthetic fluorescently-labelled (Cy3) RNA/DNA substrates were purchased from Sigma (HPLC purified) and their sequences are available in key resources table and Table S2. Substrates were prepared by mixing 3 pmol of labeled oligonucleotides with a 3-fold excess of the unlabelled oligonucleotides in the annealing buffer [25 mM Tris-HCl, pH 7.5, 100 mM NaCl, 3 mM MgCl_2_], followed by initial denaturation at 75°C for 5 min. Substrates were then purified from a native PAGE gel.

### Plasmids

Fragment of DNA containing the ORF of hSSB1 was cloned into plasmid 2BT (pET His6 LIC cloning vector, Addgene plasmid #29666) via ligation independent cloning (LIC). Fragments of DNA containing the ORFs of hSSB1, hSSB1^1−139^ (hSSB1^ΔIDR^), INTS3, and c9orf80, respectively, were cloned into plasmid 438B (pFastBac His6 TEV cloning vector with BioBrick Polypromoter LIC subcloning, Addgene plasmid #55219). Constructs 438B-INTS3, 438B-hSSB1, and 438B-c9orf80 were combined using BioBrick Polypromoter LIC subcloning into a single construct enabling co-expression of the three subunits of the trimeric SOSS1 complex from a single virus in insect cells. To fluorescently tag INTS3 and INTS3^1−958^ (INTS3^ΔIDR^), the ORFs were cloned into plasmid H6-mCerulean (pET Biotin His6 TEV mCerulean LIC cloning vector, Addgene plasmid #29726). In the second step, the ORFs for the fused, fluorescent-tagged INTS3s-mCerulean were cloned into 438B vector. Analogously, hSSB1 was fluorescently tagged in two steps by first cloning the ORF into plasmid H6-mOrange (pET Biotin His6 mOrange LIC cloning vector, Addgene plasmid #29723) and then into plasmid 438B.

The plasmids enabling co-expression of the fluorescent-labelled trimeric SOSS complexes were assembled identically, as described above. A full list of generated plasmids is available in key resources table and Table S3. Plasmids 2BT, 2BcT, 438B, 438C, H6-mCerulean, and H6-mOrange were purchased directly from QB3 Macrolab (UC Berkeley).

To generate plasmids enabling expression of the kinase module of TFIIH complex in insect cells, the ORFs for CDK7, MAT1, and CCNH were cloned into plasmid 438B and later combined into a single construct. Plasmid enabling expression of cABL^CAT^ (AA 83− 534), alongside PTP1b^1-238^ was generously provided by Gabriele Fendrich and Michael Becker at the Novartis Institutes for Biomedical Research, Basel. Plasmid expressing catalytically inactive cABL^CAT^ D363A was generated by site-directed mutagenesis. Plasmids 2BcT-GFP-hCTD and 2BcT-mCherry-hCTD (provided by Katerina Linhartova) were used to express and purify the full-length C-terminal domain of the catalytic subunit of RNAPII (hCTD) fused with msfGFP and mCherry, respectively. Plasmid pGEX4T1-(CTD)_26_-(His)_7_ (provided by Olga Jasnovidova) was used to express and purify GST-(CTD)_26_-(His)_7_. All constructs (key resources table) were verified by sequencing.

### Insect cell work

To generate viruses enabling the production of proteins in insect cells, the coding sequences and the necessary regulatory sequences of the constructs were transposed into bacmid using *E. coli* strain DH10bac. The viral particles were obtained by transfection of the bacmids into the *Sf*9 cells using FuGENE Transfection Reagent and further amplification. Proteins were expressed in 300 mL of Hi5 cells (infected at 1×10^6^ cells/ml) with the corresponding P1 virus at a multiplicity of infection >1. The cells were harvested 48 h post-infection, washed with 1x PBS, and stored at −80°C.

### Protein purification

#### Purification of hSSB1

Five grams of *E. coli* BL21 RIPL cells expressing hSSB1 were resuspended in ice-cold lysis buffer [50 mM Tris-HCl, pH 8; 0.5 M NaCl; 10 mM imidazole; 1 mM DTT], containing protease inhibitors (0.66 μg/mL pepstatin, 5 μg/mL benzamidine, 4.75 μg/mL leupeptin, 2 μg/mL aprotinin) at +4°C. Cells were opened up by sonication. The cleared lysate was passed through 2 mL of Ni-NTA beads (Qiagen), equilibrated with buffer [50 mM Tris-HCl, pH 8; 500 mM NaCl; 10 mM imidazole; and 1 mM DTT]. hSSB1 was eluted with an elution buffer [50 mM Tris-HCl, pH 8; 500 mM NaCl; 1 mM DTT, and 400 mM imidazole]. The elution fractions containing hSSB1 were pooled, concentrated, and further fractioned on Superdex S-75 column equilibrated with SEC buffer [25 mM Tris-Cl pH7.5; 200 mM NaCl, 1 mM DTT]. Fractions containing pure hSSB1 were concentrated, glycerol was added to a final concentration of 10% before they were snap-frozen in liquid nitrogen, and stored at 80 °C.

#### Purification of INTS3

Pellets of Hi5 insect cells were resuspended in ice-cold lysis buffer [50 mM Tris pH 8.0; 500 mM NaCl; 0.4% Triton X-100; 10% (v/v) glycerol; 10 mM imidazole; 1 mM DTT; protease inhibitors (0.66 μg/mL pepstatin, 5 μg/mL benzamidine, 4.75 μg/mL leupeptin, 2 μg/mL aprotinin); and 25 U benzonase per mL of lysate]. The resuspended cells were gently shaken for 10 min at 4°C. To aid the lysis, cells were briefly sonicated. The cleared lysate was passed through 2 mL of Ni-NTA beads (Qiagen), equilibrated with buffer [50 mM Tris-HCl, pH 8; 500 mM NaCl; 10 mM imidazole; and 1 mM DTT]. Proteins were eluted with an elution buffer [50 mM Tris-HCl, pH 8; 500 mM NaCl; 1 mM DTT and 400 mM imidazole]. The elution fractions containing proteins were pooled, concentrated, and further fractioned on Superdex S-200 column equilibrated with SEC buffer [25 mM Tris-Cl pH7.5; 200 mM NaCl, 1 mM DTT]. Fractions containing pure INTS3 were concentrated, glycerol was added to a final concentration of 10% before they were snap-frozen in liquid nitrogen, and stored at −80 °C.

#### Purification of the trimeric SOSS1 complex

Pellets of Hi5 insect cells were resuspended in ice-cold lysis buffer [50 mM Tris pH 8.0; 500 mM NaCl; 0.4% Triton X-100; 10% (v/v) glycerol; 10 mM imidazole; 1 mM DTT; protease inhibitors (0.66 μg/mL pepstatin, 5 μg/mL benzamidine, 4.75 μg/mL leupeptin, 2 μg/mL aprotinin); and 25 U benzonase per mL of lysate]. The resuspended cells were gently shaken for 10 min at 4°C. To aid the lysis, cells were briefly sonicated. The cleared lysate was passed through 2 mL of Ni-NTA beads (Qiagen), equilibrated with buffer [50 mM Tris-HCl, pH 8; 500 mM NaCl; 10 mM imidazole; and 1 mM DTT]. Proteins were eluted with an elution buffer [50 mM Tris-HCl, pH 8; 500 mM NaCl; 1 mM DTT and 400 mM imidazole]. The elution fractions containing proteins were pooled, concentrated, and further fractioned on Superose 6 column equilibrated with SEC buffer [25 mM Tris-Cl pH7.5; 200 mM NaCl, 1 mM DTT]. Fractions containing pure complexes were concentrated, glycerol was added to a final concentration of 10% before they were snap-frozen in liquid nitrogen, and stored at −80 °C.

#### Purification of proteins for the in vitro LLPS assays

The trimeric SOSS1 complexes (labeled or not) that were used in *in vitro* LLPS assays were purified as described above, with the following modification: affinity tags were cleaved-off by TEV protease, followed by reverse Ni-NTA affinity chromatography. Additionally, the proteins were frozen in the absence of glycerol.

#### Purification of kinases

cABL^CAT^ w.t. and its catalytic mutant (D363A) mutant were purified as described for hSSB1. The kinase module of the TFIIH complex (CDK7 kinase) was purified as described for the trimeric SOSS1 complex.

#### Purification of CTD polypeptides

GST-(CTD)_26_-(His)_7_ was purified from *E. coli* cells as described for hSSB1. GFP-hCTD and mCherry-hCTD were purified as described for hSSB1, with the following modification: affinity tags were cleaved-off by TEV protease, followed by reverse Ni-NTA affinity chro-matography. Proteins were frozen in the absence of glycerol.

##### Purification of RPA

RPA was purified as described in.^57^

##### In vitro phosphorylation assay

###### Analytical phosphorylation of hSSB1 by cABL^CAT^

hSSB1 and GST (both at 5 μM) were phosphorylated with increasing concentrations of cABL^CAT^ (0.14, 0.26, and 0.58 μM) or cABL^CAT^ D363A (0.58 μM) in buffer K [25 mM Tris-Cl pH7.5, 5 mM MgCl_2_, 2 mM ATP, 1 mM DTT] for 30 min at 37°C (final volume 10 μL). Re-actions were stopped by adding 2xSDS loading dye and boiling at 95 ° C for 5 min. Samples were subsequently analyzed on a 12% SDS-PAGE gel. The presence of modification was detected either by western blotting, followed by immunodetection with pan α-pY antibody or by mass spectrometry (see below).

##### Preparative phosphorylation and purification of CTD polypeptides

Two and half mg of GST-(CTD)_26_-(His)_7_, GFP-hCTD, and mCherry-hCTD were phosphorylated by 350 μg of cABL^CAT^ (to phosphor-ylate Y1 on the CTD) or 250 μg of the kinase module of TFIIH (to phosphorylate S5 and S7 on the CTD) in the presence of 2 mM ATP and 3.5mM MgCl_2_ for 60 min at 30 ° C. Reactions were stopped by placing the reactions at +4 ° C. CTD peptides were purified from the kinases and ATP by size-exclusion chromatography on Superdex S-200, equilibrated with 25 mM Tris-Cl, pH 7.5; 220 mM NaCl, 1 mM DTT.

##### Identification of residues phosphorylated by cABL^CAT^ by mass spectrometry

The procedure was performed as described earlier.^58^ Briefly, protein samples in the gel pieces were alkylated and digested by trypsin. The digested peptides were extracted from gels. One-half of the peptide mixture was directly analyzed, and the rest of the sample was used for phosphopeptide enrichment. Both peptide mixtures were separately analyzed on LC-MS/MS system (RSLCnano connected to Orbitrap Exploris 480; Thermo Fisher Scientific).

MS data were acquired in a data-dependent strategy using survey scan (350−2000 m/z). High-resolution HCD MS/MS spectra were acquired in the Orbitrap analyser. The analysis of the mass spectrometric RAW data files was carried out using the Proteome Discoverer software (Thermo Fisher Scientific; version 1.4) with in-house Mascot (Matrixscience, London, UK; version 2.4.1) search engine utilization. The phosphoRS feature and manual check of the phosphopeptide spectrum was used for the localisation of phosphorylation.

##### Electrophoretic mobility shift assay (EMSA)

Increasing concentrations of the tested proteins (22, 44, 88, 167 nM; for RPA the following concentrations were used: 5, 10, 20, 40 nM) were incubated with fluorescently labeled nucleic acid substrates (final concentration 10 nM) in buffer D [25 mM Tris-HCl, pH 7.5, 1mMDTT, 5mMMgCl_2_ and 100mMNaCl] for 20 min at 37 °C. Loading buffer [60% glycerol in 0.001% Orange-G] was added to the reaction mixtures and the samples were loaded onto a 7.5% (w/v) polyacrylamide native gel in 0.5 × TBE buffer and run at 75 V for 1h at +4 °C. The different nucleic acid species were visualised using an FLA-9000 Starion scanner and quantified in the MultiGauge software (Fujifilm). To calculate the relative amount of bound nucleic acid substrate the background signal from the control sample (without protein) was subtracted using the *band intensity* - *background* option. Nucleic acid-binding affinity graphs were generated with Prism-GraphPad 7.

In the EMSA experiments assessing the effect of RPA on the binding of hSSB1 and the trimeric SOSS1 complex, respectively, the substrate (10 nM) was first pre-coated with 10 or 30 nM RPA, respectively, for 20 min at 37 °C. Subsequently, increasing concentrations (22, 44, 88 nM) of the tested proteins were incorporated and the reaction mixtures were further incubated for 10 min at 37 °C. Reactions were next processed as described above. The statistical significance was determined by unpaired *t* test analysis.

##### *In vitro* pull-down experiments

Purified GST, GST-CTD, GST-Y1P-CTD, and GST-S5,7P-CTD (5 μ g each), respectively, were incubated with the trimeric SOSS1 complex and its variants (5 μg) in 30 μL of buffer T [20 mM Tris−HCl, 200 mM NaCl, 10% glycerol, 1 mM DTT, 0.5 mM EDTA, and 0.01% Nonidet P-40; pH 7.5] for 30 min at 4 ° C in the presence of GSH-beads. After washing the beads twice with 100 μ L of buffer T, the bound proteins were eluted with 30 μL of 4 ×SDS loading dye. The input, supernatant, and eluate, 7 μL each, were analyzed on SDS-PAGE gel.

##### Micro-scale thermophoresis (MST)

Binding affinity comparisons via microscale thermophoresis were performed using the Monolith NT.115 instrument (NanoTemper Technologies). The CTD polypeptides (CTD, Y1P-CTD, and S5,7P CTD, respectively) were fused with msfGFP and served as ligands in the assays. Affinity measurements were performed in the MST buffer [25mMTris−HCl buffer, pH 7.5; 150mMNaCl; 1mMDTT; 5% glycerol; and 0.01% Tween 20]. Samples were soaked into standard capillaries (NanoTemper Technologies). Measurements were performed at 25°C, 50% LED, medium IR-laser power (laser on times were set at 3 s before MST (20 s), and 1 s after), constant concentration of the labeled ligand (20 nM), and increasing concentration of the trimeric SOSS complex (4.8−1200 nM, CTD-GFP and Y1P-CTD-GFP; 28.7−7250 nM, S5,7P CTD). The data were fitted with Hill Slope in GraphPad Prism software.

#### *In vitro* LLPS assays

Condensate formation assays were performed in the buffer H [25 mM HEPES, pH 7.5; 220 mM NaCl; 0.5 mM TCEP] in the presence of a crowding agent (5% PEG-8000). Where indicated, ssDNA, ssRNA, or R-loop substrate was added to a final concentration 2 μM. Upon the addition of the indicated proteins (mCherry-CTD peptides at 0.75 μM; the trimeric SOSS1 complex and its variants at 0,75, 1, 1,5, and 3mM), the mixtures were immediately spotted onto a glass slide, and the condensates were recorded on Zeiss Axio Observer Z1 with a 633 water immersion objective. Analyses and quantifications of the micrographs were performed in Cell-profiler.^59^ First, four micrographs (2048 pixels (px) per 2048 px; 1 px = 0.103 μM) per condition and per experiment were analyzed. Objects (droplets) were identified based on diameter (4−70 px; 0.413−7.5 μM) and intensity using Otsu’s method for thresholding. Picked objects were further filtered based on shape and intensity. For the filtered objects the area and the object count per picture were calculated. The values for droplets were converted from the px to μm based on the metadata of the micrographs. The data were plotted in GraphPad Prism.

The statistical significance of the object counts per picture was determined by unpaired *t* test analysis, while for the area, by a nested *t* test was used.

### Transfection of siRNA and plasmids

RNAi was performed with Lipofectamine RNAiMax (Life technologies, 13778075), delivered at 60nM (except siRAD51, 25nM) final concentration by using reverse transfection with 1 3 10^6^ cells. The used siRNAs are listed in Table S5. Plasmids delivery was achieved with Lipofectamine 3000 or Lipofectamine LTX with the forward transfection. The details of plasmids source and usage are listed in key resources table. For site-directed mutagenesis, pCMV3-hSSB1-GFP plasmid from Sino Biological (HG22790-ACG-SIB-1Unit) was amplified with Q5 Hot Start High-Fidelity DNA Polymerase (NEB, M0493L) with primers listed in Table S4. PCR products were circularized with T4 kinase (NEB, M0201L) and T4 ligase (NEB, M0202S). The parental plasmid was digested with 5U DpnI (NEB, R0176S). Plasmid transformation was achieved by using the heat shock method (42°C, 47s) in DH5a competent cells (NEB, C2987H), then purified with QIAGEN Plasmid *Plus* Midi Kit (QIAGEN, 12943). Mutations were confirmed by sanger sequencing. For constructing optoDroplet plasmids, Gibson assembly method was applied. DNA fragments encoding human hSSB1 and INTS3 were amplified by PCR from NABP2(hSSB1)-GFPspark plasmid (Sino Biological, HG22790-ACG) and INTS3-GFPspark plasmid (Sino Biological, HG15926-ACG) with primers listed in Table S5, then inserted into PHR-mCh-CryWT plasmid (Adgene, 101221^46^) by using NEBuilder HiFi DNA Assembly Cloning Kit (NEB, E5520S). The generated constructs were fully sequenced to confirm the absence of any mutations or stop codons. Control plasmids containing IDRs from FUS or hnRNPA1 were purchased from Adgene (101223, 101226 respectively).

### *In situ* proximity ligation assay (PLA)

Duolink *In Situ* Red Starter Kit Mouse/Rabbit (Merck, DUO92101-1KT) was used to detect protein-protein interactions. Cells were fixed by 4% paraformaldehyde (PFA) in PBS (Alfa Aesar, J61899) for 10min, followed by 10 min permeabilization with 0.1% Triton X-100 (Merck, X100-100ML) before blocking with 100mL blocking buffer from the kit for 1h. The specific primary antibodies (listed in Table S4) were diluted in Duolink dilution buffer and incubated overnight at 4°C. Following primary antibody incubation, PLA probe incubation, ligation and amplification followed the manufacturer’s instructions. Duolink *In Situ* Probemaker PLUS kit (Merck, DUO92009-1KT) was applied to conjugate PLA oligonucleotides (PLUS) to Y1P rat antibody for use in Duolink PLA experiments. For the detection of cAbl and R-loop, the pre-extraction with CSK buffer was performed as described previously.^35^ Image acquisition was performed on Olympus FluoView Spectral FV1200 Laser Scanning Microscope (IX83) with 603 oil immersion objective. The red PLA dots was quantified with CellProfiler^55^ 4.2.1 with sparkle function. Non-parametrical two-tailed Mann-Whitney *u*-test was applied for PLA analysis. Statistical variability was estimated with the standard deviation (SD) and the significance was established at p < 0.05 with Graphpad Prism (Version 9).

### Cell lysis, immunoprecipitation and western blot

Approximately 1 × 10^7^ cells were lysed in 200 μL lysis buffer [50mM Tris pH 8 (Merck, T6066), 150mM NaCl (Merck, S3014), 1mM EDTA (Merck, E9884), 5mM MgCl_2_ (Merck, PHR2486), 0.5% NP40 (Merck, I8896-100ML), 1X protease inhibitors (Merck, 11873580001)/1X phosphatase inhibitors (Thermo Fisher, A32961)(PPI)] for 20 min at 4°C with vortex every 10min. Cytoplasmic supernatant was collected by centrifuge at 500g, 4°C for 5min. The cell chromatin pellet was resuspended with 200 mL lysis buffer and digested with 2mL per sample Pierce Universal Nuclease for Cell Lysis (Thermo Fisher, 88702) and 1mL per sample Benzonase Nuclease (Merck, E1014-25KU) for 30 min at 4°C on wheel with vigorous pipetting every 10min. The soluble nuclear lysate was collected by 10 min centrifuge at 17000g (4°C). 300 μL dilution buffer [50mM Tris pH 8, 150mM NaCl, 1mM EDTA, 5mM MgCl_2_, 1X PPI) was added to the both cytoplasmic and nuclear lysate before take 50 mL Input. GFP-Trap Magnetic Agarose (Proteintech, gtma-20) were washed 3X in cold dilution buffer before adding to the cell lysate for 2h. After pull-down, GFP-Trap beads were wash with high salt washing buffer [50 μM Tris pH 8, 500 μM NaCl, 1 μM EDTA, 5 mM MgCl_2_, 1X PPI] twice and low salt washing buffer [50mM Tris pH 8, 150mM NaCl, 1mM EDTA, 5mM MgCl_2_, 1X PPI] twice before eluted with 1X Laemmli Buffer [62.5 mM Tris pH6.8, 2% sodium dodecyl sulfate (SDS), 2% β-mercaptoethanol, 10% glycerol, 0.005% bromophenol blue] (Alfa Aesar, J61337.AD) and boiled for 10 min at 95°C. NuPAGE 4 to 12% Bis-Tris midi protein gels (Invitrogen, WG1402BOX) and 4−15% Mini-PROTEAN TGX precast protein gels (BioRad, 4561083/4561086) were used for the standard Western blot process. The details of antibodies are listed in Table S1. Blots were imaged with Amersham Hyperfilm ECL (VWR, 28-9068-35). Band intensity was quantified with ImageJ. Statistical analysis was performed with the paired *t* test and ** is refers to p < 0.01, and **** is refers to p < 0.0001.

### Co-immunoprecipitation (CoIP)

When cells reach 50−70% confluency (approximately 7−10 million cells) in 15cm dishes, they were washed three times by ice-cold PBS before scrapped into PBS and collected by centrifugation (500g, 4°C, 5min). The cell pellet was lysed in 5 volumes (200-300μL) of lysis buffer [50mM Tris pH 8, 150mM NaCl, 1mM EDTA, 5mM MgCl_2_, 0.5% NP40, 1X Protease inhibitors and 1X Phosphatase inhibitors (PPI)] plus 2μL per sample Pierce Universal Nuclease for Cell Lysis and 1μL per sample Benzonase Nuclease (Merck, E1014-25KU) for 30 min at 4°C on wheel with vigorous pipetting every 10min. The supernatant was collected with 10 min centrifuge (17000g, 4°C). 1.5X volume (300−450 μL) of dilution buffer [50mM Tris pH 8, 150mM NaCl, 1mM EDTA, 5mM MgCl_2_, 1X PPI] was added to the supernatant. Take 0.1X volume of diluted supernatant for Input. GFP-Trap Magnetic Agarose were washed 33 in cold dilution buffer before adding to the cell lysate. The stably overexpressed GFP-tagged proteins were captured by rotating on 4°C for 1.5h. After pull-down, beads were wash with dilution buffer three times before eluted with 2X Laemmli Buffer [62.5 mM Tris pH6.8, 2% sodium dodecyl sulfate (SDS), 2% β-mercaptoethanol, 10% glycerol, 0.005% bromophenol blue] and boiled for 10 min at 95°C.

### HR/NHEJ reporter assay with FACS

For HR/NHEJ Reporter Assay, HeLa reporter cells stably expressing DRGFP cassette and EJ5 cassette were used. 1×10^6^ cells were reverse transfected with 60nM siRNA in a well of a 6-well plate. After 24h, 1×10^5^ cells were reseeded into a new 6-well plate and cultured for another 24h. 1.5 μg pCBASceI plasmid (I-SceI endonuclease expression vector) (Addgene, 26477) was transfected into cells via Lipofectamine 3000 for a further 48h before cells were harvested to run FACS. As an NHEJ reporter cell line positive control, 1mM Wortmannin (sigma, W3144-250UL, a DNA-PK inhibitor) was added to cell culture media after 6h of reseeding and maintained until harvest.

### Laser microirradiation

2×10^5^ HeLa cells stably expressing wild-type hSSB1-GFP and 102A/115A/102&115A mutants were seeded onto CELLview Culture dish (35mm) (Greiner, 627860. After 16h, 10 μM Hoescht 33342 (Thermo Scientific, H3570) was used to sensitize cells for 10 min before laser damage. To inhibit R-loop, pFRT-TODestRFP_RNAseH1 (Addgene, 65785^52^) plasmid was transfected into stable hSSB1-GFP cells via Lipofectamine 3000 for 16h before pre-sensitization. For plasmid-based laser microirradiation, 1×10^5^ HeLa cells were seeded and transfected with hSSB1-GFP and mutant plasmids for 16h before Hoescht treatment. The laser microirradiation was performed with Nikon SoRa microscope and cells were maintained at 37 °C and 5% CO_2_ during the experimental procedures. Laser tracks were made by a 405 pulsed laser with laser power set to 20% at 80 repetitions. The 488 nm channel was monitored every 4 s tracking the GFP intensity. The images were processed using FIJI software.^54^

The association kinetics of hSSB-GFP at sites of laser micro-irradiation were monitored on the SoRA spinning disc confocal microscope by measuring GFP fluorescence over time in the damaged region using the 488-nm laser. To correct for overall bleaching of the signal due to repetitive imaging, fluorescence intensities were normalized against intensities measured in a non-damaged nucleus in the same field after background subtraction, which was determined by fluorescence intensity in the non-damaged part of the nucleus. Relative fluorescence intensities were plotted as a function of time (t) using Microsoft Excel software. Plotted data are averaged values of a minimum of 15 cells from at least two independent biological experiments. To compare between different experimental conditions, data were normalized against the fluorescence intensity in cells before micro-irradiation.

### *In vivo* optoDroplets live cell imaging

Stably integrated optoDroplets HeLa cell lines (listed in key resources table) were seeded on the 35-mm glass-bottom dish (CELLview Culture dish, Greiner, 627860) and grown overnight in normal growth medium to reach ~50% confluency. All live cell imaging was performed using 603 oil immersion objective (NA 1.4) on an Olympus SoRA spinning disk confocal microscope equipped with a temperature stage at 37°C and CO_2_ chamber. For global activation, cells were imaged by use of two laser wavelengths (488 nm for Cry2 activation/560 nm for mCherry imaging) at 25% of laser power, in 10 s pulses 40 times. For DNA damage induced laser stripping, cells were subjected to incubation with 10 μM Hoechst (Thermo Scientific, H3570) for 30 min prior to imaging. Laser stripe was induced using 405 nm laser, followed by light induction at 488 nm and acquisition at 561 nm.

### Comet assay

5000 cells embedded in 0.5% CometAssay LMAgarose (bio-techne, 4250-050-02) on a Cometslide (bio-techne, 4250-050-03). After the gel solidified on slide, cell lysis was performed by immersing slide in lysis buffer (pH = 10) [2.5M NaCl, 0.1M EDTA, 10mM Tris-Base, 10% DMSO (freshly added before use), 1% Triton X-100 (freshly added before use)] overnight at 4°C. After wash away lysis buffer by ddH_2_O, chromatin unwinding process was carried out by immersing the slides in running buffer (pH = 13) [0.3M NaOH, 1mM EDTA] for 1h at 4°C before running the gel at a constant 300mA for 0.5h. Slides was then immersed in neutralization buffer (pH = 7.5) [0.4M Tris-base] for 5min twice before washed by 70% ethanol for 15 min at room temperature and air dried. For visualisation, 2 mg/mL DAPI (BD Biosciences, 564907) was used to stain slides for 5min before washed away by ddH_2_O for 5min. Slide was imaged by EVOS M7000 microscope with 103 objectives. Quantification (tail moment) was performed by using ImageJ with OpenComet plugin. The significance of differences was determined by using unpaired Welch’s correction.

### Clonogenic assay

1000 cells were seeded into a 12-well plate and incubated overnight before being irradiated with 2 Gy. The cells were then cultured for 10−14 days until colonies formed. Subsequently, colonies were fixed and stained with a mixture of 0.5% crystal violet and 20% methanol for 30min. Images were scanned and quantified by ImageJ with the ColonyArea plugin.

### Immunofluorescence (IF)

RNAi was performed before seeding 2 × 10^5^ of cells onto glass coverslips. Cells was fixed and permeabilized with the same way as PLA protocol. For blocking, coverslips were immersed with 10%FBS in PBS for 2h at room temperature before incubating with primary antibodies at 4°C overnight. The primary antibodies was diluted with blocking buffer with the concen-tration showed in Table S4. PBST was used to wash coverslips 3 times before incubating with secondary antibodies Alexa Fluor 488 (Thermo fisher) or Alexa Fluor 647 (Thermo fisher) diluted in blocking buffer at room temperature in dark for 2h. Coverslips were mounted with Mounting Medium with DAPI (Abcam, ab104139-20mL) and sealed with clear nail polish before visualized with Olympus Fluoview FV1200 confocal microscope with a 60× objective lens. Images were quantified by using CellProfiler 4.2.1 with sparkle function.

### Quantification And Statistical Analysis

Statistical tests were performed in GraphPad Prism 9.3.1 and Excel. All error bars represent mean ± SD, unless stated differently. Each experiment repeats at least 3 times (N = 3). Statistical testing was performed using the Student’s *t*-test, one-way ANOVA (for laser stripping), unpaired Welch’s correction (for comet assay analysis), Mann−Whitney test (non-parametric comparison for PLA foci analysis). Significance is listed as *p ≤ 0.05, **p ≤ 0.01, ***p ≤ 0.001, ****p ≤ 0.0001.

## Supplementary Material

Supplementary Material

## Data Availability

Data reported in this paper can be shared by the lead contact upon request. Mass spectrometry proteomics data have been deposited to the ProteomeXchange Consortium via PRIDE^56^ partner repository with the dataset identifier PXD046523. This paper does not report original code. Any additional information required to re-analyze the data reported in this work paper is available from the lead contact upon request.

## In brief

Double-strand breaks (DSBs) are the most severe type of DNA damage. Long et al. show that hSSB1 is modified and forms a trimeric SOSS1 complex that comes to DSBs in an R-loop-dependent manner. At DSBs, SOSS1 and RNA polymerase II form liquid-like repair compartments. Depletion of the SOSS1 impairs DNA repair.

## Highlights

- Double-strand breaks (DSBs) are the most severe type of DNA damage
- c-Abl phosphorylates hSSB1 (SOSS1), enabling its interaction with Y1P RNAPII at DSBs
- The trimeric SOSS1 complex is present at DSBs in an R-loop-dependent manner
- The SOSS1 complex and RNAPII form dynamic liquid-like repair compartments at DSBs
