# Supplementary material for: The phosphorylated trimeric SOSS1 complex and RNA polymerase II trigger liquid-liquid phase separation at double-strand breaks
Source: Cell Rep. Author manuscript; Available in PMC 2025 Jun 24. (PMC7617797; doi:10.1016/j.celrep.2023.113489)
Supplement: Supplementary Material [file EMS206157-supplement-Supplementary_Material.zip › 1-s2.0-S2211124723015012-mmc1.pdf]

Cell Reports, Volume *42*

Supplemental information

The phosphorylated trimeric SOSS1 complex and RNA polymerase II trigger liquid-liquid phase separation at double-strand breaks

Qilin Long, Marek Sebesta, Katerina Sedova, Vojtech Haluza, Adele Alagia, Zhichao Liu, Richard Stefl, and Monika Gullerova

Figure S1

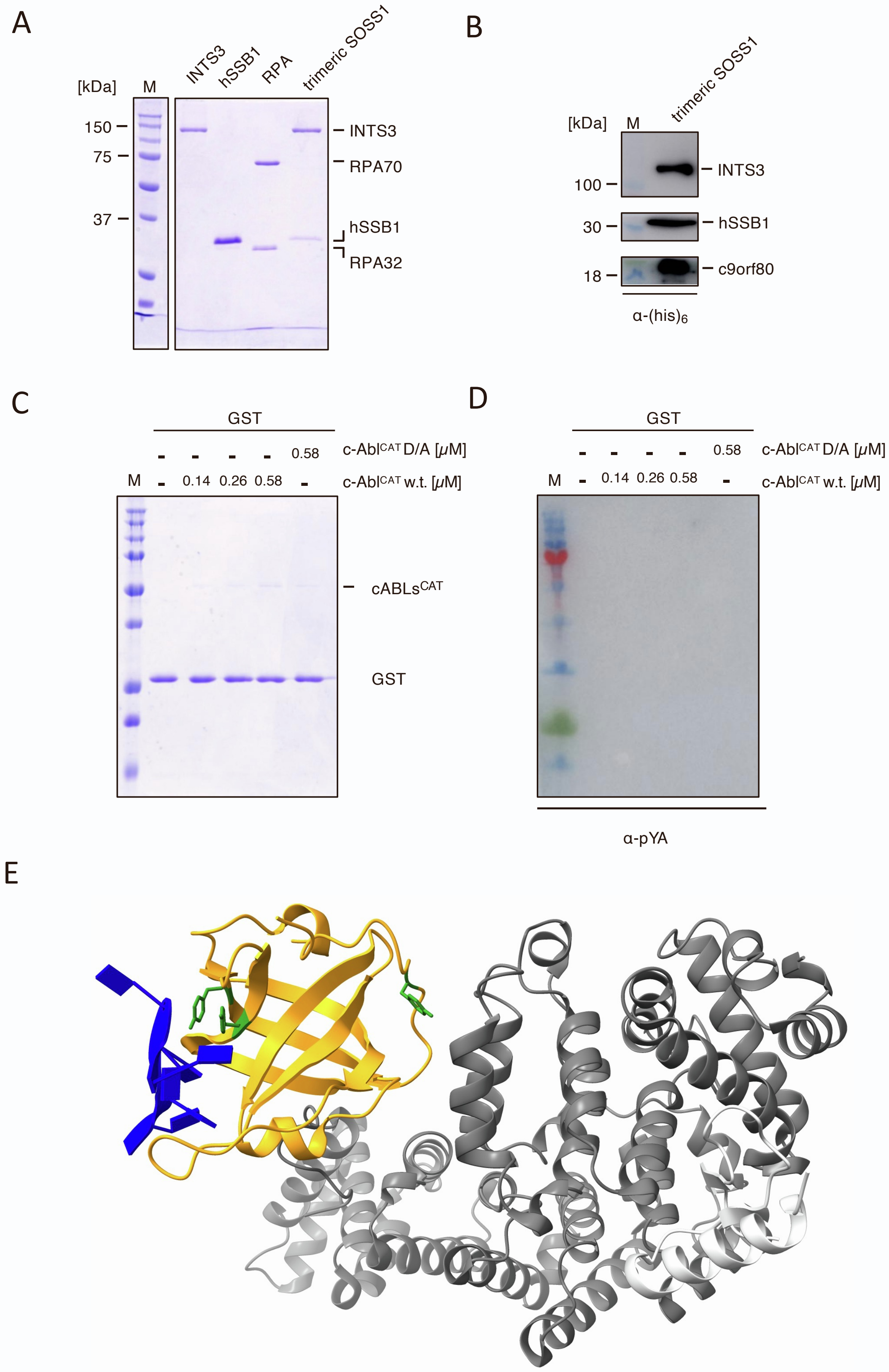
A
[kDa]
M
INTS3
hSSB1
RPA
trimeric SOSS1
150
75
37
INTS3
RPA70
hSSB1
RPA32
B
[kDa]
M
trimeric SOSS1
100
30
18
INTS3
hSSB1
c9orf80
α-(his)6
C
GST
0.58
c-Abl CAT D/A [μM]
M
0.14 0.26 0.58
c-Abl CAT w.t. [μM]
cABLs CAT
GST
D
GST
0.58
c-Abl CAT D/A [μM]
M
0.14 0.26 0.58
c-Abl CAT w.t. [μM]
α-pYA
E

Figure S1. Purification of the subunits of the trimeric SOSS1 complex and determination of the specificity of of cAbl on GST protein *in vitro*. Related to Figure 1.

A. An SDS-PAGE gel depicting purified INTS3, hSSB1, RPA, and the trimeric SOSS1 complex.

B. Western blot detection of the individual subunits of the SOSS1 complex using α-$(his)_6$ antibody.

C. *In vitro* phosphorylation of GST by $cAbl^{CAT}$ as depicted by an SDS-PAGE gel of the reaction.

D. Western blot of samples from (C) detected with α-pY antibody.

E. Depiction of the position of tyrosine residues (in green) of hSSB1 (yellow) on the structural model of the trimeric SOSS1 complex with ssDNA (PDB ID: 4OWW). Dark grey represents INTS3, and light grey represents c9orf80. Residue Y115 is not visible in the structure and could not be highlighted.

Figure S2

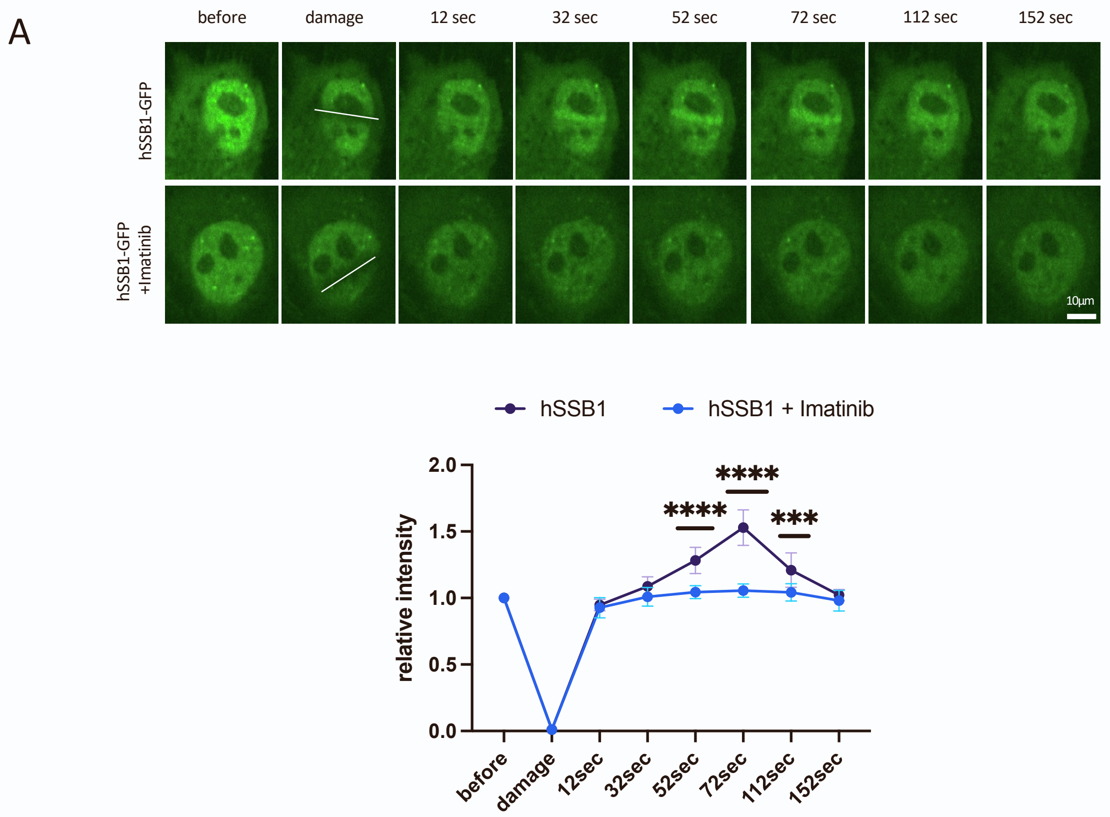
A
before
damage
12 sec
32 sec
52 sec
72 sec
112 sec
152 sec
hSSB1-GFP
hSSB1-GFP +Imatinib
10μm
hSSB1
hSSB1 + Imatinib
relative intensity
2.0
1.5
1.0
0.5
0.0
****
****
***
before
damage
12sec
32sec
52sec
72sec
112sec
152sec

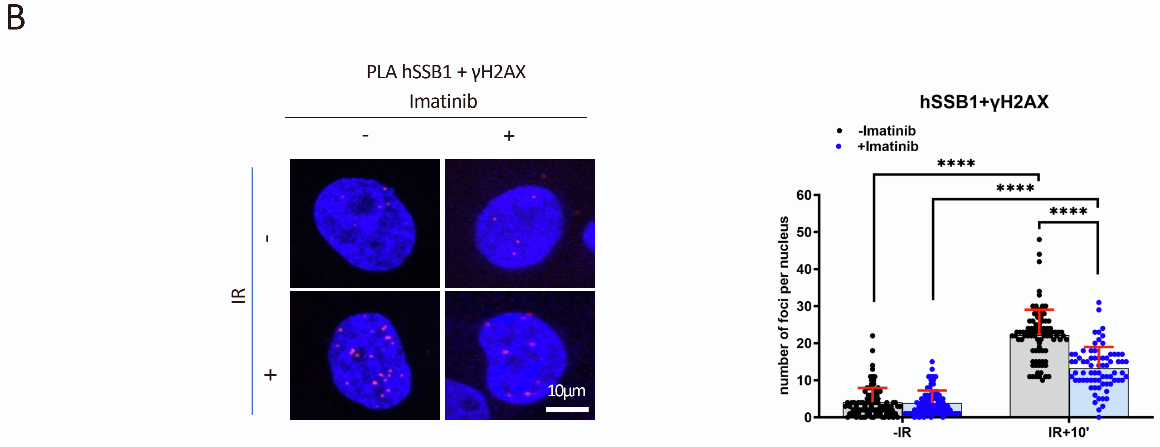
B
PLA hSSB1 + γH2AX
Imatinib
-
+
IR
-
+
10μm
hSSB1+γH2AX
-Imatinib
+Imatinib
number of foci per nucleus
60
50
40
30
20
10
0
****
****
****
-IR
IR+10'

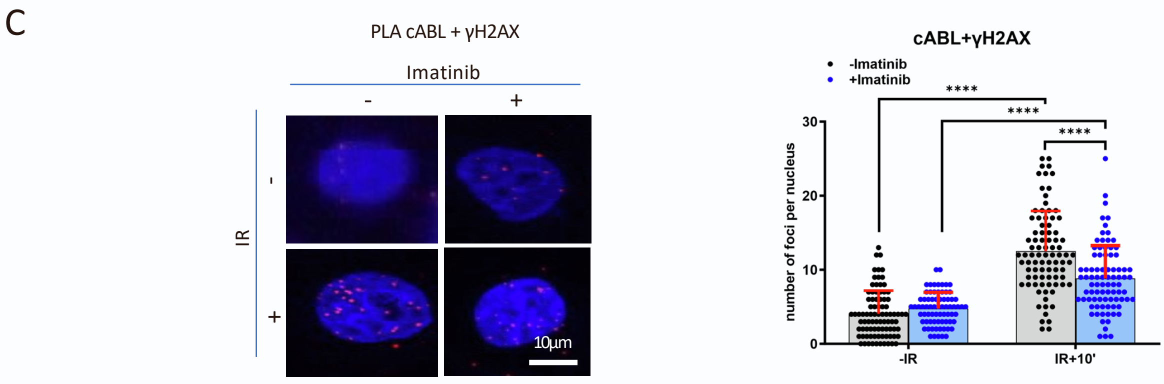
C
PLA cABL + γH2AX
Imatinib
-
+
IR
-
+
10μm
cABL+γH2AX
-Imatinib
+Imatinib
number of foci per nucleus
30
20
10
0
****
****
****
-IR
IR+10'

Figure S2. Inhibition of cAbl reduces the presence of hSSB1 at DSBs. Related to Figure 1.

A. Laser stripping of stably integrated hSSB1-GFP cells with and without Imatinib treatment (1μM, 1h). Representative spinning disk confocal microscopy images (top panel) and quantification ($n \geq 10$) showing GFP signals before and after laser striping at indicated time points; error bar = mean ± SEM; significance was determined using multiple unpaired Student's t-test. ***$p \leq 0.001$ ****$p \leq 0.0001$.

B. PLA of hSSB1 and γH2AX in cells with or without IR and Imatinib treatment (1μM, 1h). IR=10Gy. Left: representative confocal microscopy images; right: quantification of left, error bar = mean ± SD, significance was determined using non-parametric Mann-Whitney test. ****$p \leq 0.0001$.

C. PLA of cAbl and γH2AX in cells with or without IR and Imatinib (1μM, 1h) treatment. IR=10Gy. Left: representative confocal microscopy images; right: quantification of left, error bar = mean ± SD, significance was determined using non-parametric Mann-Whitney test. ####$p \leq 0.0001$

Figure S3

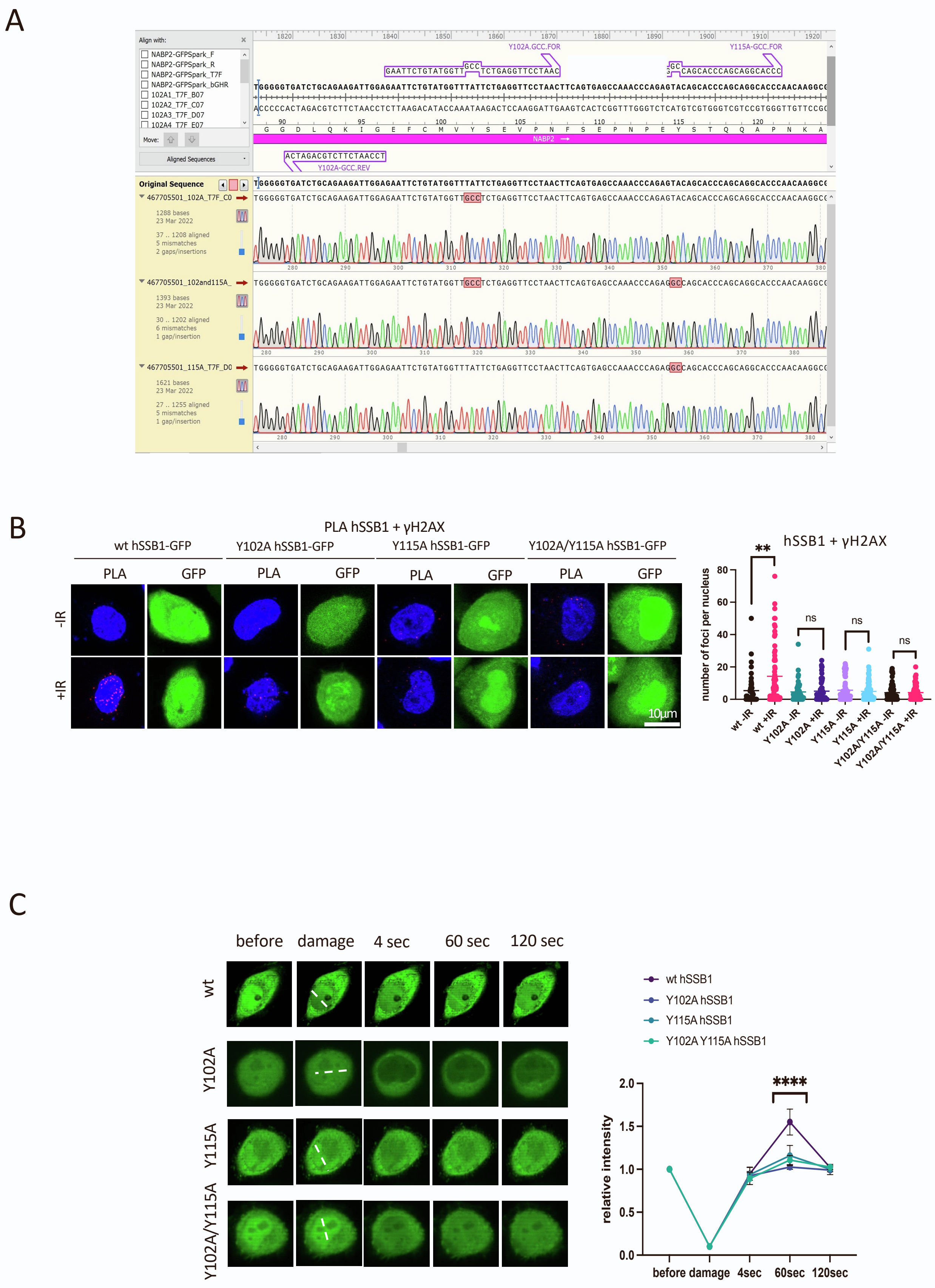
A
Align with:
NABP2-GFPSpark_F
NABP2-GFPSpark_R
NABP2-GFPSpark_T7F
NABP2-GFPSpark_bGHR
102A1_T7F_B07
102A2_T7F_C07
102A3_T7F_D07
102A4_T7F_E07
Move:
Aligned Sequences
Y102A.GCC.FOR
Y115A-GCC.FOR
Y102A-GCC.REV
NABP2
Original Sequence
467705501_102A_T7F_C0
1288 bases
23 Mar 2022
37 .. 1208 aligned
5 mismatches
2 gaps/insertions
467705501_102and115A_
1393 bases
23 Mar 2022
30 .. 1202 aligned
6 mismatches
1 gap/insertion
467705501_115A_T7F_D0
1621 bases
23 Mar 2022
27 .. 1255 aligned
5 mismatches
1 gap/insertion
B
PLA hSSB1 + γH2AX
wt hSSB1-GFP
Y102A hSSB1-GFP
Y115A hSSB1-GFP
Y102A/Y115A hSSB1-GFP
PLA
GFP
-IR
+IR
10μm
hSSB1 + γH2AX
number of foci per nucleus
**
ns
wt -IR
wt +IR
Y102A -IR
Y102A +IR
Y115A -IR
Y115A +IR
Y102A/Y115A -IR
Y102A/Y115A +IR
C
before
damage
4 sec
60 sec
120 sec
wt
Y102A
Y115A
Y102A/Y115A
wt hSSB1
Y102A hSSB1
Y115A hSSB1
Y102A Y115A hSSB1
****
relative intensity
before damage
4sec
60sec
120sec

Figure S3. The phosphorylation of hSSB1 is vital for its recruitment to DSBs. Related to Figure 1.

A. Screenshot of sequencing validation corresponding to hSSB1^{Y102A}-GFP, hSSB1^{Y115A}-GFP and hSSB1$^{Y102A\&Y115A}$-GFP plasmids.

B. PLA of hSSB1 and γH2AX in cells transiently transfected with hSSB1^{wt}-GFP or hSSB1^{Y102A}-GFP, hSSB1^{Y115A}-GFP and hSSB1$^{Y102A\&Y115A}$-GFP plasmids treated with or without IR. IR=2Gy. Left: representative confocal microscopy images; right: quantification of left, error bar = mean ± SD, significance was determined using non-parametric Mann-Whitney test. $^{**}p \leq 0.01$.

C. Laser stripping of stably integrated hSSB1^{wt}-GFP or hSSB1^{Y102A}-GFP, hSSB1^{Y115A}-GFP and hSSB1$^{Y102A\&Y115A}$-GFP cells. Representative spinning disk confocal microscopy images and quantification (n≥10) showing GFP signals before and after laser striping at indicated time points; error bar = mean ± SEM; significance was determined using one-way ANOVA with a multiple comparison test. $^{****}p \leq 0.0001$.

Figure S4

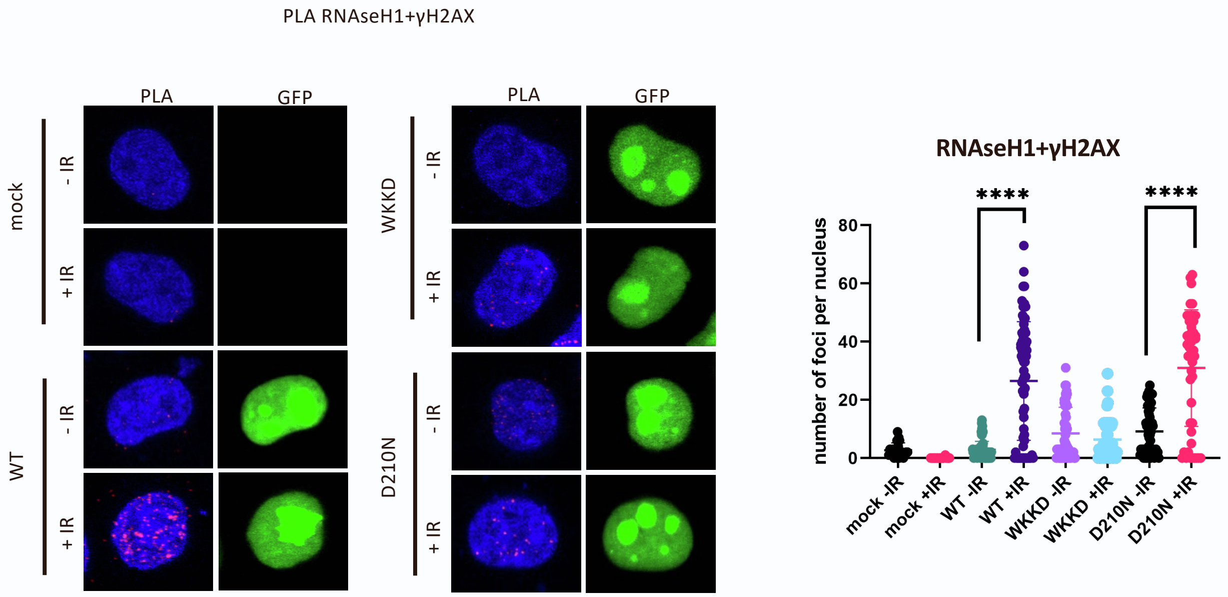
PLA RNAseH1+γH2AX
PLA
GFP
mock
WT
WKKD
D210N
- IR
+ IR
RNAseH1+γH2AX
****
number of foci per nucleus
0
20
40
60
80
mock -IR
mock +IR
WT -IR
WT +IR
WKKD -IR
WKKD +IR
D210N -IR
D210N +IR

Figure S4. RNaseH1 occupancy at DSBs. Related to Figure 2.

PLA of RNAseH1-GFP and γH2AX in cells with transiently transfected $RNAseH1^{wt}$-GFP or $RNAseH1^{WKKD}$-GFP (binding and catalytic) or $RNAseH1^{D210N}$-GFP (catalytic) mutants with or without IR. IR=10Gy. Left: representative confocal microscopy images; right: quantification of left, error bar = mean ± SD, significance was determined using non-parametric Mann-Whitney test. ****$p \leq 0.0001$.

Figure S5

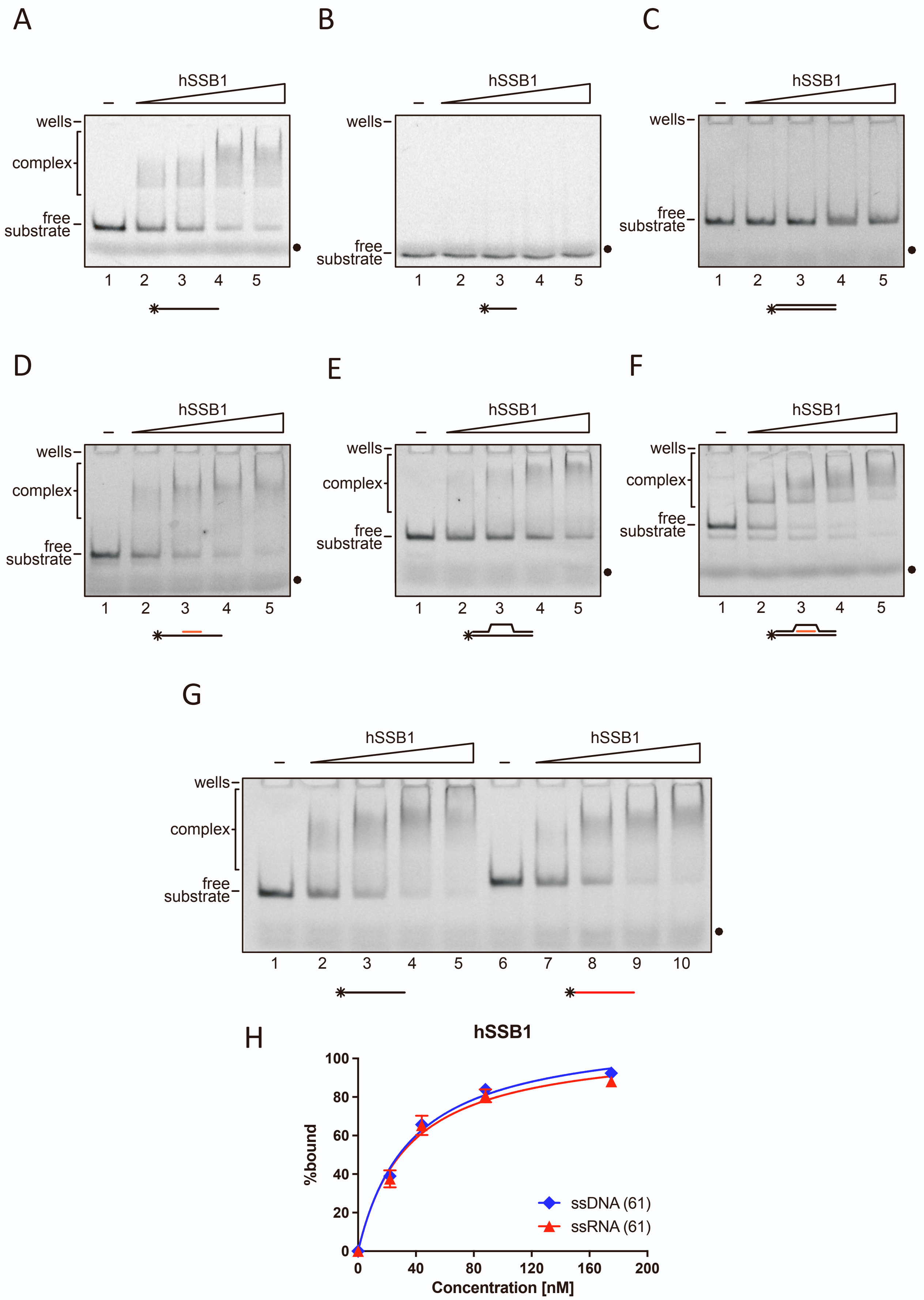
A
B
C
hSSB1
wells
complex
free substrate
1 2 3 4 5
D
E
F
G
1 2 3 4 5 6 7 8 9 10
H
hSSB1
%bound
Concentration [nM]
ssDNA (61)
ssRNA (61)
0
20
40
60
80
100
0
40
80
120
160
200

Figure S5. EMSA experiments of hSSB1 with diverse substrates. Related to Figure 3.

A. Scans of representative EMSA experiments of hSSB1 with 61-mer ssDNA.

B. Scans of representative EMSA experiments of hSSB1 with 21-mer ssDNA.

C. Scans of representative EMSA experiments of hSSB1 with 61-mer dsDNA.

D. Scans of representative EMSA experiments of hSSB1 with RNA:DNA hybrids.

E. Scans of representative EMSA experiments of hSSB1 with DNA bubble.

F. Scans of representative EMSA experiments of hSSB1 with R-loops.

G. Scans of representative EMSA experiments of hSSB1 with 61-mer ssDNA (black) and ssRNA (red).

H. Graph representing quantification of EMSA experiments from G (n=3).

Figure S6

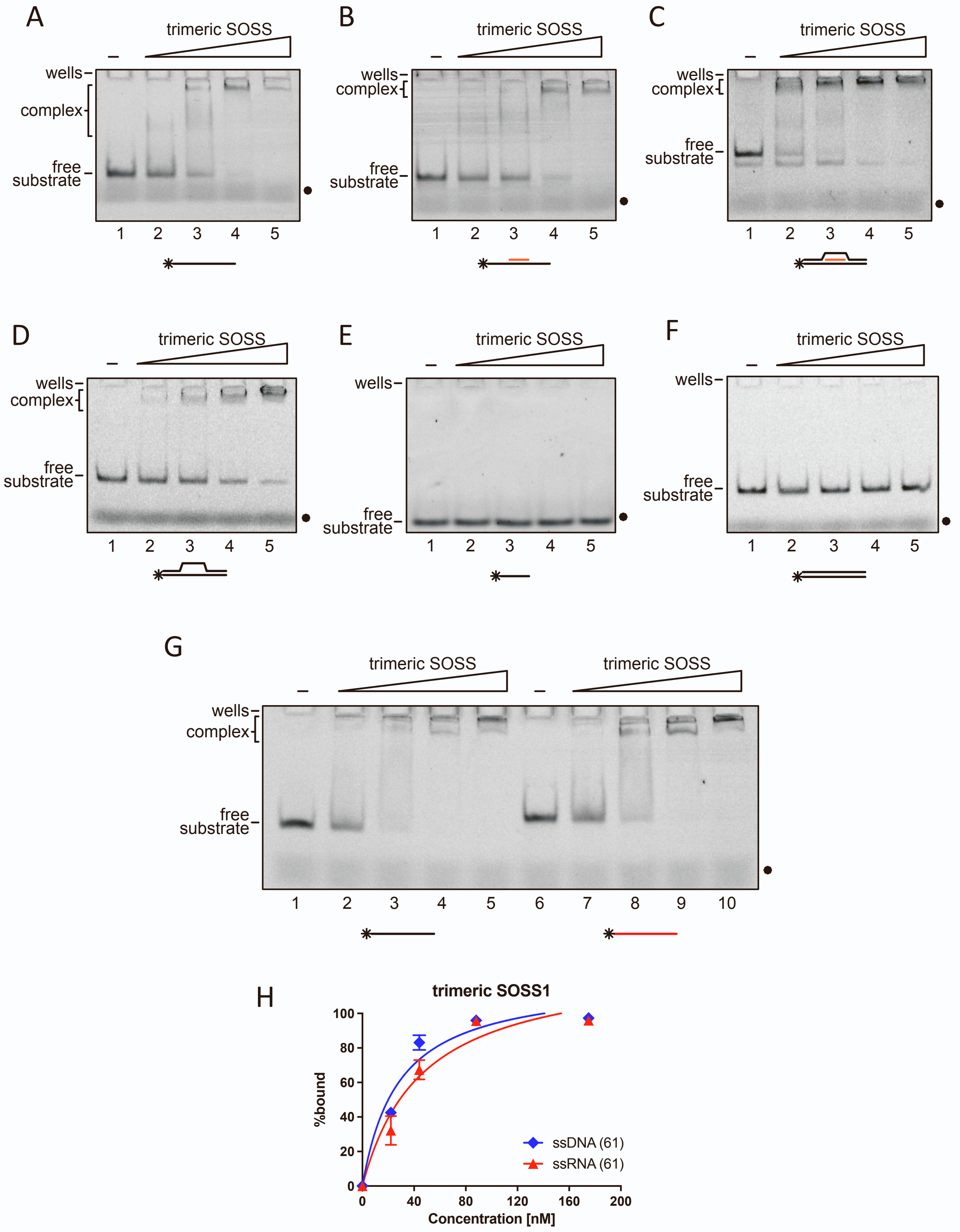
A
trimeric SOSS
wells
complex
free substrate
1 2 3 4 5
B
trimeric SOSS
wells
complex
free substrate
1 2 3 4 5
C
trimeric SOSS
wells
complex
free substrate
1 2 3 4 5
D
trimeric SOSS
wells
complex
free substrate
1 2 3 4 5
E
trimeric SOSS
wells
free substrate
1 2 3 4 5
F
trimeric SOSS
wells
free substrate
1 2 3 4 5
G
trimeric SOSS
trimeric SOSS
wells
complex
free substrate
1 2 3 4 5 6 7 8 9 10
H
trimeric SOSS1
%bound
Concentration [nM]
ssDNA (61)
ssRNA (61)
0 20 40 60 80 100
0 40 80 120 160 200

Figure S6. EMSA experiments of trimeric SOSS1 complex with diverse substrates. Related to Figure 3.

A. Scans of representative EMSA experiments of trimeric SOSS1 with 61-mer ssDNA.

B. Scans of representative EMSA experiments of trimeric SOSS1 with RNA:DNA hybrids.

C. Scans of representative EMSA experiments of trimeric SOSS1 with R-loops.

D. Scans of representative EMSA experiments of trimeric SOSS1 with DNA bubble.

E. Scans of representative EMSA experiments of trimeric SOSS1 with 21-mer ssDNA.

F. Scans of representative EMSA experiments of trimeric SOSS1 with 61-mer dsDNA.

G. Scans of representative EMSA experiments of trimeric SOSS1 with 61-mer ssDNA (black) and ssRNA (red).

H. Graph representing quantification of EMSA experiments from G (n=3).

Figure S7

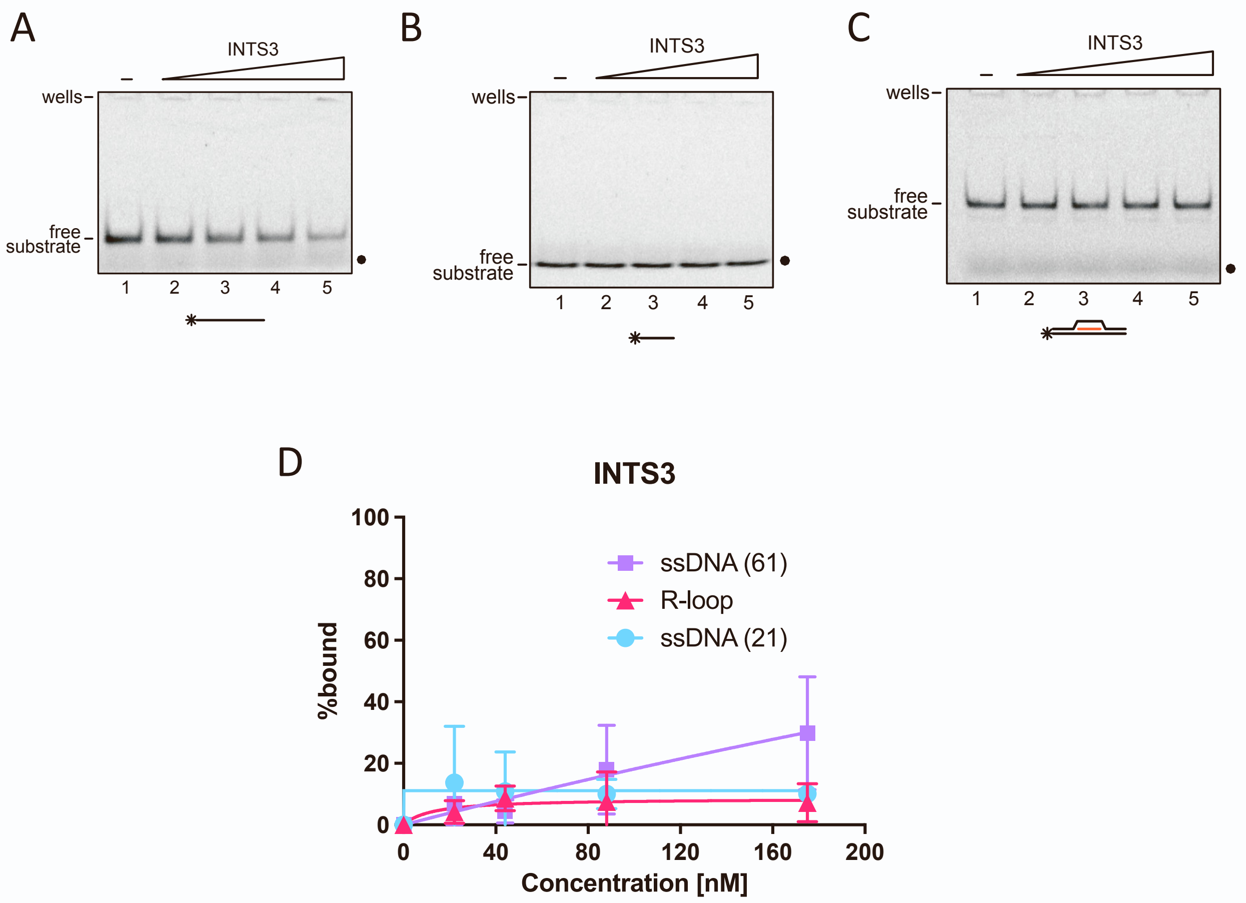
A
B
C
INTS3
wells
free substrate
1
2
3
4
5
D
INTS3
100
80
60
40
20
0
%bound
ssDNA (61)
R-loop
ssDNA (21)
0
40
80
120
160
200
Concentration [nM]

Figure S7. EMSA experiments of INTS3 with diverse substrates. Related to Figure 3.

A. Scans of representative EMSA experiments and conducted between INTS3 with 61-mer ssDNA.

B. Scans of representative EMSA experiments and conducted between INTS3 with 21-mer ssDNA.

C. Scans of representative EMSA experiments and conducted between INTS3 with R-loop.

D. Graph representing quantification of EMSA experiments from A-C (n=3).

Figure S8

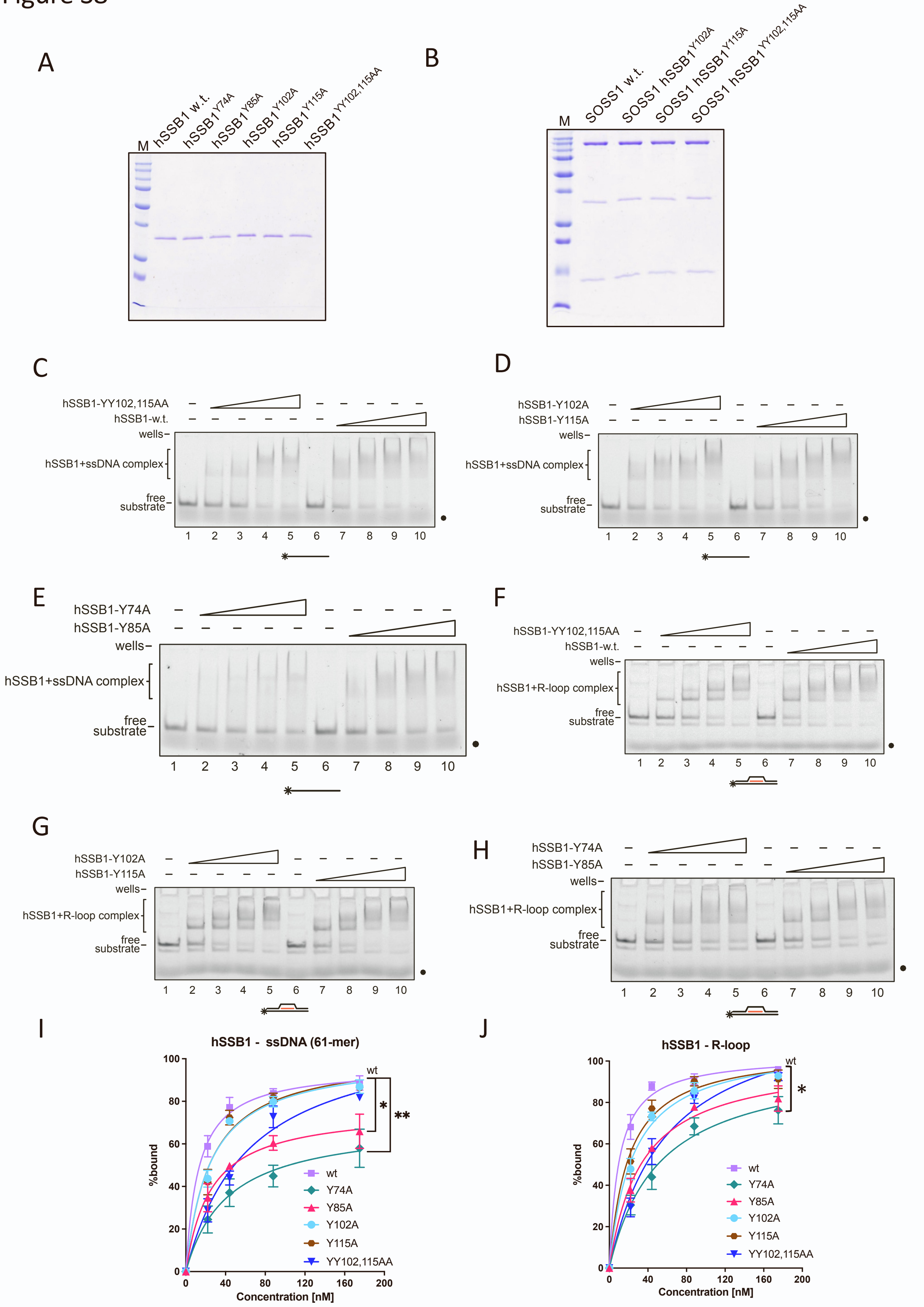

A
M
hSSB1 w.t.
hSSB1 Y74A
hSSB1 Y85A
hSSB1 Y102A
hSSB1 Y115A
hSSB1 YY102,115AA
B
M
SOSS1 w.t.
SOSS1 hSSB1 Y102A
SOSS1 hSSB1 Y115A
SOSS1 hSSB1 YY102,115AA
C
hSSB1-YY102,115AA
hSSB1-w.t.
wells
hSSB1+ssDNA complex
free substrate
1 2 3 4 5 6 7 8 9 10
D
hSSB1-Y102A
hSSB1-Y115A
wells
hSSB1+ssDNA complex
free substrate
1 2 3 4 5 6 7 8 9 10
E
hSSB1-Y74A
hSSB1-Y85A
wells
hSSB1+ssDNA complex
free substrate
1 2 3 4 5 6 7 8 9 10
F
hSSB1-YY102,115AA
hSSB1-w.t.
wells
hSSB1+R-loop complex
free substrate
1 2 3 4 5 6 7 8 9 10
G
hSSB1-Y102A
hSSB1-Y115A
wells
hSSB1+R-loop complex
free substrate
1 2 3 4 5 6 7 8 9 10
H
hSSB1-Y74A
hSSB1-Y85A
wells
hSSB1+R-loop complex
free substrate
1 2 3 4 5 6 7 8 9 10
I
hSSB1 - ssDNA (61-mer)
%bound
Concentration [nM]
wt
Y74A
Y85A
Y102A
Y115A
YY102,115AA
*
**
J
hSSB1 - R-loop
%bound
Concentration [nM]
wt
Y74A
Y85A
Y102A
Y115A
YY102,115AA
*

Figure S8. ***In vitro*** **pull-down of trimeric SOSS1 complexes containing hSSB1 mutants and representative EMSAs of hSSB1 mutants. Related to Figure 3.**

A. An SDS-PAGE gel depicting purified hSSB1 wt, Y102A, Y115A, or Y102A&Y115A (YY102,115AA) mutants, respectively.

B. An SDS-PAGE gel depicting purified trimeric SOSS1 complexes containing hSSB1 wt, Y102A, Y115A, or Y102A&Y115A (YY102,115AA) mutants, respectively.

C. Scans of representative EMSA experiments of hSSB1- Y102A&Y115A (YY102,115AA) mutant and hSSB1wt with 61-mer ssDNA.

D. Scans of representative EMSA experiments of hSSB1-Y102A and Y115A mutants with 61-mer ssDNA.

E. Scans of representative EMSA experiments of hSSB1- Y74A and Y85A (YY102,115AA) mutant and hSSB1wt with 61-mer ssDNA.

F. Scans of representative EMSA experiments of hSSB1- Y102A&Y115A (YY102,115AA) mutant and hSSB1 wt with R-loop.

G. Scans of representative EMSA experiments of hSSB1-Y102A and Y115A mutants with R-loop.

H. Scans of representative EMSA experiments of hSSB1- Y74A and Y85A (YY102,115AA) mutant and hSSB1wt with R-loop.

I. Graph representing quantification of EMSA experiments (n=3) conducted between hSSB1 wt, Y74A, Y85A, Y102A, Y115A, and Y102A&Y115A (YY102,115AA) mutants (at indicated concentrations) and ssDNA (61-mer). Significance was determined using unpaired *t*-test. $**p \leq 0.01$ and $*p \leq 0.05$ represent the comparison between hSSB1 wt and Y74A, Y85A, respectively. Representative gels from the experiments are shown in D-G.

J. Graph representing quantification of EMSA experiments (n=3) conducted between hSSB1 wt, Y74A, Y85A, Y102A, Y115A, and Y102A&Y115A (YY102,115AA) mutants (at indicated concentrations) and R-loop. Significance was determined using unpaired *t*-test. $**p \leq 0.01$ and $*p \leq 0.05$ represent the comparison between hSSB1 wt and Y74A, Y85A, respectively. Representative gels from the experiments are shown in D-G.

Figure S9

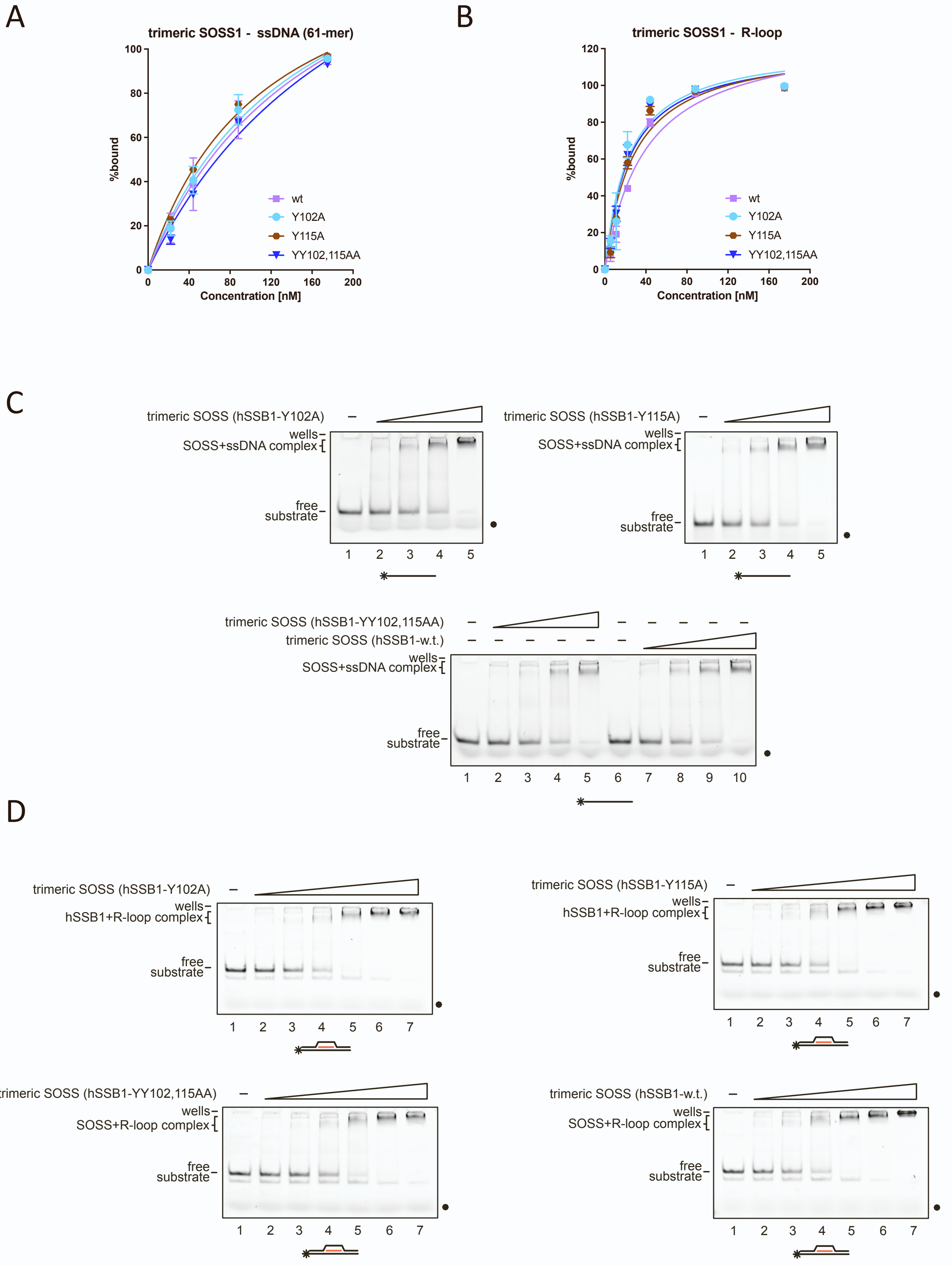
A
trimeric SOSS1 - ssDNA (61-mer)
%bound
Concentration [nM]
wt
Y102A
Y115A
YY102,115AA
B
trimeric SOSS1 - R-loop
%bound
Concentration [nM]
wt
Y102A
Y115A
YY102,115AA
C
trimeric SOSS (hSSB1-Y102A)
trimeric SOSS (hSSB1-Y115A)
wells
SOSS+ssDNA complex
free substrate
1 2 3 4 5
trimeric SOSS (hSSB1-YY102,115AA)
trimeric SOSS (hSSB1-w.t.)
1 2 3 4 5 6 7 8 9 10
D
trimeric SOSS (hSSB1-Y102A)
trimeric SOSS (hSSB1-Y115A)
wells
hSSB1+R-loop complex
free substrate
1 2 3 4 5 6 7
trimeric SOSS (hSSB1-YY102,115AA)
trimeric SOSS (hSSB1-w.t.)
SOSS+R-loop complex

Figure S9. Representative EMSAs of trimeric SOSS1 complexes containing hSSB1 mutants and 61-mer ssDNA or R-loop. Related to Figure 3.

A. Graph representing quantification of EMSA experiments (n=3) of the trimeric SOSS1 containing hSSB1 wt, Y102A, Y115A, or Y102A&Y115A (YY102,115AA) mutants with 61-mer ssDNA (n=3).

B. Graph representing quantification of EMSA experiments (n=3) of the trimeric SOSS1 containing hSSB1 wt, Y102A, Y115A, or Y102A&Y115A (YY102,115AA) mutants with R-loop.

C. Scans of representative EMSA experiments of trimeric SOSS1 containing hSSB1 wt, Y102A, Y115A, or Y102A&Y115A (YY102,115AA) mutants with 61-mer ssDNA.

D. Scans of representative EMSA experiments of trimeric SOSS1 containing hSSB1 wt, Y102A, Y115A, or Y102A&Y115A (YY102,115AA) mutants with R-loop.

Figure S10

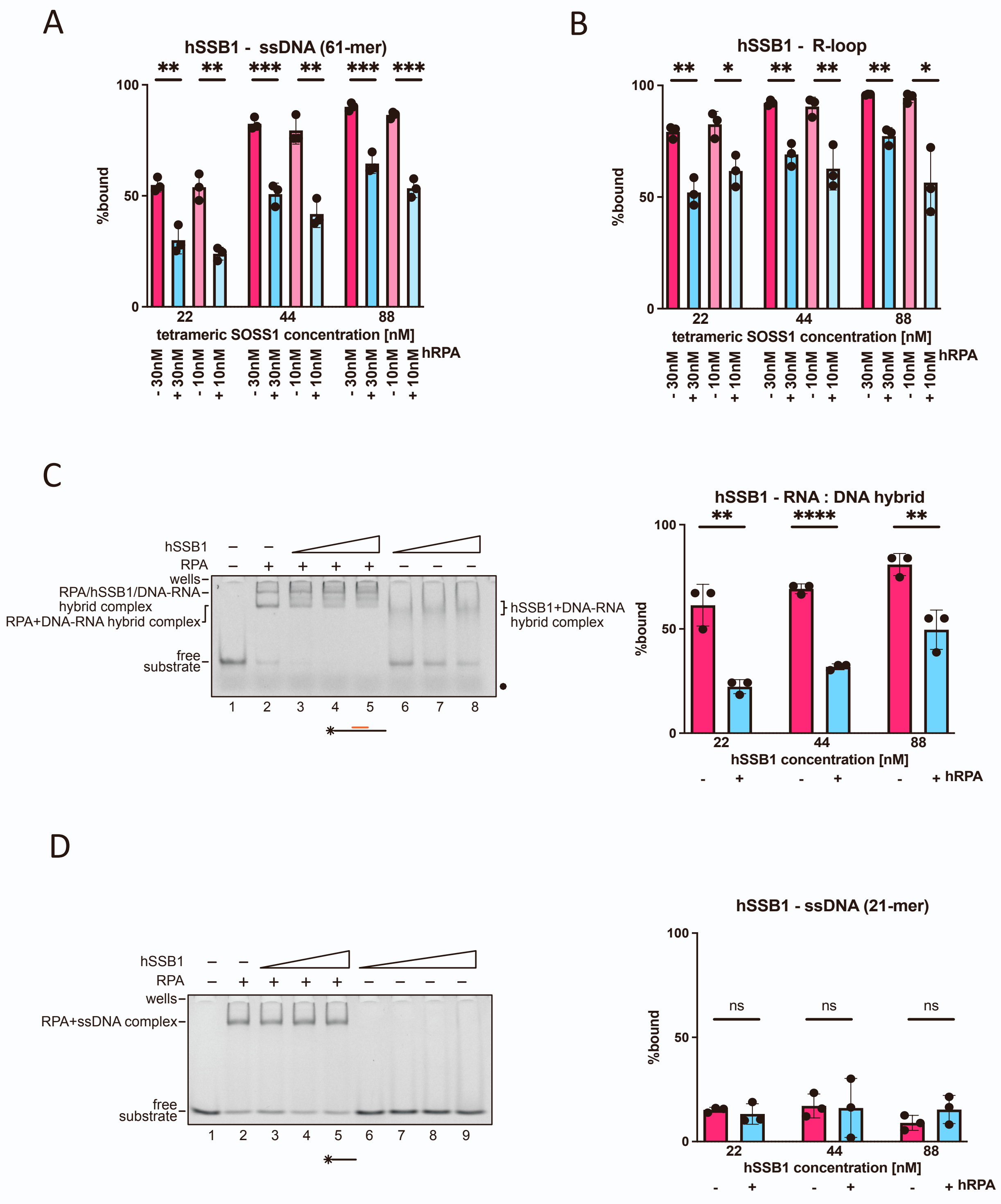

A
hSSB1 - ssDNA (61-mer)
B
hSSB1 - R-loop
%bound
tetrameric SOSS1 concentration [nM]
hRPA
C
hSSB1
RPA
wells
RPA/hSSB1/DNA-RNA hybrid complex
RPA+DNA-RNA hybrid complex
free substrate
hSSB1+DNA-RNA hybrid complex
hSSB1 - RNA : DNA hybrid
hSSB1 concentration [nM]
D
RPA+ssDNA complex
hSSB1 - ssDNA (21-mer)
ns

Figure S10. Competitive EMSA experiments of hSSB1 with diverse substrates. Related to Figure 3.

A. Graph representing quantification of EMSA experiments of hSSB1 with 61-mer ssDNA in absence or presence of RPA at various concentrations (n=3). Significance was determined using unpaired *t*-test. **$p \leq 0.01$ and ***$p \leq 0.001$.

B. Graph representing quantification of EMSA experiments of hSSB1 with R-loop in absence or presence of RPA at various concentrations (n=3). Significance was determined using unpaired *t*-test. *$p \leq 0.05$ and **$p \leq 0.01$.

C. Scans of representative EMSA experiments of hSSB1 with RNA:DNA hybrids in absence or presence of RPA at various concentrations (left) and graph representing quantification of EMSA experiments (n=3) (right). Significance was determined using unpaired *t*-test. **$p \leq 0.01$ ****$p \leq 0.0001$.

D. Scans of representative EMSA experiments of hSSB1 with 21-mer ssDNA in absence or presence of RPA at various concentrations (left) and graph representing quantification of EMSA experiments (n=3) (right).

Figure S11

A

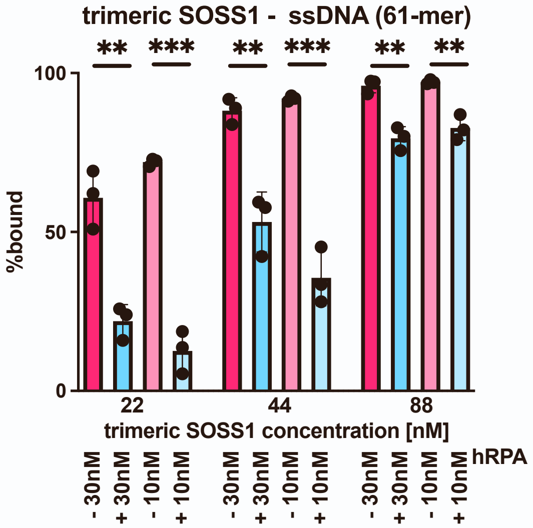

B

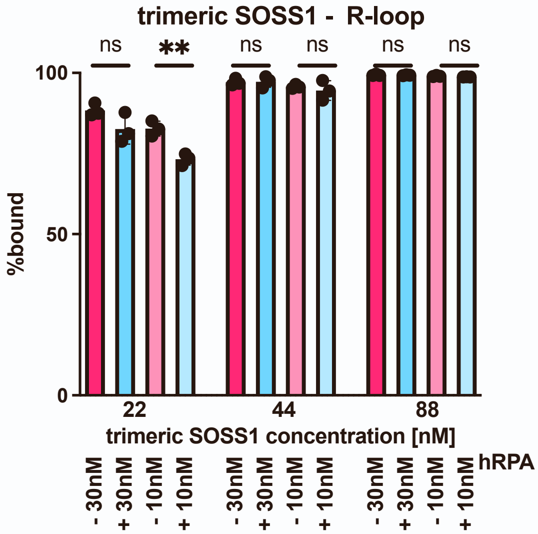

C

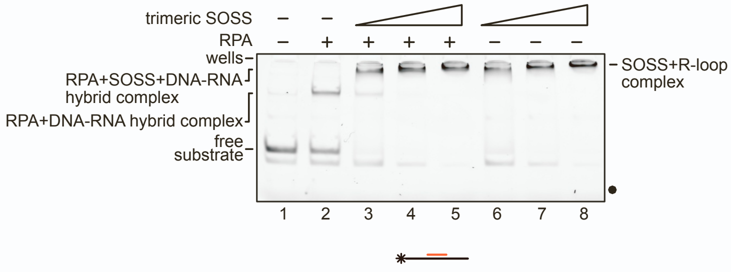

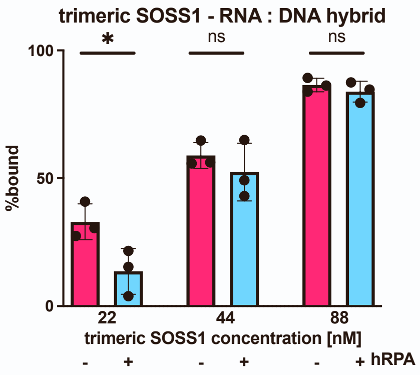

D

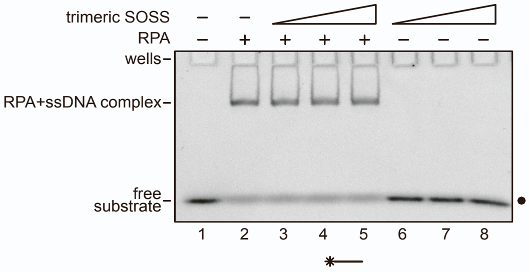

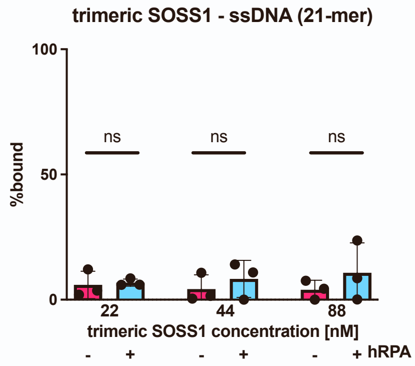

Figure S11. Competitive EMSA experiments of trimeric SOSS1 with diverse substrates. Related to Figure 3.

A. Graph representing quantification of EMSA experiments of trimeric SOSS1 with 61-mer ssDNA in absence or presence of RPA at various concentrations (n=3). Significance was determined using unpaired *t*-test. $^{**}p \leq 0.01$ and $^{***}p \leq 0.001$.

B. Graph representing quantification of EMSA experiments of trimeric SOSS1 with R-loop in absence or presence of RPA at various concentrations (n=3). Significance was determined using unpaired *t*-test. $^{**}p \leq 0.01$.

C. Scans of representative EMSA experiments of trimeric SOSS1 with RNA:DNA hybrids in absence or presence of RPA at various concentrations (left) and graph representing quantification of EMSA experiments (n=3) (right). Significance was determined using unpaired *t*-test. $^{*}p \leq 0.05$.

D. Scans of representative EMSA experiments of trimeric SOSS1 with 21-mer ssDNA in absence or presence of RPA at various concentrations (left) and graph representing quantification of EMSA experiments (n=3) (right).

Figure S12

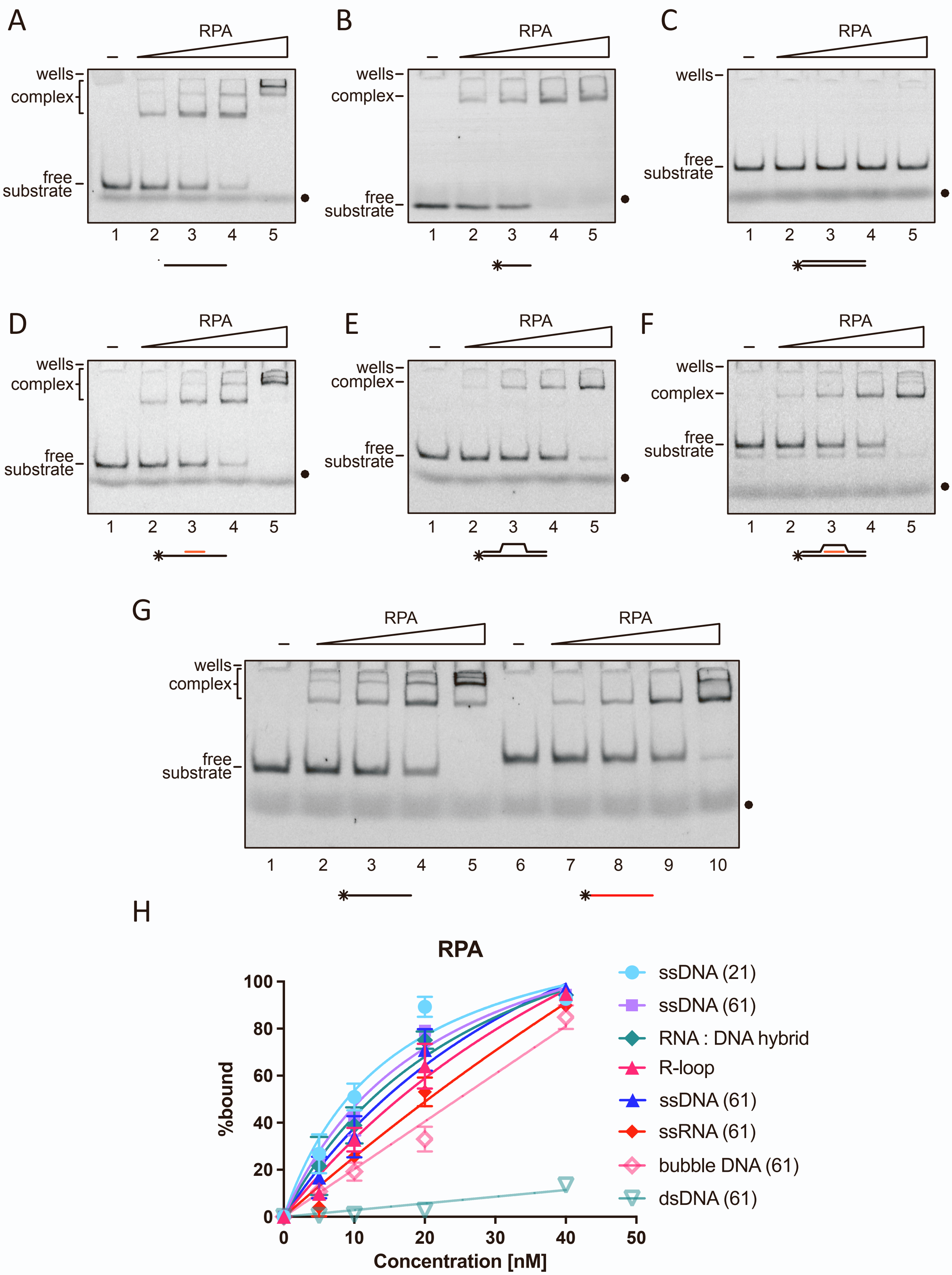

A
B
C
D
E
F
G
H
RPA
wells
complex
free substrate
1
2
3
4
5
6
7
8
9
10
RPA
%bound
Concentration [nM]
ssDNA (21)
ssDNA (61)
RNA : DNA hybrid
R-loop
ssDNA (61)
ssRNA (61)
bubble DNA (61)
dsDNA (61)

Figure S12. EMSA experiments of RPA with diverse substrates. Related to Figure 3.

A. Scans of representative EMSA experiments of RPA with 61-mer ssDNA.

B. Scans of representative EMSA experiments of RPA with 21-mer ssDNA.

C. Scans of representative EMSA experiments of RPA with 61-mer dsDNA.

D. Scans of representative EMSA experiments of RPA with RNA:DNA hybrids.

E. Scans of representative EMSA experiments of RPA with DNA bubble.

F. Scans of representative EMSA experiments of RPA with R-loops.

G. Scans of representative EMSA experiments of RPA with 61-mer ssDNA (black) and ssRNA (red).

H. Graph representing quantification of EMSA experiments from A-G (n=3).

Figure S13

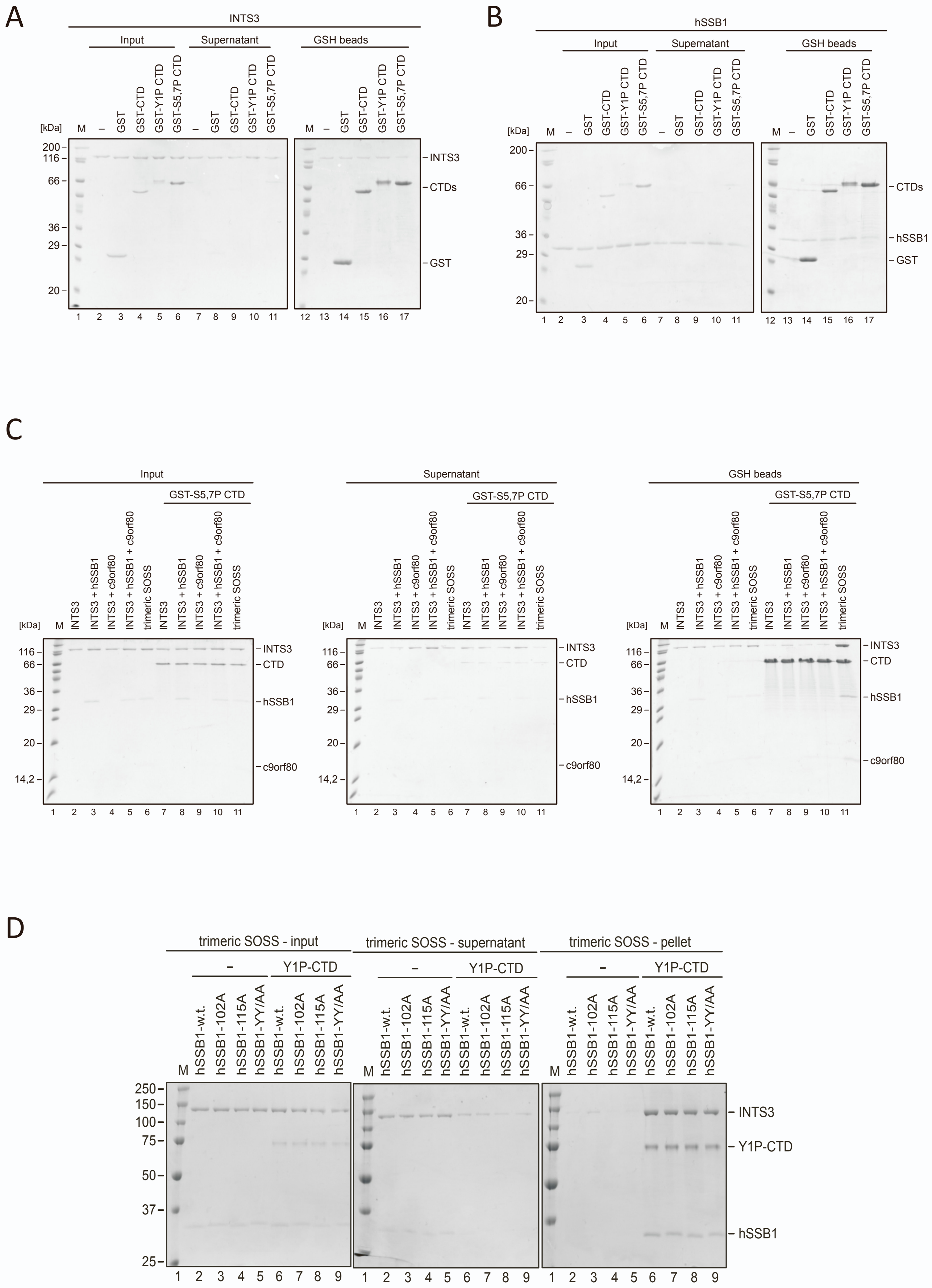
A
INTS3
Input
Supernatant
GSH beads
M
–
GST
GST-CTD
GST-Y1P CTD
GST-S5,7P CTD
[kDa]
200
116
66
36
29
20
INTS3
CTDs
GST
1 2 3 4 5 6 7 8 9 10 11 12 13 14 15 16 17
B
hSSB1
Input
Supernatant
GSH beads
[kDa]
200
66
36
29
20
CTDs
hSSB1
GST
1 2 3 4 5 6 7 8 9 10 11 12 13 14 15 16 17
C
Input
Supernatant
GSH beads
GST-S5,7P CTD
INTS3
INTS3 + hSSB1
INTS3 + c9orf80
INTS3 + hSSB1 + c9orf80
trimeric SOSS
[kDa]
116
66
36
29
20
14,2
INTS3
CTD
hSSB1
c9orf80
1 2 3 4 5 6 7 8 9 10 11
D
trimeric SOSS - input
trimeric SOSS - supernatant
trimeric SOSS - pellet
–
Y1P-CTD
hSSB1-w.t.
hSSB1-102A
hSSB1-115A
hSSB1-YY/AA
M
250
150
100
75
50
37
25
INTS3
Y1P-CTD
hSSB1
1 2 3 4 5 6 7 8 9

Figure S13. *In Vitro* pull-down assay of subunit of SOSS1 complex and purified CTD. Related to Figure 4.

A. A representative SDS-PAGE gel depicting *in vitro* pull-down assay of INTS3 with GST-tagged CTD, GST-tagged CTD modified on tyrosine 1 (Y1P), or GST-tagged CTD modified on serine 5 and 7 (S5,7P).

B. A representative SDS-PAGE gel depicting *in vitro* pull-down assay of hSSB1 with GST-tagged CTD, GST-tagged CTD modified on tyrosine 1 (Y1P), or GST-tagged CTD modified on serine 5 and 7 (S5,7P).

C. A representative SDS-PAGE gel depicting *in vitro* pull-down assay of the individual subunits of the SOSS1 complex alone and in combination with GST-tagged CTD, GST-tagged CTD modified on tyrosine 1 (Y1P), or GST-tagged CTD modified on serine 5 and 7 (S5,7P). Purified trimeric SOSS1 complex was used as a positive control.

D. A representative SDS-PAGE gel depicting *in vitro* pull-down assay of purified trimeric SOSS1 complexes containing hSSB1 wt, Y102A, Y115A, or Y102A&Y115A (YY/AA) mutants with GST-tagged CTD modified on tyrosine 1 (Y1P).

Figure S14

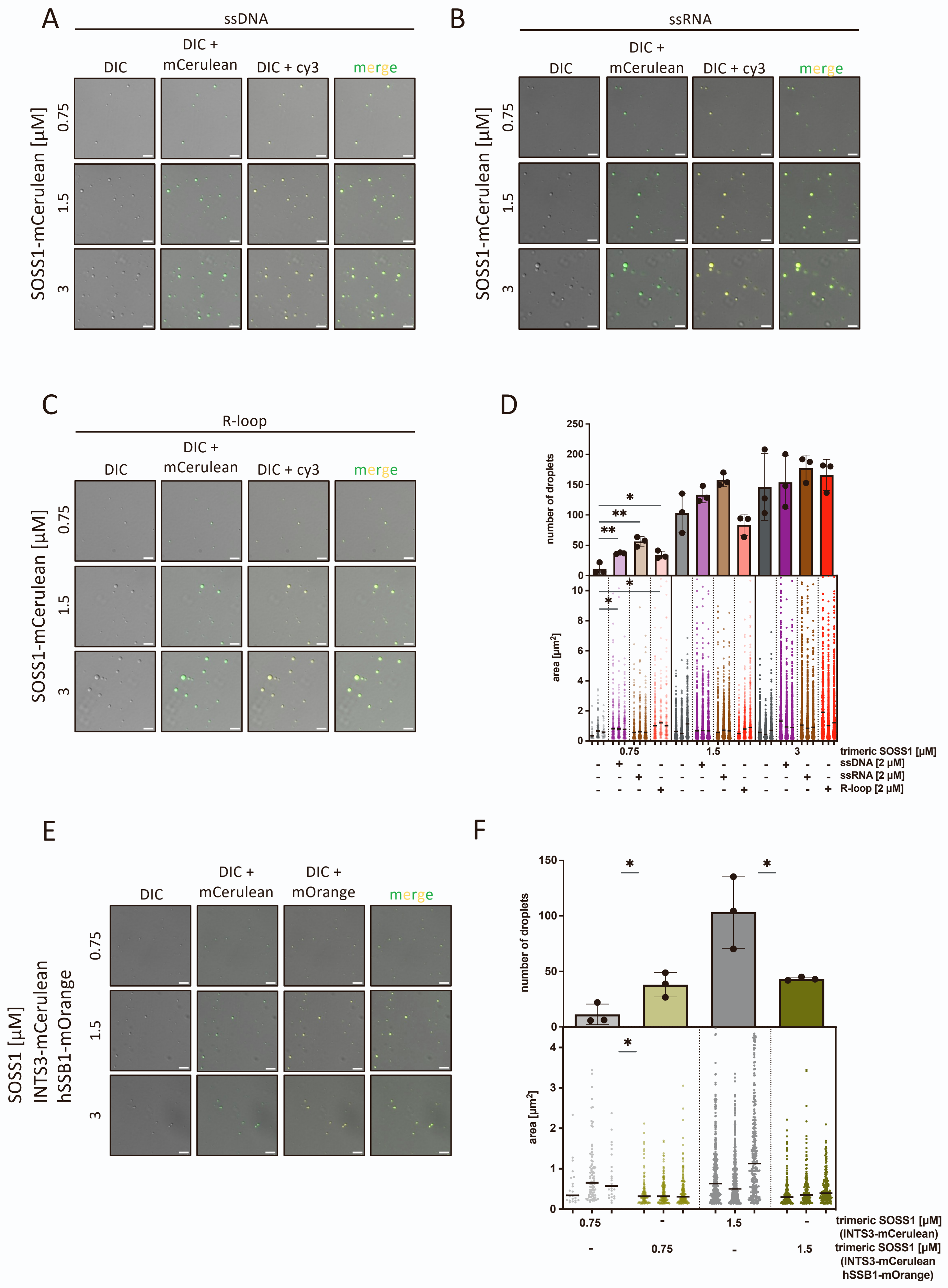

A
ssDNA
DIC
DIC + mCerulean
DIC + cy3
merge
SOSS1-mCerulean [µM]
0.75
1.5
3
B
ssRNA
DIC
DIC + mCerulean
DIC + cy3
merge
SOSS1-mCerulean [µM]
0.75
1.5
3
C
R-loop
DIC
DIC + mCerulean
DIC + cy3
merge
SOSS1-mCerulean [µM]
0.75
1.5
3
D
number of droplets
area [µm²]
trimeric SOSS1 [µM]
ssDNA [2 µM]
ssRNA [2 µM]
R-loop [2 µM]
0.75
1.5
3
E
DIC
DIC + mCerulean
DIC + mOrange
merge
SOSS1 [µM]
INTS3-mCerulean
hSSB1-mOrange
0.75
1.5
3
F
number of droplets
area [µm²]
trimeric SOSS1 [µM] (INTS3-mCerulean)
trimeric SOSS1 [µM] (INTS3-mCerulean hSSB1-mOrange)
0.75
1.5

Figure S14. Phase-separation of trimeric SOSS1 *in vitro*. Related to Figure 5.

A. LLPS experiments determining concentration-dependent phase separation of fluorescently labelled SOSS1 complex in the presence of 5% PEG-8000 and cy3-labelled ssDNA (2µM). Representative images from three experiments are depicted as differential interference contrast (DIC), overlay of DIC and mCerulean, overlay of DIC and cy3, and overlay of all three channels. Scale bar: 5µM.

B. LLPS experiments determining concentration-dependent phase separation of fluorescently labelled SOSS1 complex in the presence of 5% PEG-8000 and cy3-labelled ssRNA (2µM). Representative images from three experiments are depicted as differential interference contrast (DIC), overlay of DIC and mCerulean, overlay of DIC and cy3, and overlay of all three channels. Scale bar: 5µM.

C. LLPS experiments determining concentration-dependent phase separation of fluorescently labelled SOSS1 complex in the presence of 5% PEG-8000 and cy3-labelled R-loop (2µM). Representative images from three experiments are depicted as differential interference contrast (DIC), overlay of DIC and mCerulean, overlay of DIC and cy3, and overlay of all three channels. Scale bar: 5µM.

D. Bar chart (upper panel) represents quantification (n=3) of number of droplets from LLPS experiments shown in (A) and (B). Statistical significance was determined by unpaired *t*-test. $*p \leq 0.05$ and $**p \leq 0.01$. Nested scatter plot (lower panel) represents quantification (n=3) of area of individual droplets from three independent experiments in (A) and (B), with median area determined per dataset. Statistical significance was determined by nested *t*-test. $*p \leq 0.05$.

E. LLPS experiments determining phase separation of trimeric SOSS1 complex labelled on INTS3 (INTS3-mCerulean) and hSSB1 (hSSB1-mOrange). Representative images from three experiments are depicted as differential interference contrast (DIC) and overlay of DIC and mCerulean, DIC and mOrange, and overlay of the three channels. Scale bar: 5µM.

F. Bar chart (upper panel) represents quantification (n=3) of number of droplets from LLPS experiments shown in (D). Statistical significance was determined by unpaired *t*-test. $*p \leq 0.05$. Nested scatter plot (lower panel) represents quantification (n=3) of area of individual droplets from three independent experiments shown in in (D) and (E), with median area determined per dataset. Statistical significance was determined by nested *t*-test. $*p \leq 0.05$.

Figure S15

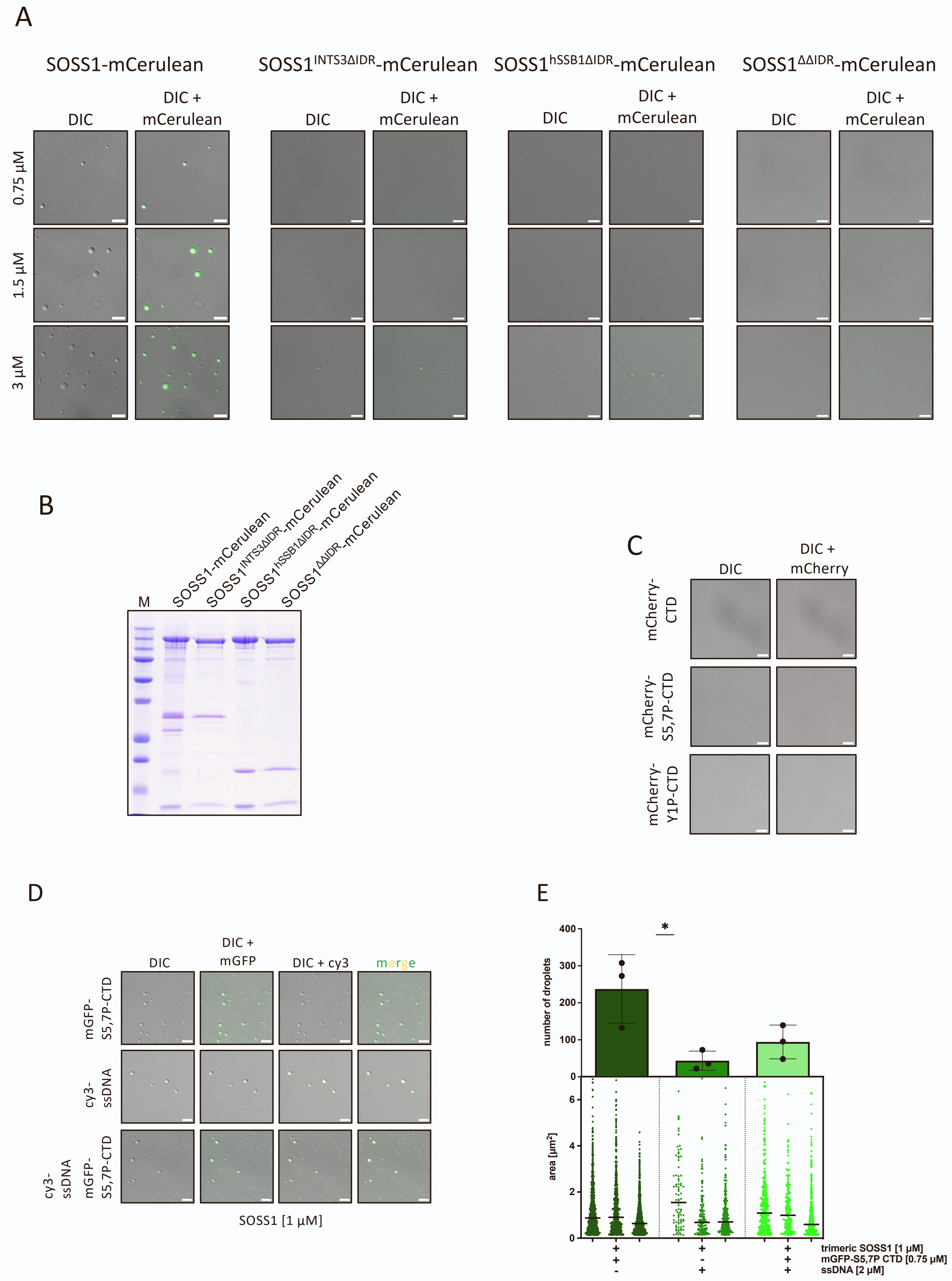
A
SOSS1-mCerulean
SOSS1INTS3ΔIDR-mCerulean
SOSS1hSSB1ΔIDR-mCerulean
SOSS1ΔΔIDR-mCerulean
DIC
DIC + mCerulean
0.75 µM
1.5 µM
3 µM
B
M
SOSS1-mCerulean
SOSS1INTS3ΔIDR-mCerulean
SOSS1hSSB1ΔIDR-mCerulean
SOSS1ΔΔIDR-mCerulean
C
DIC
DIC + mCherry
mCherry-CTD
mCherry-S5,7P-CTD
mCherry-Y1P-CTD
D
DIC
DIC + mGFP
DIC + cy3
merge
mGFP-S5,7P-CTD
cy3-ssDNA
cy3-ssDNA mGFP-S5,7P-CTD
SOSS1 [1 µM]
E
number of droplets
area [µm2]
trimeric SOSS1 [1 µM]
mGFP-S5,7P CTD [0.75 µM]
ssDNA [2 µM]

Figure S15. Further and control LLPS experiments *in vitro.* Related to Figure 5.

A. LLPS experiments determining concentration-dependent phase separation of fluorescently labelled SOSS1 w.t. and SOSS1 mutant variants lacking intrinsically disordered regions found in INTS3 (ΔAA959-1042, labelled INTS3$^{\Delta IDR}$) and hSSB1 (ΔAA140-211, labelled hSSB1$^{\Delta IDR}$) in the presence of 5% PEG-8000. Representative images from three experiments at indicated concentrations are depicted as differential interference contrast (DIC) and overlay of DIC and mCerulean. Scale bar: 5µM.

B. An SDS-PAGE gel depicting purified mCerulean labelled trimeric SOSS1 complexes harbouring either single deletions of the IDRs within INTS3 and hSSB1, respectively, or double deletion.

C. Control LLPS experiments determining phase separation of mCherry-CTD variants (unmodified, phosphorylated on Tyr1 (Y1P-CTD), and modified on Ser5 and Ser7 (S5,7P-CTD) (0.75 µM) in the presence of 5% PEG-8000. Representative images from three experiments are depicted as differential interference contrast (DIC) and overlay of DIC and mCherry. Scale bar: 5µM.

D. LLPS experiments investigating the effect of mGFP-S5,7P-CTD (0,75 µM) and cy3-labelled ssDNA (2µM) on phase separation of unlabelled trimeric SOSS1 complex (1µM). Representative images from three experiments are depicted as DIC, overlay of DIC and mGFP, overlay of DIC and cy3, and overlay of all three channels. Scale bar: 5µM.

E. Bar chart (upper panel) represents quantification (n=3) of number of droplets from LLPS experiments shown in (B). Statistical significance was determined by unpaired *t*-test. *$p \leq 0.05$. Nested scatter plot (lower panel) represents quantification (n=3) of area of individual droplets from three independent experiments shown in (B), with median area determined per dataset. Statistical significance was determined by nested *t*-test.

Figure S16

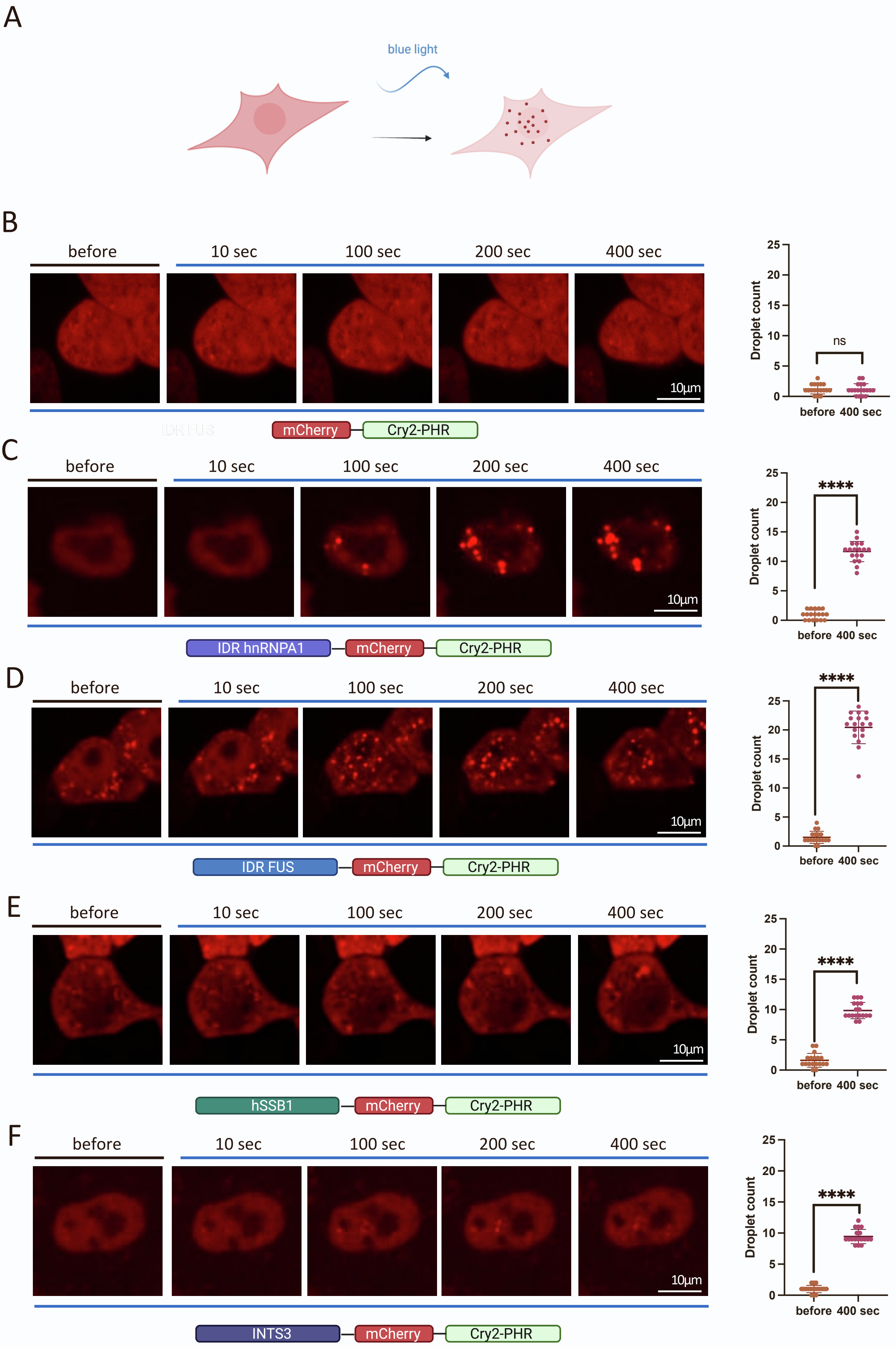
A
blue light
B
before
10 sec
100 sec
200 sec
400 sec
10μm
Droplet count
ns
before
400 sec
mCherry
Cry2-PHR
C
****
IDR hnRNPA1
D
IDR FUS
E
hSSB1
F
INTS3
0
5
10
15
20
25

Figure S16. Trimeric SOSS1 complex phase separates *in vivo*. Related to Figure 6.

A. Diagram showing the optoDroplet strategy. After blue light stimulation, protein fused with Cry2 undergo phase separation and form droplets.

B-F. Light-induced optoDroplet formation of Cry2-mCherry (B), IDR-hnRNAPA1-Cry2-mCherry (C), IDR-FUS-Cry2-mCherry (D), hSSB1-Cry2-mCherry (E) and INTS3-Cry2-mCherry. Cells were imaged at 10 seconds intervals 40 times. Representative images of optoDroplets before and during light induction are shown, specific time points are indicated. Quantification of optoDroplets from 3 independent experiments show values for optoDroplets before and at 400 seconds after light induction. Significance was determined parametric student t- test. ****$p \leq 0.0001$

Figure S17

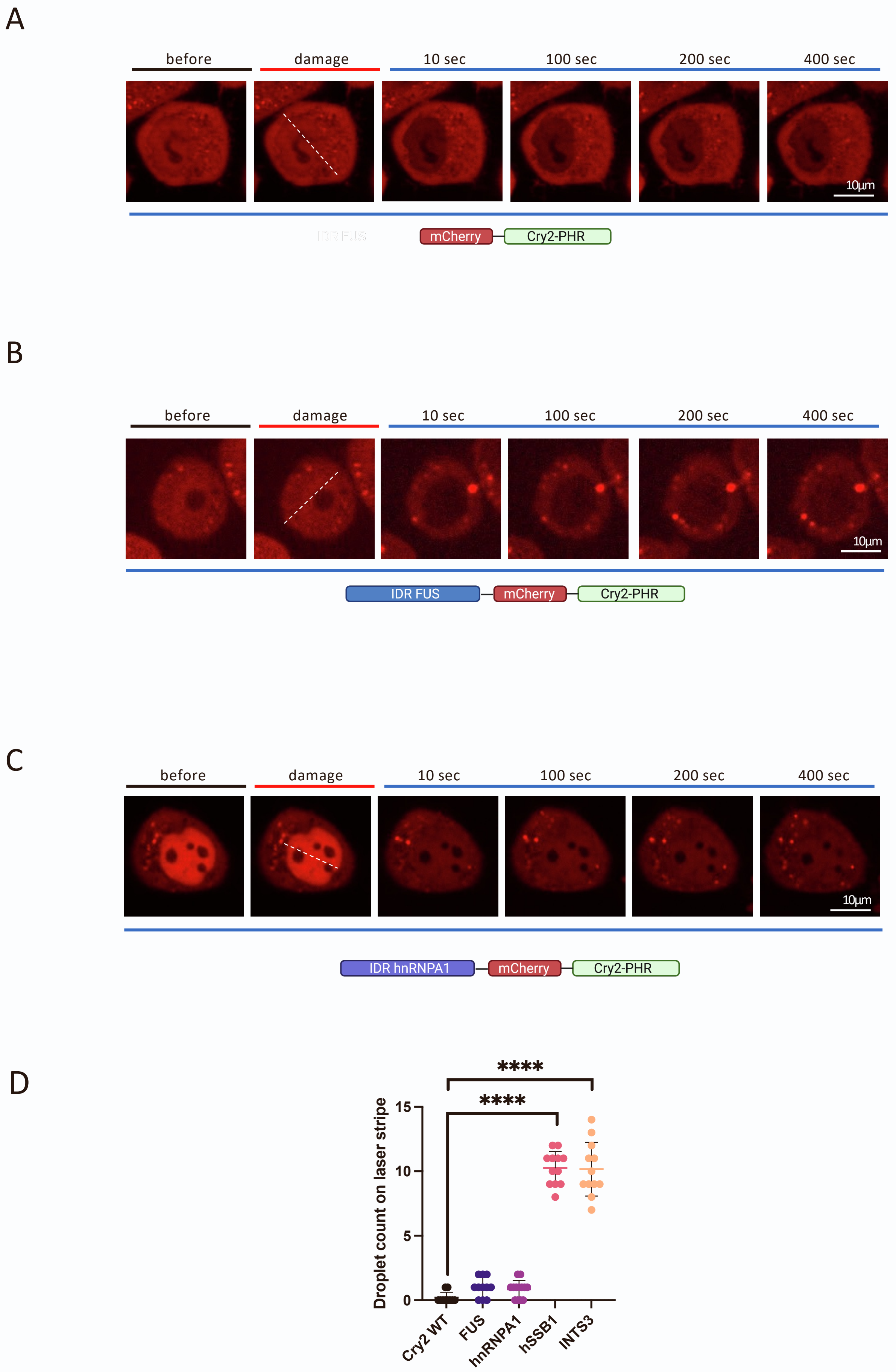

A
before
damage
10 sec
100 sec
200 sec
400 sec
10µm
IDR FUS
mCherry
Cry2-PHR
B
before
damage
10 sec
100 sec
200 sec
400 sec
10µm
IDR FUS
mCherry
Cry2-PHR
C
before
damage
10 sec
100 sec
200 sec
400 sec
10µm
IDR hnRNPA1
mCherry
Cry2-PHR
D
****
****
Droplet count on laser stripe
15
10
5
0
Cry2 WT
FUS
hnRNPA1
hSSB1
INTS3

Figure S17. FUS and hnRNP1 does not phase separates at double strand breaks *in vivo*. Related to Figure 6.

A-C. Damage induced optoDroplet formation in Cry2-mCherry (A), IDR-FUS-Cry2-mCherry (B) and IDR-hnRNPA1-Cry2-mCherry (C) cells. Representative images of optoDroplets before and after laser stripe and during light induction with indicated time points. Position of the laser stripe is marked with dashed white line.

D. Merged quantification graph of (A-C) and Fig 6B.

Figure S18

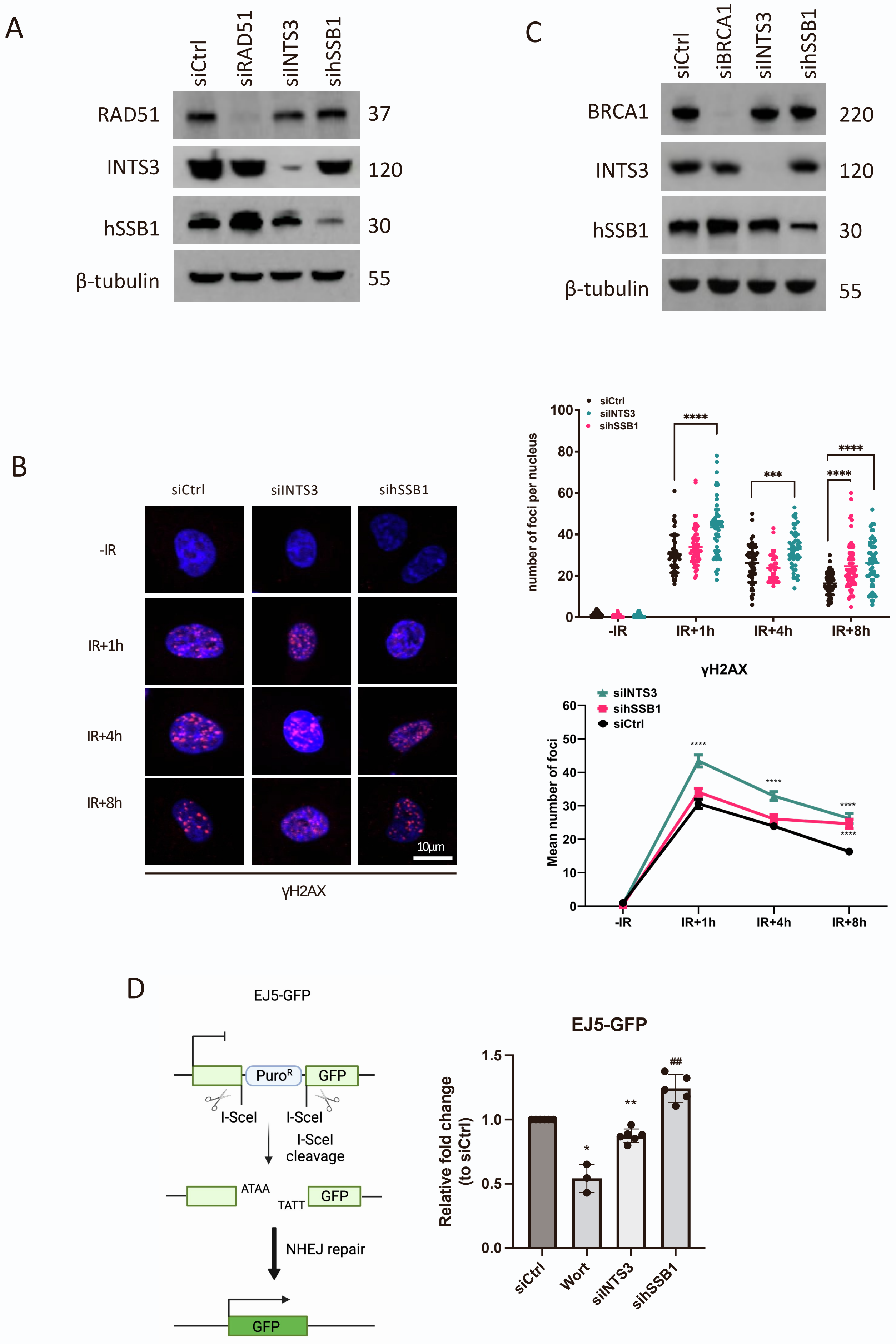

A
siCtrl
siRAD51
siINTS3
sihSSB1
RAD51
INTS3
hSSB1
β-tubulin
37
120
30
55
C
siCtrl
siBRCA1
siINTS3
sihSSB1
BRCA1
INTS3
hSSB1
β-tubulin
220
120
30
55
B
siCtrl
siINTS3
sihSSB1
-IR
IR+1h
IR+4h
IR+8h
10μm
γH2AX
siCtrl
siINTS3
sihSSB1
number of foci per nucleus
100
80
60
40
20
0
****
***
****
****
-IR
IR+1h
IR+4h
IR+8h
γH2AX
siINTS3
sihSSB1
siCtrl
Mean number of foci
60
50
40
30
20
10
0
****
****
****
****
-IR
IR+1h
IR+4h
IR+8h
D
EJ5-GFP
Puro^R
GFP
I-SceI
I-SceI
I-SceI cleavage
ATAA
TATT
GFP
NHEJ repair
GFP
EJ5-GFP
Relative fold change (to siCtrl)
1.5
1.0
0.5
0.0
*
**
##
siCtrl
Wort
siINTS3
sihSSB1

Figure S18. SOSS1 complex is important for DDR. Related to Figure 7.

A. Western blot showing knockdown efficiency in HeLa cells for proteins as indicated.

B. Representative IF images showing the levels of γH2AX in siCtrl, siINTS3 or sihSSB1 cells. Bar chart showing quantification of IF images, ****$p \leq 0.0001$.

C. Western blot showing knockdown efficiency for DRGFP HeLa HR reporter cells corresponding to Fig 7B .

D. Drawing of EJ5-GFP NHEJ reporter strategy. Bar chart shows FACS data checking the efficiency of NHEJ repair in EJ5 HeLa reporter cells. Wortmannin (DNA-PK inhibitor) was used as the positive control. *$p \leq 0.05$, **$p \leq 0.01$, compare with siCtrl significantly decreased; $^{\#\#}\ p \leq 0.01$, compare with siCtrl significantly increased.

Supplementary Table S2. List of oligonucleotides used in *in vitro* work

Name	Sequence 5'-3'	Use
T7F	TAATACGACTCACTATAGGG	Sequencing
T7R	GCTAGTTATTGCTCAGCGG	Sequencing
M13F	CCCAGTCACGACGTTGTAAAACG	Bacmid screening
M13R	AGCGGATAACAATTTCACACAGG	Bacmid screening
FB01	CCTATAACTATTCCGGATTATTCATACCGTC	Sequencing
FB02	CAGGTTCAGGGGGAGGTGTG	Sequencing
pRMS199	TTATCCACTTCCAATGTTATTACTATCTCTT GCTGCTCCTC	Amplification of hSSB1
pRMS490	TACTTCCAATCCAATGCAATGACGACGGA GACCTTTG	Amplification of hSSB1
pRMS202	TACTTCCAATCCAATGCAATGGCAGCAAA CTCTTCAG	Amplification of c9orf80
pRMS203	TTATCCACTTCCAATGTTATTACTATTCTGG GTCAAGGCG	Amplification of c9orf80
pRMS204	TACTTCCAATCCAATGCAATGGAGTTGCA GAAGGGAAA	Amplification of INTS3
pRMS205	TTATCCACTTCCAATGTTATTACTAGTCAC TGTCAGAGCC	Amplification of INTS3
pRMS206	TTGGCAGAAAGTGTTCTGGA	Sequencing of INTS3
pRMS207	AAATGTCGCTGCCTCCAAT	Sequencing of INTS3
pRMS208	CAGAAGGGGAGTGATACGGA	Sequencing of INTS3
pRMS209	TACTTCCAATCCAATCGATGACGACGGAG ACCTTTG	Amplification of INTS6
pRMS210	TTATCCACTTCCAATGTTATTACTAATTGCT ATTAATATGGTTGATC	Amplification of INTS6
pRMS211	GAACAGTTGACAGGTGTGCC	Sequencing of INTS6
pRMS212	TCCAGTCCTTCTTCCCCTCT	Sequencing of INTS6
pRMS213	CAAATGGGGAACTACCAGGA	Sequencing of INTS6
pRMS354	CTCCCACTACCAATGCCGTCACTGTCAGA GCCCAC	Reverse primer for amplification of INTS3 for mCerulean-tagging
pRMS356	CTCCCACTACCAATGCCTCTCTTGCTGCTC CTCCG	Reverse primer for amplification of hSSB1 for mOrange-tagging
pRMS468	TTATCCACTTCCAATGTTATTATTACTTGTA CAGCTCG TCCA	Reverse primer for amplification of mOrange-containing cassettes
pRMS469	TTATCCACTTCCAATGTTATTATTACTACTT GTACAGCTCGTC	Reverse primer for amplification mCerulean-containing cassettes
pRMS563	CTCCCACTACCAATGCCACAGCTCGCTTG GACATG	Reverse primer for amplification of $INTS3^{1-958}$ for mCerulean-tagging
pRMS217	[Cy3]-GACGCTGCCGAATTCTACCAGTGCCTTGC TAGGACATCTTTGCCCACCTGCAGGTTCA CCC	EMSA experiments; Part of the following substrates: RNA:DNA-hybrid, R-loop, bubble, dsDNA
pRMS219		EMSA experiments

	GGGTGAACCTGCAGGTGGGCGGCTGCTC ATCGTAGGTTAGTTGGTAGAATTCGGCAG CGTC	Part of the following substrates: R-loop, bubble
pRMS223	AAAGAUGUCCUAGCAAGGCAC	EMSA experiments<br>Part of the following substrates: RNA:DNA-hybrid, R-loop
pRMS391	GGGTGAACCTGCAGGTGGGCAAAGATGT CCTAGCAAGGCACTGGTAGAATTCGGCAG CGTC	EMSA experiments<br>Part of the following substrates: dsDNA
pRMS564	CTCCCACTACCAATGCCTCCAGTGGTAGG CTGGGA	Reverse primer for amplification of hSSB$^{1\text{-}139}$ for mOrange-tagging, Sequencing of hSSB1
pRMS630	[Cy3]-GTGCCTTGCTAGGACATCTTT	EMSA experiments
pRMS726	TTCAGTGAGCCAAACCCAGAGGCAAGCA CCCAGCAGGCACCCAAC	Forward primer introducing mutation hSSB1^{Y115A}
pRMS727	GTTGGGTGCCTGCTGGGTGCTTGCCTCTG GGTTTGGCTCACTGAA	Reverse primer introducing mutation hSSB1^{Y115A}
pRMS728	ATTGGAGAATTCTGTATGGTTGCTTCTGAG GTTCCTAACTTCAGT	Forward primer introducing mutation hSSB1^{Y102A}
pRMS729	ACTGAAGTTAGGAACCTCAGAAGCAACC ATACAGAATTCTCCAAT	Reverse primer introducing mutation hSSB1^{Y102A}
pRMS730	TTCAAAGGTTGTCTGACACTAGCTACTGG CCGTGGGGGTGATCTG	Forward primer introducing mutation hSSB1^{Y85A}
pRMS731	CAGATCACCCCCACGGCCAGTAGCTAGTG TCAGACAACCTTTGAA	Reverse primer introducing mutation hSSB1^{Y85A}
pRMS732	ATTATCCGGCTCACCAAAGGGGCAGCTTC AGTTTTCAAAGGTTGT	Forward primer introducing mutation hSSB1^{Y74A}
pRMS733	ACAACCTTTGAAAACTGAAGCTGCCCCTT TGGTGAGCCGGATAAT	Reverse primer introducing mutation hSSB1^{Y74A}
pRMS831	TTATCCACTTCCAATGTTATTAACAGCTCG CTTGGACATG	Reverse primer for amplification of INTS3$^{1\text{-}958}$
pRMS832	TTATCCACTTCCAATGTTATTATCCAGTGG TAGGCTGGGA	Reverse primer for amplification of hSSB1$^{1\text{-}139}$

Supplementary Table S3. List of gene blocks used in *in vitro* work

Name	Backbone	ORF(s)
pMS1-1	H6-mCerulean	INTS3
pMS1-2	H6-mCerulean	INTS3$^{1\text{-}958}$
pMS1-3	H6-mOrange	hSSB1
pMS1-4	H6-mOrange	hSSB1$^{1\text{-}139}$
pMS1-5	438B	INTS3-mCerulean
pMS1-6	438B	INTS3$^{1\text{-}958}$-mCerulean
pMS1-7	438B	hSSB1-mOrange
pMS1-8	438B	hSSB1$^{1\text{-}139}$-mOrange
pMS1-9	438C	INTS6
pMS1-10	438B	c9orf80

pMS1-11	438B	hSSB1
pMS1-12	438B	hSSB1$^{1-139}$
pMS1-13	438B	INTS3$^{1-958}$
pMS1-14	438B	INTS3
pMS1-15	438B	INTS3, hSSB1, c9orf80
pMS1-16	438B	INTS3$^{1-958}$-mCerulean, hSSB1, c9orf80
pMS1-17	438B	INTS3-mCerulean, hSSB1, c9orf80
pMS1-18	438B	INTS3$^{1-958}$, c9orf80
pMS1-19	438B	INTS3, c9orf80
pMS1-20	438B	INTS3$^{1-958}$-mCerulean, c9orf80
pMS1-21	438B	INTS3-mCerulean, c9orf80
pMS1-22	438B	INTS3$^{1-958}$-mCerulean, hSSB1$^{1-139}$, c9orf80
pMS1-23	438B	INTS3-mCerulean, hSSB1$^{1-139}$, c9orf80
pMS1-24	438B	INTS3$^{1-958}$, hSSB1, c9orf80
pMS1-25	438B	INTS3$^{1-958}$, hSSB1$^{1-139}$, c9orf80
pMS1-26	438B	INTS3, hSSB1$^{1-139}$, c9orf80
pMS1-27	438B	INTS3-mCerulean, hSSB1-mOrange, c9orf80
pMS1-28	438B	INTS3, INTS6, hSSB1, c9orf80
pMS1-29	2BT	hSSB1
pMS1-30	2BT	hSSB1^{Y74A}
pMS1-31	2BT	hSSB1^{Y85A}
pMS1-32	2BT	hSSB1^{Y102A}
pMS1-33	2BT	hSSB1^{Y115A}
pMS1-34	2BT	hSSB1^{YY102,115AA}
pMS1-35	438B	hSSB1^{Y102A}
pMS1-36	438B	hSSB1^{Y115A}
pMS1-37	438B	hSSB1^{YY102,115AA}
pMS1-38	438B	INTS3, hSSB1^{Y102A}
pMS1-39	438B	INTS3, hSSB1^{Y115A}
pMS1-40	438B	INTS3, hSSB1^{YY102,115AA}
pMS1-41	438B	INTS3, hSSB1^{Y102A}, c9orf80
pMS1-42	438B	INTS3, hSSB1^{Y115A}, c9orf80
pMS1-43	438B	INTS3, hSSB1^{YY102,115AA}, c9orf80

Supplementary Table S4. List of primers used for mutagenesis in this work

Primer Name	Primer Sequence (5'-3')
Y102A.GCC.FOR	GAATTCTGTATGGTTGCCTCTGAGGTTCCTAAC
Y102A-GCC.REV	TCCAATCTTCTGCAGATCA
Y115A-GCC.FOR	GAGCCAAACCCAGAGGCCAGCACCCAGCAGGCACCC
Y115A.GCC-REV	ACTGAAGTTAGGAACCTCAGAATAAACCATACAGAATTC

Supplementary Table S5: List of primers used for Gibson cloning in this work

Primer Name	Primer Sequence (5'-3')
OPTO_1_CLO_FWD	cccggaggagcagcaagagaggaggaatggtgtctaaagg
OPTO_1_CLO_REV	ccacaccctaactgacacacattccacagctgcattaatg
OPTO_1_ACL_FWD	cattaatgcagctgtggaatgtgtgtcagttagggtgtgg
OPTO_1_ACL_REV	acaaaggtctccgtcgtcatggtagctccggatccactgtc
OPTO_hSSB1_FWD	acagtggatccggagctaccatgacgacggagacctttgtgaag
OPTO_hSBB1_REV	cctttagacaccattcctcctctcttgctgctcctccggg
OPTO_3_CLO_FWD	cattaatgcagctgtggaatgtgtgtcagttagggtgtgg
OPTO_3_CLO_REV	tttcccttctgcaactccatggtagctccggatccactgtc
OPTO_3_ACL_FWD	cagtgggctctgacagtgacggaggaatggtgtctaaagg
OPTO_3_ACL_REV	ccacaccctaactgacacacattccacagctgcattaatg
OPTO_INTS3_FWD	acagtggatccggagctaccatggagttgcagaagggaaaag
OPTO_INTS3_REV	cctttagacaccattcctccgtcactgtcagagcccactg
INTS3_DELTA_RIGHT_rev	GTGCCAGGCGTTGAAAAGATTAG
INTS3_DELTA_RIGHT_fwd	agcatgtccaagcgagctgtGGAGGAATGGTGTCTAAAGG
INTS3_DELTA_LEFT_fwd	CCGCCAATCTCCGGTCGCTA
INTS3_DELTA_LEFT_rev	cctttagacaccattcctccACAGCTCGCTTGGACATGCTG
hSSB1_DELTA_RIGHT_rev	CAGCTCACTCAAAGGCGGTAATAC
hSSB1_DELTA_RIGHT_fwd	cttcccagcctaccactggaGGAGGAATGGTGTCTAAAGGC
hSSB1_DELTA_LEFT_fwd	GATTCTGTGGATAACCGTATTACCG
hSSB1_DELTA_LEFT_rev	cctttagacaccattcctccTCCAGTGGTAGGCTGGGAAG
OPTO_Y102A hSSB1_FWD	acagtggatccggagctaccatgacgacggagacctttgtgaag
OPTO_ Y102A hSBB1_REV	cctttagacaccattcctcctctcttgctgctcctccggg
OPTO_Y115A hSSB1_FWD	acagtggatccggagctaccatgacgacggagacctttgtgaag
OPTO_ Y115A hSBB1_REV	cctttagacaccattcctcctctcttgctgctcctccggg
OPTO_Y102A&Y115A hSSB1_FWD	acagtggatccggagctaccatgacgacggagacctttgtgaag
OPTO_ Y102A&Y115A hSBB1_REV	cctttagacaccattcctcctctcttgctgctcctccggg
